# Toward Structurally Novel and Metabolically Stable HIV-1 Capsid-Targeting Small Molecules

**DOI:** 10.3390/v12040452

**Published:** 2020-04-16

**Authors:** Sanjeev Kumar V. Vernekar, Rajkumar Lalji Sahani, Mary C. Casey, Jayakanth Kankanala, Lei Wang, Karen A. Kirby, Haijuan Du, Huanchun Zhang, Philip R. Tedbury, Jiashu Xie, Stefan G. Sarafianos, Zhengqiang Wang

**Affiliations:** 1 Center for Drug Design, College of Pharmacy, University of Minnesota, Minneapolis, MN 55455, USA; vvsanjeev99@googlemail.com (S.K.V.V.); rsahani@umn.edu (R.L.S.); kankanalajk1@gmail.com (J.K.); leiwang@dlut.edu.cn (L.W.); jxie@umn.edu (J.X.); 2 Department of Molecular Microbiology and Immunology, University of Missouri School of Medicine, Christopher S. Bond Life Sciences Center, Columbia, MO 65211, USA; mcc6x2@mail.missouri.edu; 3 Laboratory of Biochemical Pharmacology, Department of Pediatrics, Emory University School of Medicine, Atlanta, GA 30322, USA; karen.kirby@emory.edu (K.A.K.); haijuan.du@emory.edu (H.D.); huanchun.zhang@emory.edu (H.Z.); philip.tedbury@emory.edu (P.R.T.); stefanos.sarafianos@emory.edu (S.G.S.)

**Keywords:** HIV-1, capsid-targeting antivirals, PF74, metabolic stability

## Abstract

HIV-1 capsid protein (CA) plays an important role in many steps of viral replication and represents an appealing antiviral target. Several CA-targeting small molecules of various chemotypes have been studied, but the peptidomimetic **PF74** has drawn particular interest due to its potent antiviral activity, well-characterized binding mode, and unique mechanism of action. Importantly, **PF74** competes against important host factors for binding, conferring highly desirable antiviral phenotypes. However, further development of **PF74** is hindered by its prohibitively poor metabolic stability, which necessitates the search for structurally novel and metabolically stable chemotypes. We have conducted a pharmacophore-based shape similarity search for compounds mimicking **PF74**. We report herein the analog synthesis and structure-activity relationship (SAR) of two hits from the search, and a third hit designed via molecular hybridization. All analogs were characterized for their effect on CA hexamer stability, antiviral activity, and cytotoxicity. These assays identified three active compounds that moderately stabilize CA hexamer and inhibit HIV-1. The most potent analog (**10**) inhibited HIV-1 comparably to **PF74** but demonstrated drastically improved metabolic stability in liver microsomes (31 min vs. 0.7 min t_1/2_). Collectively, the current studies identified a structurally novel and metabolically stable **PF74**-like chemotype for targeting HIV-1 CA.

## 1. Introduction

Human immunodeficiency virus 1 (HIV-1) encodes a Gag polyprotein which contains multiple protein domains for viral assembly and release: matrix (p17 MA), capsid (p24 CA), nucleocapsid (p7 NC), p6 and spacer peptides Sp1 and Sp2 [1]. Gag polyproteins assemble to form the immature viral capsid core, which upon protease cleavage rearranges into a fullerene-shaped mature capsid core [2] comprising approximately 250 CA hexamers and exactly 12 asymmetrically-distributed CA pentamers [3]. CA plays essential roles in viral assembly and multiple events during viral replication [4,5]. CA-CA interactions drive the assembly and disassembly of viral capsid, and capsid core stability is important for reverse transcription, nuclear entry, and cloaking of the viral DNA product from host nucleic acid sensing mechanisms in the cytosol [6]. CA interacts with many cellular factors [7,8], including TRIM5α [9,10], cleavage and polyadenylation specific factor 6 (CPSF6) [11,12] nucleoporins 153 [13,14,15] and 358 [16,17,18] (NUP153, NUP358), MxB [19,20], and Cyclophilin A (CypA) [21,22,23]. These interactions enable early viral replication steps, including uncoating, cytoplasmic trafficking, reverse transcription, nuclear transport, site of integration, and the evasion of innate immunity [24]. Hence, CA-targeting small molecules could confer both early and late stage antiviral phenotypes by perturbing the stability of viral capsid core and interfering with important CA-host interactions.

**Figure 1:**
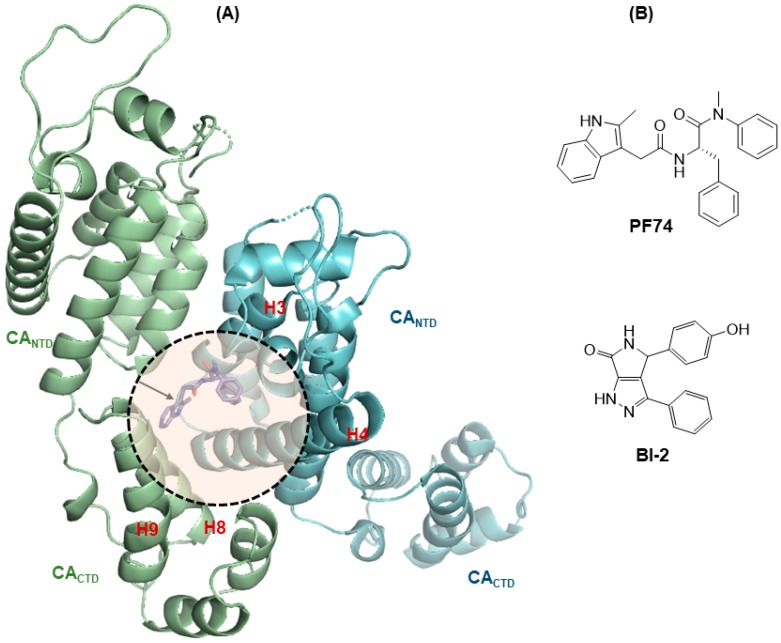
Structure of HIV-1 capsid protein (CA) and a key CA ligand binding pocket. (**A**) The structure of CA dimer (Protein Data Bank code: 4XFZ [25]). The pocket formed around H3 and H4 of the CA_NTD_ (cyan), and H8 and H9 of the adjacent CA_CTD_ (green) accommodates the binding of multiple ligands, including host factors Nup153 and CPSF6, and small molecules **PF74** and **BI-2**. Shown in the pocket is **PF74** (pointed to with an arrow); (**B**) chemical structures of **PF74** and **BI-2**.

The CA structure [25,26,27] is mostly helical (Figure 1A): it comprises 7 helices at the N-terminal domain (CA_NTD_) and 4 helices at the C-terminal domain (CA_CTD_). Interactions between one CA_NTD_ and the CA_NTD_ and CA_CTD_ of an adjacent monomer form the molecular basis of stable hexameric lattice and virus-host interactions. Previous structural studies with CA-bound compounds have revealed a small molecule binding sites [28], such as the particularly interesting **PF74** binding pocket. This pocket is formed primarily by H3 and H4 of the CA_NTD_ (cyan), and the H8 and H9 of the CA_CTD_ of an adjacent monomer (green) (Figure 1A). In addition to accommodating the binding [25,29,30] of **PF74** and **BI-2**, a smaller CA-targeting molecule (Figure 1B), the same pocket is also used by important cellular factors, including [29,30]: Nup153, a nucleoporin important for nuclear transport of viral preintegration complexes (PICs); and CPSF6 which transports HIV-1 PICs to transcriptionally active chromatin [31]. **PF74** is a well-characterized [32] peptidomimetic built around a phenylalanine core, and capped with an aniline moiety at the carboxylate end and an indole-3-acetic acid at the amino end (Figure 1B). All three components, the core, the aniline moiety (right) and the indole acid moiety (left), provide key molecular interactions for the binding of **PF74 [25]**. Consistent with the binding site and mode, **PF74** displayed a concentration-dependent bimodal mechanism [32,33] of action: at lower concentrations it competes against host factors including CPSF6 and NUP153 to affect nuclear entry; and at higher concentrations it blocks uncoating and reverse transcription, presumably by altering inter-hexamer interactions [25].

**Figure 2:**
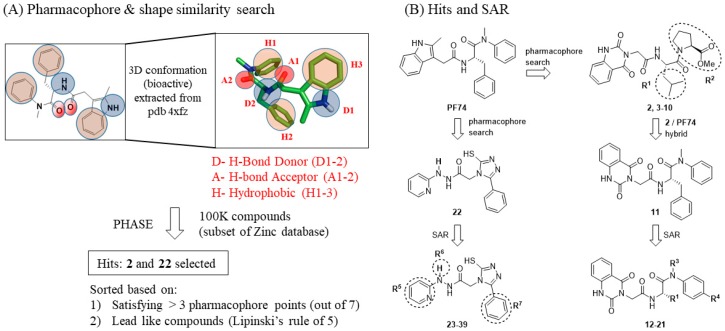
Generation of novel compounds targeting the **PF74** binding pocket. (**A**) Initial hits generated via a pharmacophore and shape similarity search; (**B**) SAR of two selected hits (**2** and **22**), as well as a new hit (**11**) from the hybridization between **2** and **PF74**. Compounds **3–10** are variants of **2**, with modifications in the circled regions.

Despite the attractive antiviral profile and the unique bimodal mechanism of action, **PF74** is not a viable drug candidate due primarily to its prohibitively poor metabolic stability [34]. In human liver microsomes (HLMs), the half-life (t_1/2_) of **PF74** was less than 1 min [35,36]. This major deficiency strongly necessitates targeted efforts to search for structurally novel and metabolically stable small molecules capable of binding to the **PF74** binding pocket. Along this line, the Zhan and Liu group recently reported **PF74**-like compounds where an easier and synthetically more accessible 1,2,3-triazole ring was substituted for the indole moiety [35,36]. However, such compounds were considerably (>10-fold) less potent than **PF74** in antiviral assays and were essentially as unstable in HLMs [35,36]. Our current work features a pharmacophore-based shape similarity search approach based on **PF74** (Figure 2A). Molecular similarity [37,38] is an important concept in medicinal chemistry and drug discovery. Hit generation using similarity search is based on the premise that similar chemical structure confers similar biological activity. In our search, we extracted the 3D conformation of **PF74** from reported co-crystal structure (PDB: 4XFZ [25]) and defined 7 pharmacophore points (Figure 2A) according to its mode of binding, including two H-bond donors (D1 and D2), two H-bond acceptors (A1 and A2), as well as three hydrophobic moieties (H1, H2 and H3). This 3D pharmacophore was then used to screen against a subset of the ZINC database [39] (100K compounds) using the program PHASE [40,41]. Hit ranking was based on the number of pharmacophore points satisfied and the lead like properties as predicted by the Lipinski’s rule of 5 [42]. From this similarity search we selected two hits (**2** and **22**) for analog synthesis and SAR (Figure 2B). Molecular hybridization [43] between hit **2** and **PF74** also generated hit **11**, which was also subjected to analog synthesis (Figure 2B). We report herein the analog synthesis and SAR on all three hits. In the end, our best compound inhibited HIV-1 with an EC_50_ of 1.6 μM, and more important exhibited a half-life (t_1/2_) 44-fold longer than **PF74**.

## 2. Materials and Methods

**Scheme 1:**
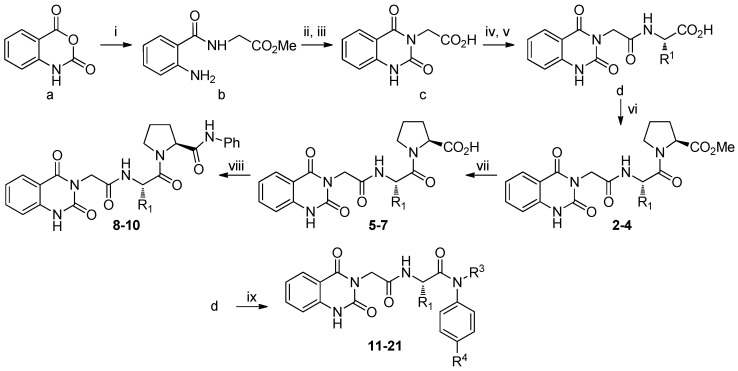
Synthetic Strategy for Analogs **2**–**21**. Reagents and conditions: (i) glycine methyl ester hydrochloride, Et_3_N, DMF, rt, 12 h; (ii) ethyl chloroformate, pyridine, rt, 12 h; (iii) NaOH, MeOH/H_2_O, 60 °C, 12 h; (iv) *L*-amino acid methyl ester hydrochloride, HATU, DIPEA, DMF, rt, 12 h; (v) LiOH, MeOH/H_2_O, rt, 12 h; (vi) *L*-proline methyl ester hydrochloride, PyAOP, DIPEA, DMF, rt, 12 h; (vii) LiOH, MeOH/H2O, rt, 12 h/60 °C, 3 h; (viii) amine, PyAOP, DIPEA, DMF, rt, 12 h; (ix) amine, HATU, DIPEA, DMF, rt, 12 h.

**Scheme 2:**
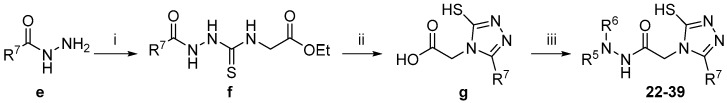
Synthetic Strategy for Analogs **22**–**39**. Reagents and conditions: (i) ethyl isothiocyanatoacetate, ACN, 40 °C, 3 h; (ii) NaOH, H_2_O, 100 °C, 3 h; (iii) Hydrazine, HATU, DIPEA, DMF, rt, 24 h.

All analogs were synthesized using procedures described in Scheme 1 and Scheme 2, fully characterized with ^1^H NMR, ^13^C NMR, and HRMS, and displayed a purity of ≥95% as determined by HPLC. Detailed synthetic procedures and compound characterization data are included in Supplemental Materials.

The general synthetic strategy for major analogs (**1**–**39**) tested in this work is described here (Scheme 1 and Scheme 2) and the synthesis for others is outlined in supporting information (Schemes S1 and S2). Commercially available 1*H*-benzo[*d*] [1,3] oxazine-2,4-dione (**a**) was reacted with glycine methyl ester hydrochloride in the presence of Et_3_N in DMF to yield intermediate (**b**). Further treatment of intermediate (**b**) with ethyl chloroformate in pyridine and then with NaOH in MeOH/H_2_O afforded the carboxylic acid intermediate (**c**). A subsequent amide coupling of carboxylic acid intermediate (**c**) with different *L*-amino acid methyl ester hydrochlorides in the presence of HATU and DIPEA in DMF furnished acid intermediates (**d**) after hydrolysis under LiOH in MeOH/H_2_O. To synthesize varied hit **2** analogs, carboxylic acid intermediates (**d**) were further coupled with *L*-proline methyl ester hydrochloride in the presence of PyAOP and DIPEA to produce compounds **2**–**4**, which upon hydrolysis afforded **5**–**7**. Another coupling with various amines delivered analogs **8**–**10**. Likewise, acid intermediates (**d**) were treated with various anilines using HATU and DIPEA in DMF to produce hit **11** analogs **11**–**21**.

The synthesis of hit **22** analogs **22**–**39** was achieved with a straightforward approach described in Scheme 2. Hydrazides (**e**) reacted with ethyl isothiocyanatoacetate in ACN to give thiosemihydrazides (**f**) that were converted to triazole acid derivatives (**g**) through in situ cyclization and hydrolysis when treated with aqueous NaOH. Amide coupling of these triazole acid derivatives (**g**) with various amines in the presence of HATU and DIPEA in DMF resulted in analogs **22**–**39**.

### 2.1. Biology Cells

TZM-GFP cells are a modified version of TZM-bl cells and contain an integrated nlsGFP reporter gene under the transcriptional control of the HIV-1 long terminal repeat (LTR) [44,45]. TZM-GFP cells were kindly provided by Dr. Marc Johnson (University of Missouri-Columbia, Columbia, MO) and cultured in DMEM supplemented with 10% fetal bovine serum (FBS; Hyclone). HEK293-FT cells were also cultured in DMEM supplemented with 10% FBS. MT-2 cells were grown in RPMI supplemented with 10% heat-inactivated FBS. All cells were grown at 37 °C and maintained in humidified atmosphere containing 5% CO_2_.

### 2.2. Method Details

#### 2.2.1. Thermal Shift Assays (TSAs) to Screen Compounds for Effect on HIV-1 CA Hexamer Stability

Compounds were screened for their effect on CA hexamer stability using purified covalently-crosslinked hexameric CA^A14C/E45C/W184A/M185A^ (CA121). CA121 cloned in a pET11a expression plasmid was provided by Dr. Owen Pornillos (University of Virginia, Charlottesville, VA). Protein was expressed in *E. coli* BL21(DE3)RIL and purified as reported previously [26]. The TSA was conducted as previously described [46,47,48] using the PikoReal Real-Time PCR (Thermo Fisher Scientific, Waltham, MA, USA) or the QuantStudio 3 Real-Time PCR (Thermo Fisher Scientific) systems. Each reaction contained 10 µL of 15 µM CA121 (7.5 µM final concentration) in 50 mM sodium phosphate buffer (pH 8.0), 10 µL of 2× Sypro Orange Protein Gel Stain (Life Technologies, Carlsbad, CA, USA) in 50 mM sodium phosphate buffer (pH 8.0) and 0.2 µL of DMSO (control) or compound. Compounds were tested at a final concentration of 20 µM. The plate was heated from 25 to 95 °C with a heating rate of 0.2 °C/10 s. The fluorescence intensity was measured with an Ex range of 475–500 nm and Em range of 520–590 nm. The differences in the melting temperature (ΔTm) of CA hexamer in DMSO (T0) verses in the presence of compound (Tm) were calculated using the following formula: ΔTm = Tm − T0.

#### 2.2.2. Virus Production

The wild-type laboratory HIV-1 strain, HIV-1_NL4-3_ [49], was produced using a pNL4-3 vector that was obtained through the NIH AIDS Reagent Program. HIV-1_NL4-3_ was generated by transfecting HEK 293FT cells in a T75 flask with 10 µg of the pNL4-3 vector and FuGENE^®^ HD Transfection Reagent (Promega, Madison, WI, USA). Supernatant was harvested 48–72 h post-transfection and transferred to MT2 cells for viral propagation. Virus was harvested when syncytia formation was observed, which took 3–5 days. The viral supernatant was then concentrated using 8% *w/v* PEG 8000 overnight at 4 °C, followed by centrifugation for 40 min at 3500 rpm. The resulting viral-containing pellet was concentrated 10-fold by resuspension in DMEM without FBS and stored at −80 °C.

#### 2.2.3. Anti-HIV-1 and Cytotoxicity Assays

Anti-HIV-1 activity of PF74 and related analogs was examined in TZM-GFP cells. The potency of HIV-1 inhibition by a compound was based on its inhibitory effect on viral LTR-activated GFP expression compared with that of compound-free (DMSO) controls. Briefly, TZM-GFP cells were plated at density of 1 × 10^4^ cells per well in a 96-well plate. 24 h later, media was replaced with increasing concentrations of compound. 24 h post treatment, cells were exposed to an HIV-1 strain (MOI = 1). After incubation for 48 h, anti-HIV-1 activity was assessed by counting the number of GFP positive cells on a Cytation^TM^ 5 Imaging Reader (BioTek, Winooski, VT, USA) and 50% effective concentration (EC_50_) values were determined.

Cytotoxicity of each compound was also determined in TZM-GFP cells. Cells were plated at a density of 1 × 10^4^ cells per well in a 96-well plate and were continuously exposed to increasing concentrations of a compound for 72 h. The number of viable cells in each well was determined using a Cell Proliferation Kit II (XTT), and 50% cytotoxicity concentration (CC_50_) values were determined. All the cell-based assays were conducted in duplicate of at least two independent experiments and the average values were determined.

For calculation of EC_50_ and CC_50_ dose response curves, values were plotted in GraphPad Prism 5 and analyzed with the log (inhibitor) vs. normalized response—Variable slope equation. Final values were calculated in each independent assay and the average values were determined. Statistical analysis (calculation of standard deviation) was performed using Microsoft Excel.

### 2.3. Microsomal Stability Assay

The in vitro microsomal stability assay was conducted in duplicate in mouse and human liver microsomal systems, which were supplemented with nicotinamide adenine dinucleotide phosphate (NADPH) as a cofactor. Briefly, a compound (1 µM final concentration) was pre-incubated, in the absence or presence of 0.5 µM Cobicistat (CYP 3A inhibitor, purchased from medchemexpress.com and verified with LCMS), with the reaction mixture containing liver microsomal protein (0.5 mg/mL final concentration) and MgCl_2_ (1 mM final concentration) in 0.1 M potassium phosphate buffer (pH 7.4) at 37 °C for 15 min. The reaction was initiated by addition of 1 mM NADPH, followed by incubation at 37 °C. A negative control was performed in parallel in the absence of NADPH to measure any chemical instability or non-NADPH dependent enzymatic degradation for each compound. At various time points (0, 5, 15, 30 and 60 min), 1 volume of reaction aliquot was taken and quenched with 3 volumes of acetonitrile containing an appropriate internal standard and 0.1% formic acid. The samples were then vortexed and centrifuged at 15,000 rpm for 5 min at 4 °C. The supernatants were collected and analyzed by LC-MS/MS to determine the in vitro metabolic half-life (t_1/2_).

### 2.4. Molecular Modeling

Molecular modeling was performed using the Schrödinger small molecule drug discovery suite 2019-1 [50]. The crystal structure of native HIV-1 capsid protein in complex with PF74 [25] was retrieved from the protein data bank (PDB code: 4XFZ [25]). The above structure was analyzed using Maestro [51] (Schrödinger; LLC: New York, NY, USA) and subjected to a docking protocol that involves several steps including preparing protein of interest, grid generation, ligand preparation, and docking. The crystal structure was refined using the protein preparation wizard [52] (Schrödinger; LLC: New York, NY, USA.) wherein missing hydrogen atoms, side chains, and loops were added using Prime and minimized using the OPLS 3e force field [53] to optimize the hydrogen bonding network and converge the heavy atoms to an rmsd of 0.3 Å. The receptor grid generation tool in Maestro (Schrödinger; LLC: New York, NY, USA) was used to define an active site around the native ligand PF74 to cover all the residues within 12 Å. All the compounds were drawn using Maestro and subjected to Lig Prep to generate conformers, possible protonation at pH of 7 ± 2 that serves as an input for docking process. All the dockings were performed using Glide XP [54] (Glide: version 8.2) with the van der Waals radii of nonpolar atoms for each of the ligands were scaled by a factor of 0.8. The solutions were further refined by post docking and minimization under implicit solvent to account for protein flexibility. The residue numbers of HIV-1 capsid protein used in the discussion and the figures were based on the native HIV-1 capsid protein.

## 3. Results

All analogs synthesized for each hit were assessed first with a biophysical protein stability assay, or thermal shift assay, where the effect of the compound was measured by the change in protein melting point compared to the DMSO control (ΔTm). A positive value in ΔTm indicates a stabilizing effect on the protein and a negative value indicates a destabilizing effect. Of note, the target CA protein is in a covalently crosslinked hexameric state. Thus, the ΔTm values likely reflect local changes that may affect stabilization and exclude inter-hexamer effects, which are important correlates of overall capsid core stability. To simplify presentation of the data, we refer to the effects of these compounds as stabilization or destabilization of “CA hexamer.” All compounds were then screened at 20 µM against HIV-1 in a cell-based assay to determine antiviral activity. Compounds demonstrating significant inhibition were further tested in a dose-response fashion for antiviral EC_50_ values. All compounds were tested for cytotoxicity either by screening at 100 or 50 µM, or determination of CC_50_ values. The benchmark compound **PF74** was resynthesized and tested in these assays (1, ΔTm = 7.4 °C, EC_50_ = 0.61 μM, CC_50_ = 76 μM). The best compound (**10**) was also tested for liver microsomal stability. Molecular modeling was performed for a few selected compounds to help understand the SAR.

### 3.1. SAR of Hit 2 (R^1^ and R^2^)

Hit **2** showed only a weak CA hexamer stabilizing effect (ΔTm = 0.5 °C) with no significant antiviral activity at 20 μM and no cytotoxicity at 100 μM (Table 1). Removing the isopropyl group at R^1^ (compound **3**) resulted in the complete loss of the CA hexamer stabilizing effect, whereas replacing it with an indole-7-methyl (compound **4**) did not yield significant differences in all three assays. When the ester was hydrolyzed to acid (compounds **5**–**7**), only the analog with an indole-7-methyl at R^1^ (compound **5**) weakly stabilized CA hexamer (ΔTm = 0.8 °C); no such effect was observed when R^1^ was a benzyl methyl thioether (compound **6**) or a bulky alkyl (compound **7**). Amidation of the acid with an aniline yielded more structurally elaborate analogs **8**–**10**. Of these, the analogs with a benzyl methyl thioether (compound **8**) or an indole-7-methyl (compound **9**) at R^1^ showed only weak CA hexamer stabilization (ΔTm = 0.5 °C for both), whereas a much stronger CA hexamer stabilizing effect was observed with compound **10** which bears a benzyl moiety at R^1^ (ΔTm = 2.5 °C). More importantly, compound **10** inhibited HIV-1 with a potency (EC_50_ = 1.6 μM) only 2.5-fold less than **PF74** (EC_50_ = 0.61 μM). In addition, compound **10** (CC_50_ > 100 μM) was less cytotoxic than **PF74** (CC_50_ = 76 μM).

### 3.2. SAR of Hit 11 (R^1^, R^3^, and R^4^)

Hit **11** was designed from hit **2** and **PF74** via molecular hybridization. As a result, the skeleton of **11** is very similar to that of **PF74**, except that the indole moiety was replaced with a bioisosteric quinazoline-2, 4-dione moiety. A general SAR observation was that when R^1^ was a bulky alkyl group, compounds (**14–21**) did not show significant antiviral activity at 20 μM or cytotoxicity at 100 μM (Table 2), though most analogs weakly stabilized CA hexamer (ΔTm = 0.5–0.7 °C). However, when R^1^ was a benzyl group to mimic **PF74**, the activity profiles changed substantially (analogs **11**–**13**) and demonstrated a strong dependence on R^3^. When R^3^ was a methyl group, analog **11** (ΔTm = 2.7 °C, EC_50_ = 6.9 μM) and analog **13** (ΔTm = 2.4 °C, EC_50_ = 8.0 μM) both moderately stabilized CA hexamer and inhibited HIV-1, without observed cytotoxicity (CC_50_ >100 μM). By contrast, when R^3^ was a hydrogen, the resulting analog (**12**) did not show activity in either assay. Overall, SARs around R^1^ and R^3^ within this series strongly indicate that both the benzyl group at R^1^ and the methyl group at R^3^ are important for potency.

### 3.3. SAR of Hit 22 (R^5^, R^6^, and R^7^)

Compound **22** is structurally quite different from **PF74** and the other two hits (**2** and **11**), though it satisfied multiple pharmacophore points (Figure 2A), and hence, could be similar to **PF74** in 3D shape. In addition, the 1,2,4-trazole is a well-known bioisostere [55] of carboxamide, which renders **22** functionally similar to **PF74**. The SARs of this series were centered around the two aromatic rings (R^5^ and R^7^). The most prominent observation was that analogs within this series did not significantly inhibit HIV-1 at 20 μM, and did not show cytotoxicity at 100 μM (Table 3). However, with the exception of **23**, **32**, and **33**, all analogs demonstrated an impact on CA hexamer stability, which essentially validates our hit generation approach as the shape similarity search is target-based. Very interestingly, a weak CA hexamer destabilizing effect, rather than stabilizing effect, was observed with hit **22** (ΔTm = −0.5 °C). A similar destabilizing effect was also observed with the only other analog featuring a pyridine ring at R^5^ (compound **31**), though the effect was significantly more prominent (ΔTm = −1.2 °C). All other analogs feature either a phenyl or a biphenyl ring at R^5^, and in stark contrast, produced CA hexamer stabilizing effect (ΔTm = 0.5–0.9 °C). This weak stabilizing effect was not impacted by functional group substitution at either R^5^ or R^7^. Finally, N-methyl substitution of analog **23** (ΔTm = 0 °C) yielded a compound **39** stabilizing CA hexamer (ΔTm = 1.2 °C). These observations strongly indicate that (1) pyridine at R^5^ destabilizes the CA hexamer whereas a phenyl or biphenyl moiety at R^5^ stabilizes the CA hexamer; (2) a methyl group at the para position of the phenyl ring at R^7^ reinforces the destabilizing effect (**31** vs. **22**) but does not impact the stabilizing effect (**28** vs. **24**, **30** vs. **36**); and (3) a methyl group at R^6^ confers a CA hexamer stabilizing effect (**39** vs. **23**).

### 3.4. Metabolic Stability

To characterize the drug-like property of **10**, our most potent antiviral compound from the current work, we conducted metabolic stability assays in both HLMs and mouse liver microsomes (MLMs). Metabolic stability is a major absorption, distribution, metabolism and excretion (ADME) property that profoundly impacts drug bioavailability [56]. Peptidomimetics are particularly susceptible to phase I metabolism, presumably because they are good substrates [57] for liver metabolizing enzyme subfamily cytochrome P450 3A (CYP3A), which is responsible for the metabolism of at least 50% of all current drugs [58]. It is known that **PF74** is a severely flawed antiviral lead due to its prohibitively low metabolic stability [34]. This was confirmed in our metabolic stability assays, where the half-life (t_1/2_) of **PF74** is less than 1 min in both HLMs and MLMs (Table 4). By contrast, our compound **10** was decisively more stable, particularly in HLMs where its half-life (t_1/2_ = 31 min) was 44-fold longer than that of **PF74** (t_1/2_ = 0.7 min). When tested in combination with a CYP3A inhibitor Cobicistat (Cobi) [59], **10** still exhibited significantly longer half-life than that of **PF74**. A t_1/2_ >30 min in HLM generally indicates good in vivo metabolic stability and oral bioavailability. Collectively, these observations support our compound **10** as a viable antiviral hit, and corroborate the hypothesis that the poor metabolic stability of **PF74** is due to CYP3A-mediated phase I metabolism.

### 3.5. Molecular Modeling

**Figure 3:**
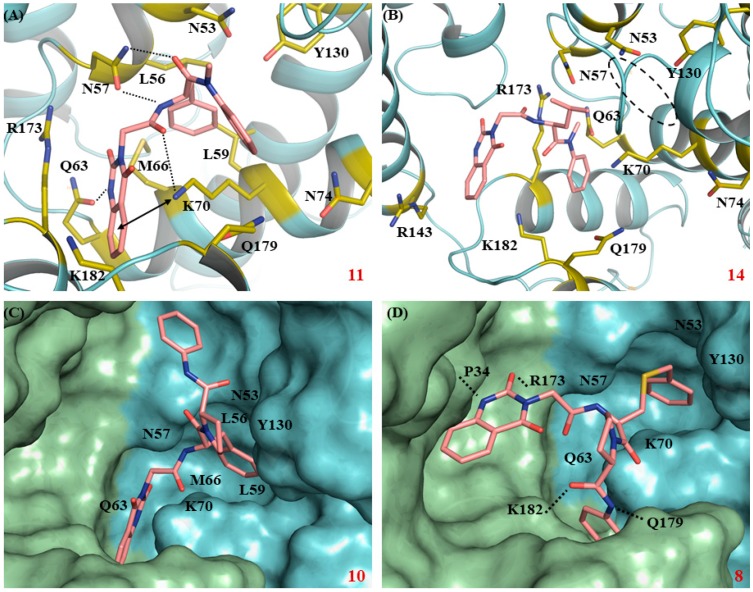
Docking poses of key ligands based on native HIV-1 capsid protein bound to **PF74** (PDB code: 4XFZ [25]). (**A**) Predicted binding mode of **11**. Glide score (kcal/mol): −6.3. (**B**) Predicted binding mode of **14** away from the preferred binding site (circled). Glide scores (kcal/mol): −4.2. (**C**) Predicted binding and fitting of *L*-proline analog **10** within the preferred **PF74** binding cavity. Glide score (kcal/mol): −6.3. (**D**) Predicted binding and fitting of *L*-cysteine derivative analog **8** away from the preferred **PF74** binding site. Glide score (kcal/mol): −5.2. Ligands numbers **8**, **10**, **11**, and **14** are in pink. In Figure **A** and **D**, H-bond and cation-π interactions are depicted as black dotted lines and double headed arrow, respectively. In (**A**) and (**B**), the capsid protein chain is colored cyan and key residues around binding site are colored olive. In (**C**) and (**D**), CA_NTD_ around helices H3 and H4 in chain A are colored cyan, and CA_CTD_ around helices H8 and H9 in the adjacent chain B are colored green. The nitrogen, oxygen, and sulfur atoms are colored blue, red, and yellow, respectively.

To understand some of the aforementioned SAR, we performed molecular modeling with selected compounds based on the co-crystal structure of native HIV-1 capsid protein bound to **PF74** (PDB code: 4XFZ [25]). Compound **11** (Figure 3A) bearing a quinazoline-2,4(1*H*,3*H*)-dione core in place of the indole ring of **PF74** interacted with the same key residues in the CA hexamer as **PF74**. Observed key interactions included (i) hydrogen-bonding between N57 and the NH and carbonyl groups of the phenylalanine fragment on compound **11**, between K70 and the carbonyl next to the phenylalanine fragment of **11**, and between Q63 and the free NH of the quinazoline-2,4(1*H*,3*H*)-dione core of **11**; (ii) cation-π interactions between protonated K70 and quinazoline-2,4(1*H*,3*H*)-dione aromatic ring. However, despite sharing the same binding site and similar key interactions, compound **11** was significantly less potent than **PF74**. In the meantime, a complete loss in potency was observed for leucine derived analog **14**, which could be attributed to its shifting away from the binding site (circled), possibly in a binding mode shown in Figure 3B, and hence the loss of key interactions. This observation signified the importance of the phenylalanine core of these compounds for potency. In particular, the benzyl group of the phenylalanine core could play an important role in keeping a molecule inside the **PF74**-bound cavity through hydrophobic interactions [25] with surrounding residues L56, M66, and L69, as evident from the observed binding mode for compound **11** (Figure 3A). This was consistent with the significant increase in the potency of compound **10** as well, which bears a benzyl group and an additional *L*-proline core. Like the **PF74** backbone, the benzyl group of the phenylalanine fragment forces compound **10** to nestle in the cavity in such a way that the aniline ring of the *L*-proline core is extended to the adjacent CA_NTD_ domain, resulting in an additional hydrogen-bonding between the carbonyl of the *L*-proline and NH_2_ of N53, along with the typical H-bonding with N57, Q63, and K70 (Figure 3C). Additionally, pyrrolidine core of *L*-proline is oriented in such a way that it has maximum interaction with A105, T107, and Y130. Expectedly, introduction of an *L*-cysteine derived core having an elongated backbone in place of phenylalanine in compound **8** forces the entire molecule out of the cavity, diminishing it potency (Figure 3D). Compound **8** was found to be closer to the adjacent CA_CTD_ and CA_NTD_ domains, interacting with P34, R173, Q179, and K182 through H-bonding.

## 4. Discussion

Despite the approval of many HIV-1 antiviral regimens [60], a curative therapy remains elusive and HIV-1 continues to pose a global healthcare challenge. There is a need to develop new classes of HIV-1 drugs with distinct mechanisms of action to manage HIV-1 strains resistant to current drugs. The multifunctional HIV-1 CA represents an attractive target for novel antiviral discovery. **PF74** is a CA-targeting small molecule which binds to a unique pocket between a viral CA_NTD_ and the adjacent CA_CTD_, and competes against a few host factors important for viral replication. The antiviral profile of **PF74** and its mode of CA binding are well characterized. However, **PF74** is not a viable antiviral lead as it suffers from extremely low metabolic stability. Aiming to identify mechanistically similar yet structurally distinct small molecules with improved metabolic stability, we performed a pharmacophore-based shape similarity search based on **PF74**. Subsequently, we conducted analog synthesis and SAR for two hits (**2** and **22**) generated from the shape similarity search, as well as a third hit (**11**) designed via molecular hybridization. Overall, most of analogs exhibited weak yet discernible impacts on the stability of CA hexamer, which largely validates our hit generation approach. Three of the analogs (**10**, **11**, and **13**) showed moderate CA hexamer stabilizing effects and significant anti-HIV-1 activities. Particularly, compound **10** inhibited HIV-1 with an EC_50_ of 1.6 μM, which is only 2.5-fold less potent that **PF74**. More importantly, compound **10** demonstrated drastically improved metabolic stability over **PF74** in HLMs (t_1/2_ = 31 min for **10** vs. 0.7 min for **PF74**). Molecular modeling indicates that our compound **10** binds comfortably in the **PF74** binding pocket. Collectively, our data support **10** as a potent and metabolically stable HIV-1 CA-targeting antiviral lead.

## Figures and Tables

**Table 1:**
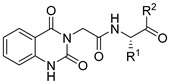
Anti-HIV-1 activity, cytotoxicity, and CA hexamer stability profiles of **2**–**10** (R^1^, R^2^).

Compd	R^1^	R^2^	EC_50_ (μM) ^[a]^	CC_50_ (μM) ^[b]^	TSA ΔTm (°C) ^[c]^
**1 (PF74)**	--	--	0.61 ± 0.2	76 ± 9	7.4
**2**	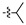	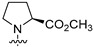	>20	>100	0.5
**3**	H	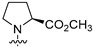	>20	>100	0
**4**	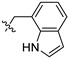	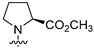	>20	<100	0.5
**5**	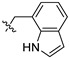	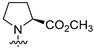	>20	>100	0.8
**6**	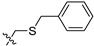	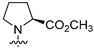	>20	>100	0
**7**	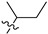	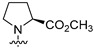	>20	>100	0
**8**	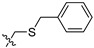	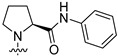	>20	44 ± 5	0.5
**9**	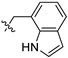	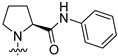	>20	>100	0.5
**10**	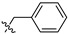	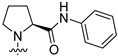	1.6 ± 0.1	>100	2.5

^a^ Concentration of compound inhibiting HIV-1 replication by 50%, expressed as the mean ± standard deviation from at least two independent experiments. ^b^ Concentration of compound causing 50% cell death, expressed as the mean ± standard deviation from at least two independent experiments. ^c^ TSA: thermal shift assay. ΔTm: change of CA crosslinked hexamer melting point in the presence of compound minus DMSO control.

**Table 2:** Anti-HIV-1 activity, cytotoxicity, and CA hexamer stability profiles of **11–21** (R^1^, R^3^, and R^4^).

Compd	R^1^	R^3^	R^4^	EC_50_ (μM) ^[a]^	CC_50_ (μM) ^[b]^	TSA ΔTm (°C) ^[c]^
**1 (PF74)**	--	--	--	0.61 ± 0.2	76 ± 9	7.4
**11**	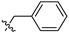	Me	H	6.9 ± 0.8	>100	2.7
**12**	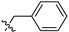	H	H	>20	<100	0
**13**	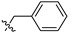	Me	F	8.0 ± 1.3	>100	2.4
**14**	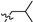	Me	H	>20	>100	0
**15**	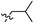	H	H	>20	>100	0.7
**16**	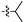	Me	Me	>20	>100	0
**17**	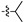	H	H	>20	>100	0
**18**	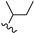	Me	H	>20	>100	0.5
**19**	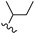	H	H	>20	>100	0.6
**20**	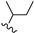	Me	Cl	>20	>100	0.5
**21**	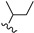	H	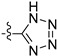	>20	>100	0.6

^a^ Concentration of compound inhibiting HIV-1 replication by 50%, expressed as the mean ± standard deviation from at least two independent experiments. ^b^ Concentration of compound causing 50% cell death, expressed as the mean ± standard deviation from at least two independent experiments. ^c^ TSA: thermal shift assay. ΔTm: change of CA crosslinked hexamer melting point in the presence of compound minus DMSO control.

**Table 3:**
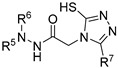
Anti-HIV-1 activity, cytotoxicity, and CA hexamer stability profiles of **22–39** (R^5^, R^6^, and R^7^).

Compd	R^5^	R^6^	R^7^	EC_50_ (μM) ^[a]^	CC_50_ (μM) ^[b]^	TSA ΔTm (°C) ^[c]^
**22**	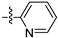	H	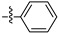	>20	>100	−0.5
**23**	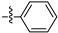	H	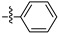	>20	>100	0
**24**	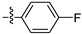	H	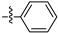	>20	>100	0.7
**25**	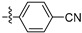	H	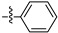	>20	>100	0.6
**26**	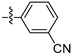	H	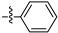	>20	>100	0.7
**27**	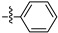	H	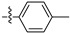	>20	~100	0.7
**28**	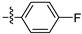	H	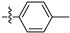	>20	>100	0.7
**29**	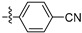	H	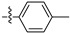	>20	>100	0
**30**	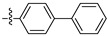	H	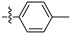	>20	>100	0.8
**31**	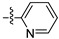	H	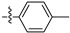	>20	>100	−1.2
**32**	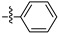	H	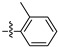	>20	>100	0
**33**	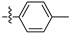	H	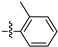	>20	>100	0
**34**	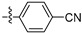	H	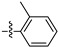	>20	>100	0.6
**35**	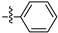	H	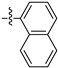	>20	>100	0.5
**36**	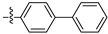	H	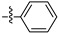	>20	>100	0.8
**37**	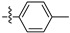	H	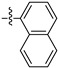	>20	>100	1.0
**38**	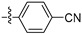	H	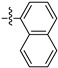	>20	>100	0.9
**39**	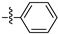	Me	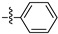	>20	>50	1.2

^a^ Concentration of compound inhibiting HIV-1 replication by 50%, expressed as the mean ± standard deviation from at least two independent experiments. ^b^ Concentration of compound causing 50% cell death, expressed as the mean ± standard deviation from at least two independent experiments. ^c^ TSA: thermal shift assay. ΔTm: change of CA crosslinked hexamer melting point in the presence of compound minus DMSO control.

**Table 4:** Phase I metabolic stability in liver microsomes t_1/2_ (min).

Compound	HLM ^a^	HLM ^a^ (+Cobi ^c^)	MLM ^b^	MLM ^b^ (+Cobi ^c^)
**PF74**	0.7	91	0.6	34
**10**	31	>120	2.9	85
**Verapamil**	15	--	4.2	--

^a^ HLM: human liver microsome; ^b^ MLM: mouse liver microsome; ^c^ Microsomal stability measured in the presence of CYP3A inhibitor Cobi.

## Supplementary Materials

The following are available online at https://www.mdpi.com/1999-4915/12/4/452/s1, Scheme S1: Synthesis of intermediates **b**–**d**, and compounds **2**–**21**, Scheme S2: Synthesis of intermediates **f**–**g** and compounds **22**–**39**.
