# Supplementary material for: Toward Structurally Novel and Metabolically Stable HIV-1 Capsid-Targeting Small Molecules
Source: Viruses. 2020 Apr 16;12(4):452. doi: 10.3390/v12040452 (PMC7232165; doi:10.3390/v12040452)
Supplement: Supplementary file 1 [file viruses-12-00452-s001.pdf]

Supporting Information

Toward Structurally Novel and Metabolically Stable HIV-1 Capsid-Targeting Small Molecules

Sanjeev Kumar V. Vernekar a, #, Rajkumar Lalji Sahani a, #, Mary Casey b, Jayakanth Kankanala a, Lei Wang a, Karen A. Kirby c, Haijuan Du c, Huanchun Zhang c, Philip R. Tedbury c, Jiashu Xie a, Stefan G. Sarafianos b,c, and Zhengqiang Wang a*

a Center for Drug Design, College of Pharmacy, University of Minnesota, Minneapolis, MN 55455, USA

b Department of Molecular Microbiology and Immunology, University of Missouri School of Medicine, Christopher S. Bond Life Sciences Center, Columbia, MO 65211, USA

*c Laboratory of Biochemical Pharmacology, Department of Pediatrics, Emory University School of Medicine, Atlanta, GA 30322, USA**

** Correspondence: wangx472@umn.edu; Tel.: +1-612-626-7025*

# These authors contributed equally to this work.

Table of Contents

1. Scheme 1: Synthesis of intermediates **b**, **c**, and **d** and spectral data S2
2. Synthesis of compounds **2-4** and spectral data S6
3. Synthesis of compounds **5-7** and spectral data S7
4. Synthesis of compounds **8-10** and spectral data S9
5. Synthesis of compounds **11-21** and spectral data S11
6. Scheme 2: Synthesis of intermediates **f** and **g** and spectral data S15
7. Synthesis of compounds **22-39** and spectral data S18
8. NMR spectra of compounds **2-39** S25

Scheme 1: Synthesis of intermediates b-d, and compounds 2-21.

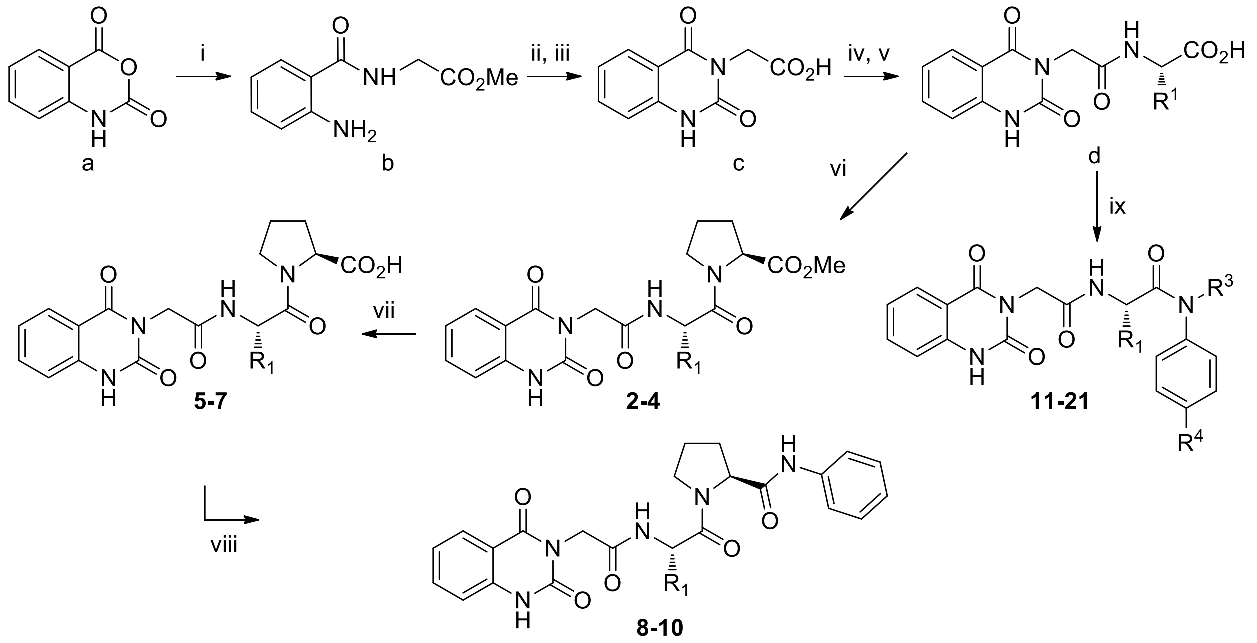

Reagents and conditions: (i) glycine methyl ester hydrochloride, Et_3N, DMF, rt, 12 h; (ii) ethyl chloroformate, pyridine, 0 °C - rt, 12 h; (iii) NaOH, $MeOH/H_2O$, 60 °C, 12 h; (iv) *L*-amino acid methyl ester hydrochloride, HATU, DIPEA, DMF, rt, 12 h; (v) LiOH, $MeOH/H_2O$, rt, 12 h; (vi) *L*-proline methyl ester hydrochloride, PyAOP, DIPEA, DMF, rt, 12 h; (vii) LiOH, $MeOH/H_2O$, rt, 12 h/60 °C, 3 h; (viii) amine, PyAOP, DIPEA, DMF, rt, 12 h; (ix) amine, HATU, DIPEA, DMF, rt, 12 h.

***Synthesis of intermediate b*:** To a solution of Isatoic anhydride **a** (1.0 gm, 1 equiv.) in DMF (10 mL) glycine methyl ester hydrochloride (1.1 equiv.), and Et_3N (1.2 equiv.) were added and the mixture was stirred at room temperature for 12 hours. Upon completion, reaction mixture was diluted with H_2O (30 mL) and extracted with EtOAc (3x30 mL). Combined organic phases were washed with water, brine, dried over anhydrous Na_2SO_4, filtered and concentrated. After drying completely, the crude product **b** (1.1 gm, 85%) was used in the next step without further purification.

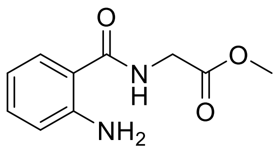

^1H NMR (600 MHz, DMSO-d_6) δ 8.64 (t, *J* = 5.8 Hz, 1H), 7.52 (dd, *J* = 7.9, 1.5 Hz, 1H), 7.15 (ddd, *J* = 8.4, 7.0, 1.4 Hz, 1H), 6.70 (dd, *J* = 8.2, 1.2 Hz, 1H), 6.52 (ddd, *J* = 8.1, 7.0, 1.2 Hz, 1H), 6.45 (s, 2H), 3.94 (d, J = 5.8 Hz, 2H), 3.65 (s, 3H).

Synthesis of intermediate c: To a pyridine (10 mL) solution of compound **b** (1.1 gm, 1.0 equiv.) cooled at 0 °C was added ethyl chloroformate (1.2 equiv.) dropwise, the reaction mixture was slowly warmed to room temperature and stirred for 12 hours. Upon completion, solvent was evaporated, H_2O (30 mL) and 0.1N HCl were added to precipitate out pale yellow solid. The crude product was filtered over Buchner funnel, washed with water and dried to get ethyl amino formate derivative (1.25 gm, 95%) that was used in next step without further purification.

To the solution of ethyl amino formate derivative (1.25 gm, 1.0 equiv.) in methanol (10 mL) was added 1N NaOH (5 mL) and the reaction mixture was heated at 60 ℃ for 12 hours. Upon completion, ethanol was evaporated, and the reaction mixture was acidified till pH = 3 using 1N HCl to precipitate out white solid. Filtration of the crude product over Buchner funnel, washing with water, and drying gave compound **c** (0.79 gm, 80%) as white solid that was used in the next step without further purification.

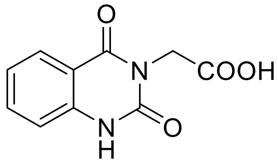

^1H NMR (600 MHz, DMSO-d_6) δ 11.58 (s, 1H), 7.94 (dd, *J* = 8.0, 1.5 Hz, 1H), 7.70 (ddd, *J* = 8.5, 7.2, 1.5 Hz, 1H), 7.27 – 7.20 (m, 2H), 4.55 (s, 2H).

Synthesis of intermediate d: To a solution of acid **c** (1.0 gm, 1 equiv.) in DMF (10 mL), HATU (1.2 equiv.) and DIPEA (2.0 equiv.) were added and the mixture was stirred at room temperature for 20 min. Followed by addition of desired *L*-amino acid methyl ester hydrochloride (1.1 equiv.) and the mixture was further stirred at room overnight for 12 hours. Upon completion, H_2O (50 mL) was added and the reaction mixture was extracted with EtOAc (3x50 mL). The combined organic phases were washed with water, brine, dried over anhydrous Na_2SO_4, filtered and concentrated. The crude product was purified by Combi-flash on silica gel using 50-100% EtOAc/hexane.

To a solution of the desired ester products obtained in the previous step in methanol (10 mL) was added LiOH (3.0 equiv.) dissolved in H_2O (10 mL) and the mixture was stirred at room temperature for 12 hours. Upon completion, ethanol was evaporated and acidified to pH = 3 using 1N HCl. Filtration of the crude product, washing, and drying gave the respective acids **d**.

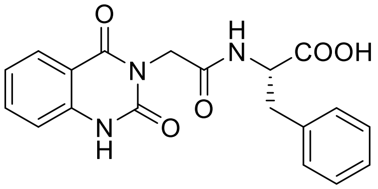

^{1}H NMR (600 MHz, DMSO-d_6) δ 12.77 (s, 1H), 11.48 (s, 1H), 8.51 (d, J = 7.9 Hz, 1H), 7.92 (d, J = 7.9 Hz, 1H), 7.67 (t, J = 7.8 Hz, 1H), 7.28 (t, J = 7.5 Hz, 2H), 7.22 – 7.21 (m, 5H), 4.56 – 4.45 (m, 2H), 4.41 (q, J = 7.3 Hz, 1H), 3.03 (dd, J = 13.9, 5.3 Hz, 1H), 2.91 (dd, J = 13.8, 8.4 Hz, 1H).

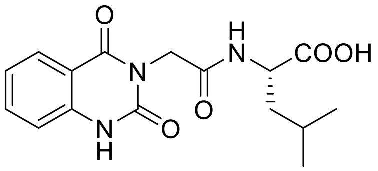

^{1}H NMR (600 MHz, DMSO-d_6) δ 12.60 (s, 1H), 11.49 (s, 1H), 8.41 (d, J = 8.1 Hz, 1H), 7.92 (dd, J = 8.1, 1.6 Hz, 1H), 7.70 – 7.65 (m, 1H), 7.22 (dd, J = 8.0, 6.0 Hz, 2H), 4.58 – 4.47 (m, 2H), 4.25

(td, *J* = 8.6, 5.8 Hz, 1H), 1.65 (dq, *J* = 13.2, 6.7 Hz, 1H), 1.56 – 1.47 (m, 2H), 0.89 (d, *J* = 6.6 Hz, 3H), 0.84 (d, J = 6.5 Hz, 3H).

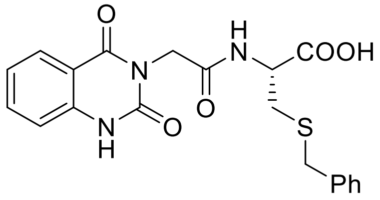

^{1}H NMR (600 MHz, DMSO-d_6) δ 12.94 (s, 1H), 11.49 (s, 1H), 8.64 (d, *J* = 8.0 Hz, 1H), 7.93 (d, *J* = 7.8 Hz, 1H), 7.68 (t, *J* = 7.7 Hz, 1H), 7.31 (d, *J* = 6.0 Hz, 4H), 7.27 – 7.19 (m, 3H), 4.59 (s, 2H), 4.48 (td, *J* = 7.7, 5.1 Hz, 1H), 3.78 (s, 2H), 2.79 (dd, *J* = 13.8, 5.3 Hz, 1H), 2.69 (dd, *J* = 13.7, 7.5 Hz, 1H).

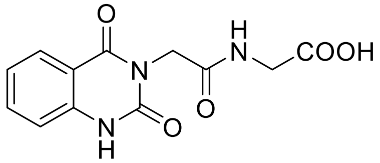

^{1}H NMR (600 MHz, DMSO-d_6) δ 12.60 – 12.57 (m, 1H), 11.48 (s, 1H), 8.47 (t, *J* = 6.0 Hz, 1H), 7.92 (d, *J* = 7.9 Hz, 1H), 7.68 (t, *J* = 8.0 Hz, 1H), 7.25 – 7.18 (m, 2H), 4.54 (s, 2H), 3.78 (d, *J* = 5.8 Hz, 2H).

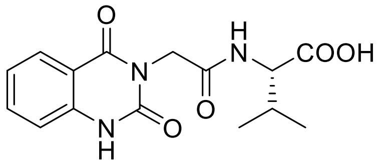

^{1}H NMR (600 MHz, DMSO-d_6) δ 12.65 (s, 1H), 11.46 (s, 1H), 8.37 (d, *J* = 8.7 Hz, 1H), 7.92 (d, *J* = 7.8 Hz, 1H), 7.68 (t, *J* = 7.8 Hz, 1H), 7.25 – 7.18 (m, 2H), 4.63 – 4.53 (m, 2H), 4.17 (dd, *J* = 8.7, 6.0 Hz, 1H), 2.05 (h, *J* = 6.7 Hz, 1H), 0.89 (dd, J = 7.0, 3.7 Hz, 6H).

Synthesis of compounds 2-4: To a solution of acid **d** (1 equiv.) in DMF (5 mL), PyAOP (1.2 equiv.) and DIPEA (2.0 equiv.) were added and the mixture was stirred at room temperature for 20 min. Followed by addition of desired *L*-proline methyl ester hydrochloride (1.2 equiv.) and the mixture was further stirred at room overnight for 12 hours. Upon completion, H_2O (20 mL) was added and the reaction mixture was extracted with EtOAc (3x20 mL). The combined organic phases were washed with water, brine, dried over anhydrous Na_2SO_4, filtered and concentrated. The crude product was purified by Combi-flash on silica gel using 50-100% EtOAc/hexane.

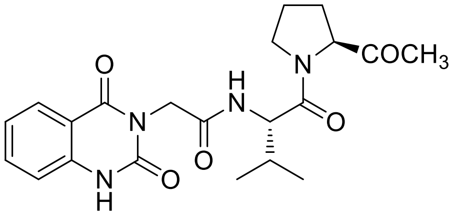

2, ^{1}H NMR (600 MHz, DMSO-*d*$_6$) δ 11.46 (d, *J* = 7.7 Hz, 1H), 8.21 (d, *J* = 9.0 Hz, 1H), 7.92 (ddd, *J* = 8.0, 3.8, 1.5 Hz, 1H), 7.68 (ddt, *J* = 8.5, 7.2, 1.4 Hz, 1H), 7.41 (dt, *J* = 15.2, 7.8 Hz, 2H), 7.33 (q, *J* = 7.5 Hz, 3H), 7.26 – 7.18 (m, 2H), 4.64 – 4.49 (m, 3H), 3.18 (s, 1H), 1.53 (dt, *J* = 13.1, 6.7 Hz, 1H), 1.02 (dt, *J* = 14.4, 7.2 Hz, 1H), 0.93 – 0.79 (m, 6H). ^{13}C NMR (100 MHz, DMSO-*d*$_6$) δ 173.2, 169.8, 166.6, 161.8, 150.0, 139.5, 135.1, 127.4, 122.6, 115.2, 113.7, 58.5, 55.7, 46.9, 42.2, 30.4, 28.7, 24.5, 18.8, 18.5; HRMS (ESI) m/z calcd for $C_{21}H_{26}N_4O_6$ $[M + H]^+$ 431.1925, found 431.1923.

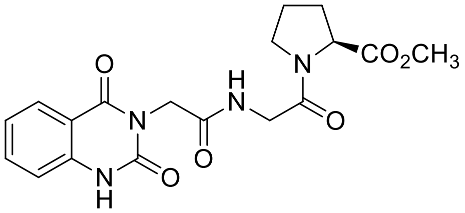

3, ^{1}H NMR (400 MHz, DMSO-*d*$_6$) δ 11.43 (s, 1H), 8.31 (s, 1H), 7.93 (d, *J* = 7.7 Hz, 1H), 7.68 (t, *J* = 7.5 Hz, 1H), 7.23 (t, *J* = 7.7 Hz, 2H), 4.58 – 4.52 (m, 2H), 4.33 (dd, *J* = 8.7, 4.1 Hz, 1H), 4.03

(dd, J = 17.2, 5.4 Hz, 1H), 3.89 (dd, J = 17.1, 5.1 Hz, 1H), 3.63 (s, 3H), 3.59 – 3.41 (m, 2H), 2.21 – 2.12 (m, 1H), 2.00 – 1.79 (m, 3H). ^{13}C NMR (100 MHz, DMSO-d_6) δ 172.8, 167.4, 167.3, 162.2, 150.5, 139.9, 135.6, 127.9, 123.0, 115.7, 114.2, 59.0, 52.2, 46.1, 42.8, 41.6, 29.1, 24.9; HRMS (ESI) m/z calcd for $C_{18}H_{20}N_4O_6$ [M + H]$^+$ 389.1456, found 389.1460.

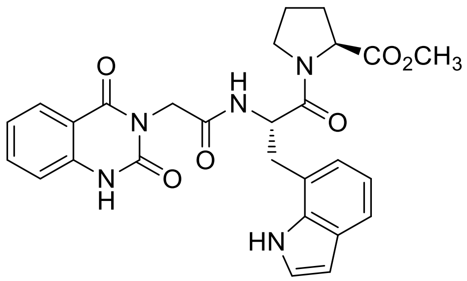

4, ^{1}H NMR (400 MHz, DMSO-d_6) δ 11.47 (s, 1H), 10.91 (s, 1H), 8.68 (d, J = 7.9 Hz, 1H), 7.92 (d, J = 7.0 Hz, 1H), 7.73 – 7.63 (m, 1H), 7.58 (d, J = 7.8 Hz, 1H), 7.35 (d, J = 8.0 Hz, 1H), 7.29 – 7.12 (m, 3H), 7.08 (t, J = 7.5 Hz, 1H), 7.00 (t, J = 7.0 Hz, 1H), 4.84 – 4.75 (m, 1H), 4.58 – 4.44 (m, 2H), 4.36 – 4.31 (m, 1H), 3.61– 3.56 (m, 4H), 3.16 – 3.00 (m, 2H), 2.99 – 2.82 (m, 1H), 2.15 – 2.10 (m, 1H), 1.84 – 1.71 (m, 3H). ^{13}C NMR (100 MHz, DMSO-d_6) δ 172.7, 170.3, 166.9, 162.2, 150.5, 139.9, 136.5, 135.6, 127.9, 127.7, 124.3, 123.1, 121.4, 118.9, 118.4, 115.6, 114.2, 111.8, 109.9, 59.0, 52.2, 51.8, 46.9, 42.7, 29.1, 27.7, 25.0; HRMS (ESI) m/z calcd for $C_{27}H_{27}N_5O_6$ [M + H]$^+$ 518.2034, found 518.2039.

Synthesis of compounds 5-7: *L*-proline esters **2-4** obtained in the previous step were subjected to hydrolysis described in the second part of synthesis of **d**. Except, in some cases a further heating at 60 °C for 3 hours was desired for a complete hydrolysis after initial stirring of the reaction mixture at room temperature for 12 hours.

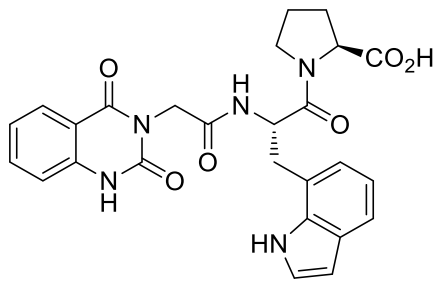

5, ^{1}H NMR (400 MHz, DMSO-d_6) δ 12.56 (s, 1H), 11.46 (s, 1H), 10.88 (s, 1H), 8.65 (d, J = 7.9 Hz, 1H), 7.92 (dd, J = 7.9, 0.9 Hz, 1H), 7.73 – 7.62 (m, 1H), 7.57 (d, J = 7.8 Hz, 1H), 7.38 – 7.31 (m, 1H), 7.26 – 7.14 (m, 3H), 7.07 (t, J = 7.5 Hz, 1H), 6.71 – 6.95 (m, 1H), 4.75 – 4.68 (m, 1H), 4.66 – 4.48 (m, 2H), 4.32 – 4.23 (m, 1H), 3.60 – 3.55 (m, 1H), 3.42 – 3.39 (m, 2H), 3.19 – 2.88 (m, 2H), 2.16 – 2.11 (m, 1H), 1.97 – 1.67 (m, 2H). ^{13}C NMR (100 MHz, DMSO-d_6) δ 173.7, 170.2, 166.9, 162.2, 150.5, 139.9, 136.5, 135.6, 127.9, 127.7, 124.4, 123.0, 121.3, 118.9, 118.4, 115.6, 114.1, 111.8, 110.1, 59.1, 51.9, 46.9, 42.7, 29.1, 27.6, 24.9; HRMS (ESI) m/z calcd for $C_{26}H_{25}N_5O_6$ $[M + H]^+$ 504.1878, found 504.1878.

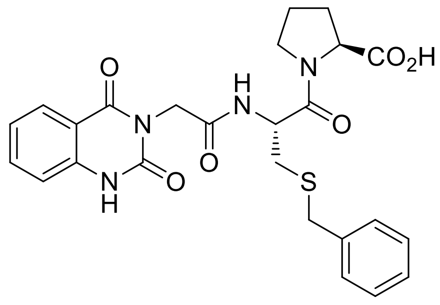

6, ^{1}H NMR (400 MHz, DMSO-d_6) δ 12.54 (s, 1H), 11.49 (s, 1H), 8.67– 8.62 (m, 1H), 7.94 (d, J = 7.7 Hz, 1H), 7.69 (t, J = 7.7 Hz, 1H), 7.43 – 7.27 (m, 4H), 7.28 – 7.16 (m, 3H), 4.78 – 4.64 (m, 1H), 4.65 – 4.47 (m, 2H), 4.26 – 4.22 (m, 1H), 3.84 – 3.75 (m, 2H), 3.64 – 3.48 (m, 1H), 3.48 – 3.35 (m, 2H), 2.77 – 2.71 (m, 1H), 2.15 – 2.11 (m, 1H), 1.92 – 1.83 (m, 3H). ^{13}C NMR (100 MHz, DMSO-d_6) δ 173.5, 168.9, 167.1, 162.3, 150.5, 139.9, 138.9, 135.6, 129.5, 129.4, 128.8, 127.9, 127.3, 123.1, 115.7, 114.2, 59.2, 50.9, 46.9, 42.7, 40.6, 40.4, 40.2, 40.0, 39.8, 39.6, 39.4, 36.0,

35.9, 32.9, 29.1, 24.8; HRMS (ESI) m/z calcd for $C_{25}H_{26}N_4O_6S$ $[M + H]^+$ 511.1646, found 511.1648.

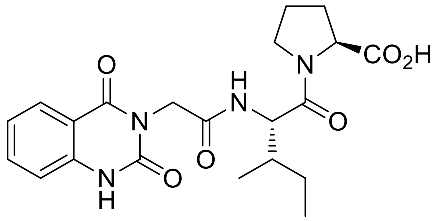

7, ^{1}H NMR (400 MHz, DMSO-d_6) δ 12.44 (s, 1H), 11.47 (s, 1H), 8.48 (d, *J* = 8.5 Hz, 1H), 7.92 (d, *J* = 7.7 Hz, 1H), 7.68 (t, *J* = 7.5 Hz, 1H), 7.27 – 7.14 (m, 2H), 4.69 – 4.46 (m, 2H), 4.38 (t, *J* = 8.8 Hz, 1H), 4.31 – 4.12 (m, 1H), 3.75 – 3.71 (m, 1H), 3.64 – 3.47 (m, 1H), 2.22 – 2.08 (m, 1H), 1.96 – 1.68 (m, 4H), 1.64 – 1.45 (m, 1H), 1.16 – 1.04 (m, 1H), 1.02 – 0.69 (m, 6H). ^{13}C NMR (100 MHz, DMSO-d_6) δ 173.7, 170.4, 166.9, 162.2, 150.5, 139.9, 135.6, 127.9, 123.0, 115.6, 114.2, 59.0, 55.4, 54.9, 47.3, 42.6, 36.9, 29.2, 24.9, 15.2, 11.2; HRMS (ESI) m/z calcd for $C_{21}H_{26}N_4O_6$ $[M + H]^+$ 413.1925, found 413.1920.

Synthesis of compounds 8-10: A similar synthetic procedure described to synthesize compounds **2-4** was employed to synthesize products **8-10** using desired amines in place of *L*-proline methyl ester hydrochloride.

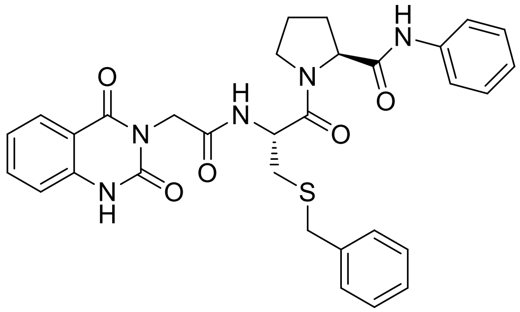

8, ^{1}H NMR (600 MHz, DMSO-d_6) δ 11.48 (s, 1H), 9.86 (s, 1H), 8.66 (d, *J* = 8.2 Hz, 1H), 7.92 (d, *J* = 8.0 Hz, 1H), 7.67 (t, *J* = 7.7 Hz, 1H), 7.54 (d, *J* = 8.1 Hz, 2H), 7.33 (d, *J* = 7.6 Hz, 2H), 7.31 –

7.17 (m, 7H), 7.01 (t, *J* = 7.0 Hz, 1H), 4.75 – 4.70 (m, 1H), 4.59 – 4.49 (m, 2H), 4.42 – 4.39 (m, 1H), 3.82 – 3.75 (m, 2H), 3.63 – 3.57 (m, 1H), 3.50 – 3.45 (m, 1H), 2.79 (dd, *J* = 13.8, 5.9 Hz, 1H), 2.60 – 2.51 (m, 1H), 2.12 – 2.10 (m, 1H), 1.98 – 1.91 (m, 1H), 1.92 – 1.83 (m, 2H); HRMS (ESI) m/z calcd for $C_{31}H_{31}N_5O_5S$ $[M + H]^+$ 586.2119, found 586.2121.

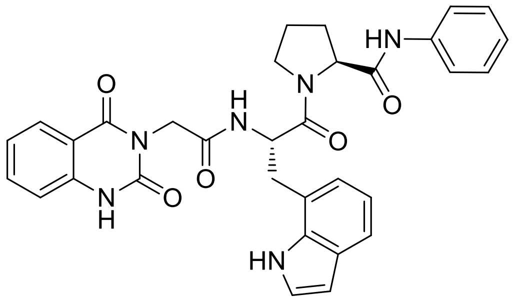

9, ^{1}H NMR (400 MHz, DMSO-*d*$_6$) δ 11.47 (s, 1H), 10.91 (s, 1H), 9.98 (s, 1H), 8.64 (d, *J* = 7.6 Hz, 1H), 7.92 (d, *J* = 7.7 Hz, 1H), 7.69 (t, *J* = 7.7 Hz, 1H), 7.60 – 7.54 (m, 3H), 7.35 – 7.18 (m, 6H), 7.09 – 6.97 (m, 3H), 4.79 – 4.72 (m, 1H), 4.56 – 4.43 (m, 2H), 3.63 – 3.60 (m, 1H), 3.41 – 3.39 (m, 2H), 3.17 – 3.12 (m, 1H), 2.95 (dd, *J* = 14.5, 7.7 Hz, 1H), 2.25 – 2.06 (m, 1H), 1.95 – 1.83 (m, 3H). ^{13}C NMR (100 MHz, DMSO-*d*$_6$) δ 170.8, 170.4, 167.0, 162.2, 150.5, 139.6, 139.0, 136.5, 135.6, 129.1, 127.9, 127.7, 124.4, 123.1, 121.3, 119.6, 118.9, 115.6, 114.1, 110.1, 60.8, 52.2, 47.3, 29.8, 27.5, 25.0; HRMS (ESI) m/z calcd for $C_{32}H_{30}N_6O_5$ $[M + H]^+$ 579.235, found 579.2349.

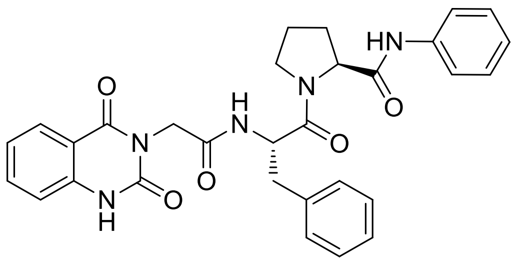

10, ^{1}H NMR (400 MHz, DMSO-*d*$_6$) δ 11.45 (s, 1H), 8.61 (d, *J* = 7.7 Hz, 1H), 7.91 (d, *J* = 7.8 Hz, 1H), 7.68 (t, *J* = 7.7 Hz, 1H), 7.24 – 7.20 (m, 6H), 7.04 – 6.98 (m, 2H), 6.94 – 6.89 (m, 4H), 4.54 – 4.52 (m, 1H), 4.46 – 4.33 (m, 2H), 3.79 – 3.75 (m, 3H), 3.12 – 3.09 (m, 3H), 2.90 – 2.83 (m,

2H), 2.68 (dd, *J* = 13.4, 8.7 Hz, 1H). ^{13}C NMR (100 MHz, DMSO-*d₆*) δ 171.4, 166.8, 162.1, 158.9, 150.4, 139.9, 137.9, 135.9, 135.6, 129.4, 129.1, 128.6, 127.9, 126.9, 123.0, 115.6, 115.0, 114.1, 55.8, 51.9, 42.5, 37.9, 37.8, 36.2, 31.2; HRMS (ESI) m/z calcd for $C_{30}H_{29}N_5O_5$ $[M + H]^+$ 540.2241, found 540.2243.

Synthesis of compounds 11-21: To a solution of acid **d** (1 equiv.) in DMF (3 mL) was added HATU (1.2 equiv.) DIPEA (2 equiv) and the mixture was stirred at room temperature for 20 min. Followed by addition of desired amine (1.2 equiv.) and the reaction was further stirred at room overnight for 12 hours. Upon completion, H_2O was added, and the reaction mixture was extracted with EA (3x20 mL). The organic phases were combined and washed with brine, dried over anhydrous Na_2SO_4, filtered and concentrated to yield the crude product. The product was purified by combi-flash on silica gel using 50% EtOAc/hexane.

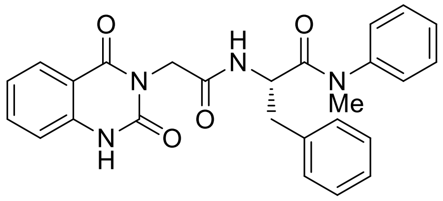

11, ^{1}H NMR (400 MHz, DMSO-d_6) δ 11.45 (s, 1H), 8.63 (d, *J* = 7.5 Hz, 1H), 7.91 (d, *J* = 7.7 Hz, 1H), 7.68 (t, *J* = 7.7 Hz, 1H), 7.41 – 7.35 (m, 1H), 7.30 – 7.03 (m, 9H), 6.84 – 6.81 (m, 2H), 4.56 – 4.44 (m, 3H), 3.14 (s, 3H), 2.93 – 2.78 (m, 1H), 2.75 – 2.58 (m, 1H). ^{13}C NMR (100 MHz, DMSO-d_6) δ 171.2, 166.9, 166.7, 162.1, 153.4, 150.4, 142.6, 137.8, 137.6, 135.6, 133.3, 129.9, 129.3, 128.6, 127.9, 126.1, 123.1, 116.0, 115.6, 112.1, 55.6, 42.4, 42.3, 37.6; HRMS (ESI) m/z calcd for $C_{26}H_{24}N_4O_4$ $[M + H]^+$ 457.187, found 457.1867.

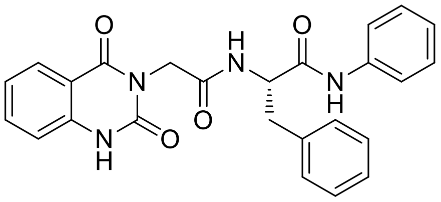

12, ^{1}H NMR (400 MHz, DMSO-d_6) δ 11.49 (s, 1H), 9.98 (s, 1H), 8.65 (d, *J* = 8.0 Hz, 1H), 7.93 (dd, *J* = 12.0, 5.0 Hz, 1H), 7.74 – 7.62 (m, 1H), 7.69 – 7.55 (m, 2H), 7.26 (m, 8H), 7.15 – 6.94 (m, 1H), 4.67 (dd, *J* = 13.9, 8.2 Hz, 1H), 4.65 – 4.40 (m, 2H), 3.07 (dd, *J* = 13.7, 5.5 Hz, 1H), 2.92 (dd, *J* = 10.7, 5.6 Hz, 1H). ^{13}C NMR (100 MHz, DMSO-d_6) δ 170.2, 167.2, 162.8, 162.3, 150.5, 139.9, 139.1, 137.9, 135.6, 129.7, 129.2, 128.6, 127.9, 126.9, 123.9, 123.1, 119.9, 115.7, 114.1, 55.4, 42.9, 38.3; HRMS (ESI) m/z calcd for $C_{25}H_{22}N_4O_4$ $[M + H]^+$ 443.1714, found 443.1711.

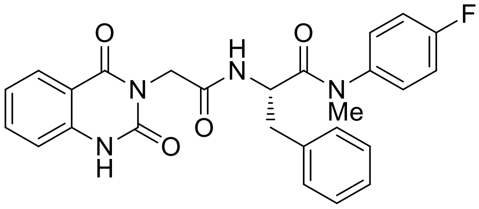

13, ^{1}H NMR (400 MHz, DMSO-d_6) δ 11.45 (s, 1H), 8.66 (d, *J* = 7.2 Hz, 1H), 7.91 (d, *J* = 7.2 Hz, 1H), 7.71 – 7.66 (m, 1H), 7.48 – 7.08 (m, 8H), 6.91 (d, *J* = 6.3 Hz, 2H), 4.67 – 4.21 (m, 3H), 3.10 (s, *J* = 25.7 Hz, 3H), 2.88 (d, *J* = 8.5 Hz, 1H), 2.72- 2.70 (m, 1H). ^{13}C NMR (100 MHz, DMSO-d_6) δ 171.2, 169.6, 166.9, 163.5, 152.8, 150.4, 142.7, 137.9, 137.8, 135.7, 134.5, 133.2, 129.4, 129.1, 128.7, 127.9, 127.0, 125.5, 124.1, 123.1, 119.8, 116.8, 115.7, 55.5, 42.4, 42.1, 37.4; HRMS (ESI) m/z calcd for $C_{26}H_{23}FN_4O_4$ $[M + H]^+$ 475.1776, found 475.1774.

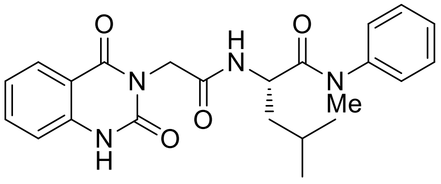

14, ^{1}H NMR (400 MHz, DMSO-d_6) δ 11.40 (s, 1H), 8.34 (d, *J* = 7.7 Hz, 1H), 7.93 (d, *J* = 7.8 Hz, 1H), 7.68 (t, *J* = 7.4 Hz, 1H), 7.52 – 7.30 (m, 5H), 7.23 (t, *J* = 7.8 Hz, 2H), 4.52 – 4.45 (m, 3H), 3.17 (s, 3H), 1.52 – 1.41 (m, 2H), 1.23 – 1.20 (m, 1H), 0.71 (d, *J* = 5.1 Hz, 3H), 0.32 (d, *J* = 3.3 Hz, 3H). ^{13}C NMR (100 MHz, DMSO-d_6) δ 172.2, 166.8, 166.8, 162.2, 150.5, 143.4, 140.0, 135.6, 129.9, 128.0, 127.9, 123.0, 115.6, 114.2, 48.4, 42.6, 41.1, 37.6, 24.4, 23.5, 21.0; HRMS (ESI) m/z calcd for $C_{23}H_{26}N_4O_4$ $[M + H]^+$ 423.2027, found 423.2024.

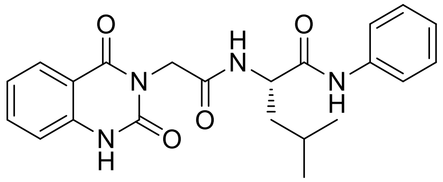

15, ^{1}H NMR (400 MHz, DMSO-d_6) δ 11.50 (s, 1H), 9.92 (s, 1H), 8.49 (d, *J* = 8.0 Hz, 1H), 7.94 (d, *J* = 7.9 Hz, 1H), 7.81 – 7.54 (m, 3H), 7.36– 7.20 (m, 4H), 7.06 (t, *J* = 7.4 Hz, 1H), 4.62 – 4.40 (m, 3H), 1.76 – 1.48 (m, 3H), 0.91 (dd, *J* = 14.8, 6.5 Hz, 6H). ^{13}C NMR (100 MHz, DMSO-d_6) δ 171.3, 167.2, 162.4, 150.6, 139.9, 139.3, 135.6, 129.2, 127.9, 123.9, 123.1, 119.8, 115.7, 114.2, 52.4, 42.9, 41.5, 24.8, 23.5, 22.2; HRMS (ESI) m/z calcd for $C_{22}H_{24}N_4O_4$ $[M + H]^+$ 408.187, found 408.1865.

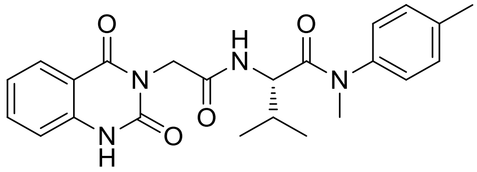

16, ^{1}H NMR (400 MHz, DMSO-d_6) δ 11.46 (s, 1H), 8.32 – 8.23 (m, 1H), 7.99 – 7.87 (m, 2H), 7.71 – 7.67 (m, 1H), 7.26 – 7.16 (m, 5H), 4.70 – 4.46 (m, 3H), 2.90 (s, 3H), 2.72 (s, 3H), 0.89 – 0.67 (m, 6H). ^{13}C NMR (100 MHz, DMSO-d_6) δ 171.5, 166.9, 162.8, 162.2, 150.5, 140.8, 139.9, 137.5, 135.6, 130.4, 127.9, 127.8, 123.0, 115.6, 114.1, 52.0, 42.6, 36.2, 31.2, 26.2, 21.0, 15.1.

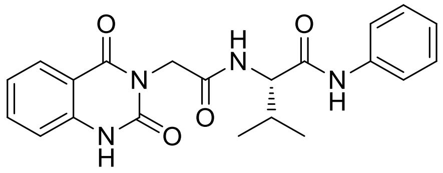

17, ^{1}H NMR (400 MHz, DMSO-d_6) δ 11.48 (s, 1H), 10.06 (s, 1H), 8.45 (d, *J* = 8.8 Hz, 1H), 7.93 (d, *J* = 7.7 Hz, 1H), 7.75 – 7.54 (m, 3H), 7.32 (t, *J* = 7.8 Hz, 2H), 7.22 (t, *J* = 8.6 Hz, 2H), 7.06 (t, *J* = 7.4 Hz, 1H), 4.62 (s, 2H), 4.35 (t, *J* = 8.0 Hz, 1H), 2.16 – 1.87 (m, 1H), 0.93 (dd, *J* = 6.6, 2.6 Hz, 6H). ^{13}C NMR (100 MHz, DMSO-d_6) δ 170.4, 167.3, 162.3, 150.5, 139.9, 139.2, 135.6, 129.2, 127.9, 123.9, 123.1, 119.8, 115.7, 114.2, 59.1, 42.8, 31.5, 19.6, 18.8; HRMS (ESI) m/z calcd for $C_{21}H_{22}N_4O_4$ $[M + H]^+$ 395.1714, found 395.1718.

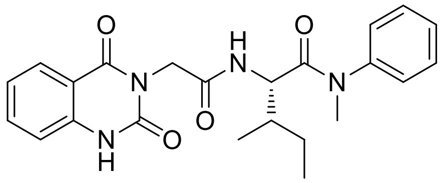

18, ^{1}H NMR (600 MHz, DMSO-d_6) δ 11.46 (s, 1H), 8.22 (d, *J* = 7.2 Hz, 1H), 7.92 (s, 1H), 7.69 (d, *J* = 7.8 Hz, 1H), 7.41 – 7.22 (m, 5H), 7.28 – 7.13 (m, 2H), 4.60 – 4.38 (m, 3H), 3.18 (s, 3H), 1.61- 1.54 (m, 1H), 0.86 – 0.84 (m, 2H), 0.79 (d, *J* = 6.8 Hz, 3H), 0.36 (t, 3H); HRMS (ESI) m/z calcd for $C_{23}H_{26}N_4O_4$ $[M + H]^+$ 423.2027, found 423.2031.

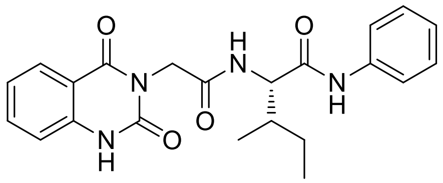

19, ^{1}H NMR (600 MHz, DMSO-d_6) δ 11.52 (s, 1H), 10.04 (s, 1H), 8.43 – 8.35 (m, 1H), 7.91 (d, *J* = 7.8 Hz, 1H), 7.66 (t, *J* = 7.6 Hz, 1H), 7.61 (d, *J* = 7.9 Hz, 2H), 7.29 (t, *J* = 7.7 Hz, 2H), 7.21 – 7.17 (m, 2H), 7.04 (t, *J* = 7.3 Hz, 1H), 4.61 (s, 2H), 4.47 (t, *J* = 6.6 Hz, 1H), 1.86 – 1.82 (m, 1H),

1.40 – 1.35 (m, 1H), 1.18-1.13 (m, 1H), 0.96 – 0.84 (m, 6H)); HRMS (ESI) m/z calcd for $C_{22}H_{24}N_4O_4$ $[M + H]^+$ 409.187, found 409.1872.

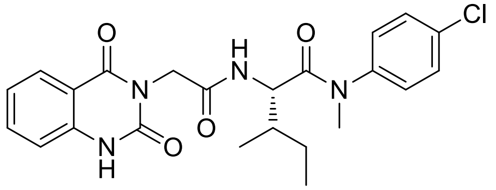

20, ^{1}H NMR (400 MHz, DMSO-*d6*) δ 11.47 (s, 1H), 8.44 – 8.27 (m, 1H), 8.01 – 7.86 (m, 2H), 7.71 – 7.65 (m, 1H), 7.50 – 7.45 (m, 1H), 7.38 – 7.34 (m, 1H), 7.25 – 7.20 (m, 3H), 4.60 – 4.45 (m, 3H), 2.90 (s, 3H), 1.18 – 1.02 (m, 3H), 0.90 – 0.68 (m, 6H). ^{13}C NMR (100 MHz, DMSO-*d6*) δ 167.1, 162.8, 162.2, 150.5, 139.9, 135.6, 129.9, 127.9, 123.1, 115.6, 114.1, 53.8, 42.7, 36.0, 31.2, 21.2, 15.1, 12.1; HRMS (ESI) m/z calcd for $C_{23}H_{25}ClN_4O_4$ $[M + H]^+$ 457.1637, found 457.1638.

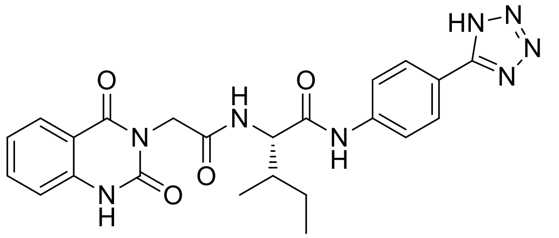

21, ^{1}H NMR (600 MHz, DMSO-*d6*) δ 11.49 (s, 1H), 10.30 (s, 1H), 8.54 – 8.44 (m, 2H), 7.92 – 7.86 (m, 1H), 7.82 – 7.60 (m, 1H), 7.53 – 8.44 (m, 1H), 7.19 (s, 2H), 4.69 – 4.51 (m, 2H), 4.00 (d, *J* = 6.8 Hz, 1H), 2.06 – 1.76 (m, 2H), 1.39 – 1.32 (m, 1H), 1.25 – 1.10 (m, 2H), 0.92 – 0.85 (m, 4H); HRMS (ESI) m/z calcd for $C_{23}H_{24}N_8O_4$ $[M + H]^+$ 477.1993, found 477.1991.

Scheme 2: Synthesis of intermediates f-g and compounds 22-39

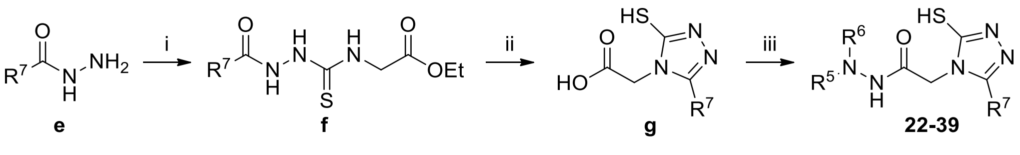

Reagents and conditions: (i) ethyl isothiocyanatoacetate, ACN, 40 °C, 3 h; (ii) NaOH, H_2O, 100 °C, 3 h; (iii) hydrazine, HATU, DIPEA, DMF, rt, 24 h.

***Synthesis of intermediate f*:** Hydrazide (1.0 g, 1 equiv.) and isothiocyanato ethyl acetate (1.6 g, 1.5 equiv.) were mixed in CH_3CN and stirred for 3 hours at 40 °C. Upon completion, reaction mixture was cooled down and the white solid precipitated was filtered and washed with ether to get pure compound **f** (1.8 g). The desired product was confirmed from NMR and mass.

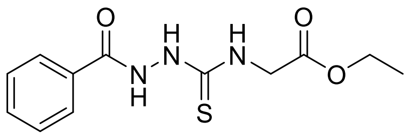

^{1}H NMR (600 MHz, DMSO-d_6) δ 10.47 (s, 1H), 9.60 (s, 1H), 8.36 (s, 1H), 7.92 (d, *J* = 7.5 Hz, 2H), 7.56 (d, *J* = 7.5 Hz, 1H), 7.48 (t, *J* = 7.7 Hz, 2H), 4.28 – 4.08 (m, 3H), 4.07 (d, *J* = 7.1 Hz, 1H), 1.18 (t, *J* = 7.1 Hz, 3H).

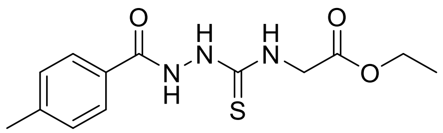

^{1}H NMR (600 MHz, DMSO-d_6) δ 10.38 (s, 1H), 9.56 (s, 1H), 8.33 (s, 1H), 7.81 (d, *J* = 8.0 Hz, 2H), 7.28 (d, *J* = 8.0 Hz, 2H), 4.10 (dd, *J* = 29.9, 22.8 Hz, 4H), 2.35 (s, 3H), 1.18 (t, *J* = 7.1 Hz, 3H).

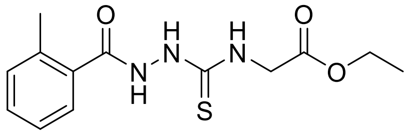

^{1}H NMR (600 MHz, DMSO-d_6) δ 10.15 (s, 1H), 9.62 (s, 1H), 8.18 (s, 1H), 7.61 (s, 1H), 7.36 (t, *J* = 7.4 Hz, 1H), 7.24 (t, *J* = 8.2 Hz, 2H), 4.22 (s, 2H), 4.09 (q, *J* = 7.1 Hz, 2H), 2.37 (s, 3H), 1.19 (t, *J* = 7.1 Hz, 3H).

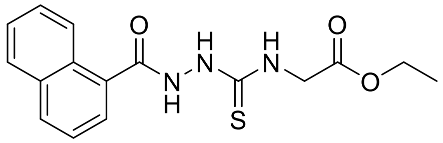

^{1}H NMR (600 MHz, DMSO-d_6) δ 10.63 (s, 1H), 9.67 (s, 1H), 8.56 (s, 1H), 8.44 (s, 1H), 8.00 (dt, *J* = 19.7, 6.2 Hz, 4H), 7.61 (dt, *J* = 14.8, 6.9 Hz, 2H), 4.19 (s, 2H), 4.09 (q, *J* = 7.0 Hz, 2H), 1.18 (t, *J* = 7.1 Hz, 3H).

Synthesis of intermediate g: To the solution of compound **f** (1.5 g, 1.0 equiv.) in water (15 mL) was added sodium hydroxide (2.5 equiv.) and the mixture was stirred for 3 hours at 100 °C. Upon completion, confirmed by TLC, the reaction mixture was cooled to room temperature and 1N HCl was slowly added until pH = 3, then precipitated white powder was filtered and dried to yielded acid (0.9 g). The desired product was confirmed from NMR and mass spectrum.

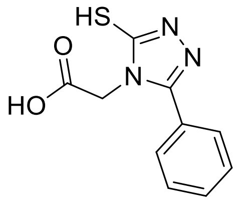

^{1}H NMR (600 MHz, DMSO-d_6) δ 7.80 – 7.58 (m, 3H), 7.58 – 7.48 (m, 3H), 4.80 (s, 2H).

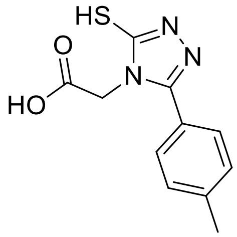

^{1}H NMR (600 MHz, DMSO-d_6) δ 7.50 (d, *J* = 8.1 Hz, 2H), 7.35 (d, *J* = 8.0 Hz, 2H), 4.79 (s, 2H), 2.36 (s, 3H).

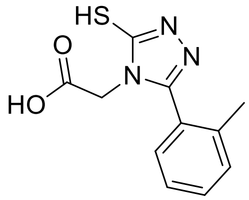

^{1}H NMR (600 MHz, DMSO-d_6) δ 13.23 (s, 1H), 7.46 (t, J = 7.5 Hz, 1H), 7.38 (d, J = 8.0 Hz, 2H), 7.33 (t, J = 7.5 Hz, 1H), 4.73 – 4.32 (m, 2H), 2.26 (s, 3H).

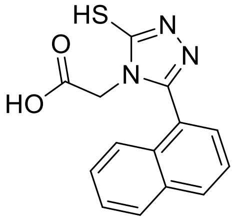

^{1}H NMR (600 MHz, DMSO-d_6) δ 13.32 (s, 1H), 8.25 (s, 1H), 8.08 (d, J = 8.5 Hz, 1H), 8.01 (t, J = 8.0 Hz, 2H), 7.73 – 7.67 (m, 1H), 7.67 – 7.56 (m, 2H), 4.94 (s, 2H).

Synthesis of compounds 22-39: To the solution of acid (1.0 equiv.) in DMF (5 mL) was added HATU (1.0 equiv.) and the mixture was stirred for 30 min at room temperature. To the mixture, hydrazine (1.5 equiv.) and DIPEA (2.2 equiv.) were added and stirred at room temperature for 12 h. and reaction was monitored by TLC. The reaction was diluted with water and extracted with EtOAc (3x25 mL) and washed with brine, organic layer was dried over Na_2SO_4, filtered and concentrated. The crude product was purified by flash column chromatography (EtOAc/hexane; 1:1) to afford desired compound as a white solid. The desired product was confirmed from NMR spectrum and mass spectrum.

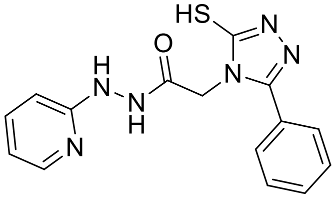

22, ^{1}H NMR (600 MHz, DMSO-d_6) δ 10.15 (s, 1H), 9.48 (s, 1H), 8.36 (s, 1H), 8.02 (d, J = 4.2 Hz, 1H), 7.67 (d, J = 7.3 Hz, 2H), 7.62 – 7.57 (m, 2H), 7.55 – 7.48 (m, 3H), 7.43 (d, J = 7.1 Hz, 1H), 6.72 – 6.63 (m, 1H), 6.41 (d, J = 8.3 Hz, 1H), 4.81 (s, 2H); HRMS (ESI) m/z calcd for $C_{15}H_{14}N_6OS$ $[M + H]^+$ 327.1023, found 327.1028.

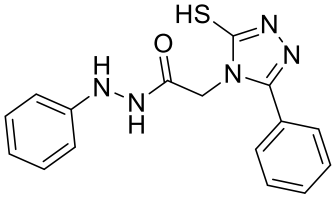

23, ^{1}H NMR (600 MHz, DMSO-*d*$_6$) δ 10.03 (d, *J* = 2.0 Hz, 1H), 7.78 (d, *J* = 1.9 Hz, 1H), 7.68 – 7.50 (m, 7H), 7.17 (t, *J* = 7.8 Hz, 1H), 7.06 (t, *J* = 7.8 Hz, 2H), 6.66 (t, *J* = 7.3 Hz, 2H), 6.58 (d, *J* = 7.8 Hz, 2H), 4.82 (s, 2H); HRMS (ESI) m/z calcd for $C_{16}H_{15}N_5OS$ $[M + H]^+$ 326.107, found 326.1073.

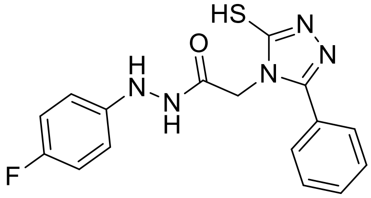

24, ^{1}H NMR (600 MHz, DMSO-*d*$_6$) δ 10.06 (s, 1H), 7.77 (d, *J* = 2.3 Hz, 1H), 7.69 – 7.48 (m, 7H), 7.02 (t, *J* = 8.8 Hz, 1H), 6.90 (t, *J* = 8.8 Hz, 2H), 6.67 (s, 1H), 6.62 – 6.53 (m, 2H), 4.81 (s, 2H); HRMS (ESI) m/z calcd for $C_{16}H_{14}FN_5OS$ $[M + H]^+$ 344.0976, found 344.0981.

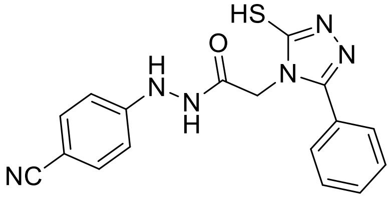

25, ^{1}H NMR (600 MHz, DMSO-*d*$_6$) δ 10.28 (s, 1H), 8.66 (s, 1H), 7.63 (dt, *J* = 25.6, 8.9 Hz, 4H), 7.59 – 7.51 (m, 3H), 7.48 (d, *J* = 8.6 Hz, 2H), 6.63 (d, *J* = 8.7 Hz, 2H), 4.85 (s, 2H); HRMS (ESI) m/z calcd for $C_{17}H_{14}N_6OS$ $[M + H]^+$ 351.1023, found 351.1024.

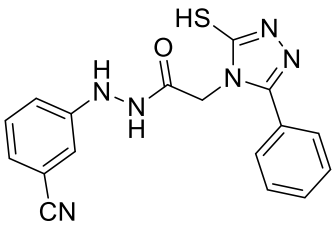

26, ^{1}H NMR (600 MHz, DMSO-d_6) δ 10.23 (d, J = 1.6 Hz, 1H), 8.32 (s, 1H), 7.84 – 7.60 (m, 3H), 7.60 – 7.48 (m, 4H), 7.28 (t, J = 7.9 Hz, 1H), 7.09 (d, J = 7.5 Hz, 1H), 7.02 (s, 1H), 6.92 (dd, J = 8.3, 2.0 Hz, 1H), 4.82 (s, 2H); HRMS (ESI) m/z calcd for $C_{17}H_{14}N_6OS$ $[M + H]^+$ 351.1023, found 351.1028.

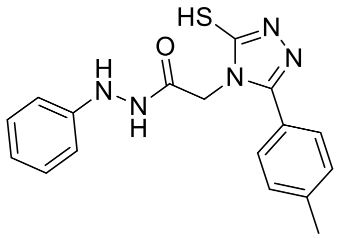

27, ^{1}H NMR (600 MHz, DMSO-d_6) δ 10.02 (s, J = 1.9 Hz, 1H), 7.79 (d, J = 1.8 Hz, 1H), 7.52 (t, J = 16.8 Hz, 2H), 7.45 (d, J = 8.0 Hz, 1H), 7.38 – 7.31 (m, 3H), 7.17 (t, J = 7.8 Hz, 1H), 7.16 (d, J = 7.8 Hz, 2H), 6.67 (t, J = 6.9 Hz, 2H), 6.59 (d, J = 7.8 Hz, 2H), 2.39 (s, 2H); HRMS (ESI) m/z calcd for $C_{17}H_{17}N_5OS$ $[M + H]^+$ 340.1227, found 340.1229.

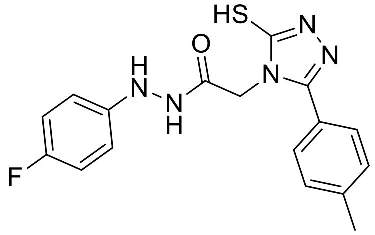

28, ^{1}H NMR (600 MHz, DMSO-d_6) δ 10.06 (d, J = 2.5 Hz, 1H), 7.53 (d, J = 8.1 Hz, 2H), 7.43 (t, J = 17.3 Hz, 1H), 7.39 (m, 4H), 7.28 (d, J = 8.1 Hz, 1H), 7.16 – 6.94 (m, 1H), 6.91 (t, J = 8.8 Hz, 2H), 6.68 (dd, J = 8.8, 4.5 Hz, 1H), 6.65 – 6.56 (m, 2H), 4.79 (s, 2H), 2.38 (s, 3H); HRMS (ESI) m/z calcd for $C_{17}H_{16}FN_5OS$ $[M + H]^+$ 358.1132, found 358.1137.

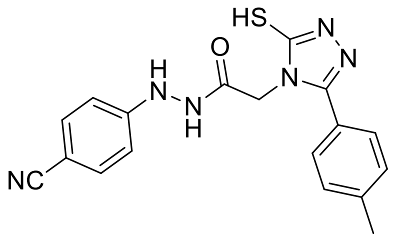

29, ^{1}H NMR (600 MHz, DMSO-d_6) δ 10.29 (s, 1H), 8.68 (s, 1H), 7.57 – 7.43 (m, 4H), 7.36 (d, *J* = 6.3 Hz, 2H), 6.67 (d, *J* = 7.2 Hz, 2H), 4.82 (s, 2H), 2.40 (s, 3H); HRMS (ESI) m/z calcd for $C_{18}H_{16}N_6OS$ [M + H]$^+$ 365.1179, found 365.1179.

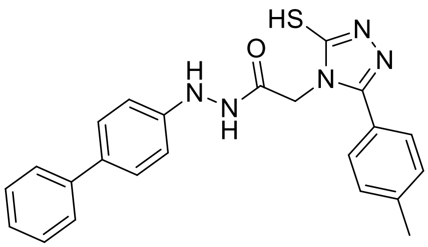

30, ^{1}H NMR (600 MHz, DMSO-d_6) δ 10.95 (s, 1H), 7.51 (d, *J* = 8.0 Hz, 2H), 7.40 – 7.32 (m, 4H), 7.23 (t, *J* = 7.8 Hz, 4H), 7.02 – 6.88 (m, 6H), 4.85 (s, 2H), 2.39 (s, 3H); HRMS (ESI) m/z calcd for $C_{23}H_{21}N_5OS$ [M + H]$^+$ 416.154, found 416.1545.

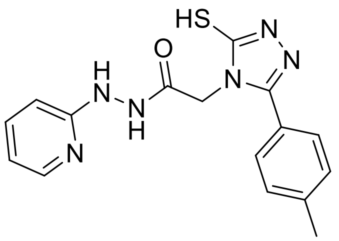

31, ^{1}H NMR (600 MHz, DMSO-d_6) δ 10.15 (s, 1H), 8.37 (s, 1H), 8.03 (s, 1H), 7.54 (t, *J* = 17.6 Hz, 2H), 7.35 (d, *J* = 7.7 Hz, 3H), 6.68 (s, 1H), 6.44 (d, *J* = 8.0 Hz, 1H), 4.80 (s, 2H), 2.34 (s, 3H); HRMS (ESI) m/z calcd for $C_{16}H_{16}N_6OS$ [M + H]$^+$ 341.1179, found 341.1174.

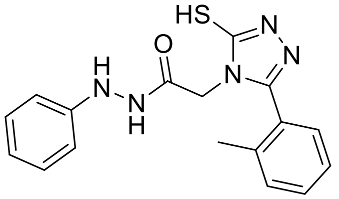

32, ^{1}H NMR (600 MHz, DMSO-d_6) δ 9.87 (s, 1H), 7.71 (s, 1H), 7.53 – 7.41 (m, 3H), 7.34 (ddd, *J* = 21.9, 17.6, 7.7 Hz, 4H), 7.07 (dt, *J* = 24.1, 6.8 Hz, 3H), 6.66 (t, *J* = 7.0 Hz, 1H), 6.47 (t, *J* = 10.0 Hz, 3H), 4.59 (s, 2H), 2.20 (s, 3H); HRMS (ESI) m/z calcd for $C_{17}H_{17}N_5OS$ [M + H]$^+$ 340.1227, found 340.1225.

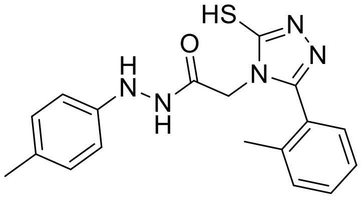

33, ^{1}H NMR (600 MHz, DMSO-*d*$_6$) δ 9.83 (d, *J* = 2.1 Hz, 1H), 7.53 (s, 1H), 7.49 (t, *J* = 7.5 Hz, 2H), 7.45 (d, *J* = 7.6 Hz, 1H), 7.38 (dd, *J* = 12.3, 8.6 Hz, 2H), 7.32 (dt, *J* = 10.3, 7.1 Hz, 2H), 6.86 (d, *J* = 8.1 Hz, 2H), 6.41 (d, *J* = 6.1 Hz, 1H), 4.56 (s, 2H), 2.21 (s, 3H), 2.16 (s, 3H); HRMS (ESI) m/z calcd for $C_{18}H_{19}N_5OS$ $[M + H]^+$ 354.1383, found 354.1381.

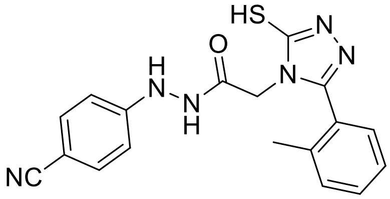

34, ^{1}H NMR (600 MHz, DMSO-*d*$_6$) δ 10.14 (s, 1H), 8.59 (s, 1H), 7.47 (d, *J* = 8.5 Hz, 4H), 7.39 (dd, *J* = 21.2, 7.4 Hz, 1H), 7.36 (s, 1H), 6.55 (t, *J* = 9.7 Hz, 2H), 4.61 (s, 2H), 2.20 (s, 3H); HRMS (ESI) m/z calcd for $C_{18}H_{16}N_6OS$ $[M + H]^+$ 365.1179, found 365.1178.

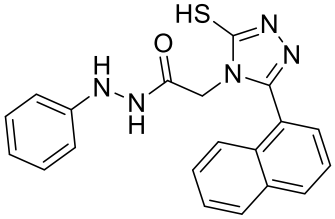

35, ^{1}H NMR (600 MHz, DMSO-*d*$_6$) δ 10.03 (s, 1H), 8.27 (s, 1H), 8.07 – 7.94 (m, 4H), 7.76 (s, 2H), 7.65 (s, 1H), 6.86 (s, 2H), 6.59 (s, 2H), 6.46 (d, *J* = 5.9 Hz, 2H), 4.98 (s, 2H); HRMS (ESI) m/z calcd for $C_{20}H_{17}N_5OS$ $[M + H]^+$ 376.1227, found 376.1226.

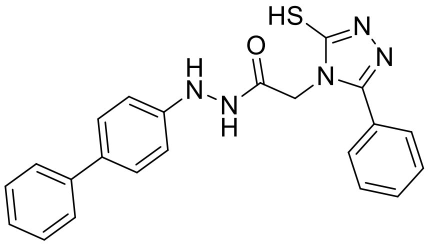

36, ^{1}H NMR (600 MHz, DMSO-*d*$_6$) δ 10.95 (s, 1H), 7.62 (dd, *J* = 11.7, 7.5 Hz, 3H), 7.52 (t, *J* = 7.6 Hz, 3H), 7.36 (dd, *J* = 34.0, 26.2 Hz, 2H), 7.23 (t, *J* = 7.8 Hz, 3H), 7.12 – 7.03 (m, 2H), 6.96 (t, *J* = 8.0 Hz, 3H), 4.87 (s, 2H); HRMS (ESI) m/z calcd for $C_{22}H_{19}N_5OS$ $[M + H]^+$ 402.1383, found 402.1388.

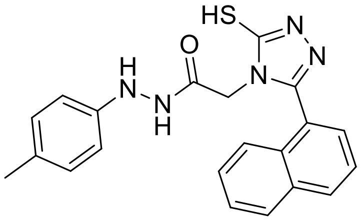

37, ^{1}H NMR (600 MHz, DMSO-*d*$_6$) δ 9.98 (s, *J* = 2.4 Hz, 1H), 8.25 (s, 1H), 8.08 – 7.98 (m, 6H), 7.64 (dd, *J* = 12.4, 4.7 Hz, 3H), 7.62 – 7.56 (m, 3H), 6.66 (d, *J* = 8.2 Hz, 2H), 6.37 (d, *J* = 8.3 Hz, 2H), 4.92 (s, 2H), 2.10 (s, 3H); HRMS (ESI) m/z calcd for $C_{21}H_{19}N_5OS$ $[M + H]^+$ 390.1383, found 390.1382.

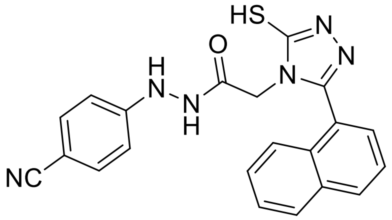

38, ^{1}H NMR (600 MHz, DMSO-*d*$_6$) δ 10.28 (s, 1H), 8.63 (s, 1H), 8.27 (s, 1H), 8.08 (dd, *J* = 20.8, 8.3 Hz, 2H), 8.00 (t, *J* = 12.7 Hz, 1H), 7.71 – 7.61 (m, 3H), 7.21 (d, *J* = 8.0 Hz, 2H), 6.49 (d, *J* = 8.1 Hz, 2H), 5.00 (s, 2H); HRMS (ESI) m/z calcd for $C_{21}H_{16}N_6OS$ $[M + H]^+$ 401.1179, found 401.1183.

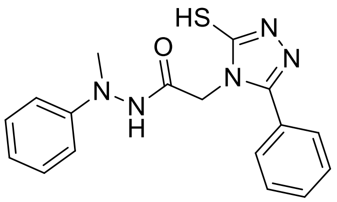

39, ^{1}H NMR (600 MHz, DMSO-d_6) δ 10.35 (s, 1H), 7.70 – 7.66 (m, 2H), 7.63 – 7.58 (m, 1H), 7.60 – 7.53 (m, 2H), 7.14 (dd, J = 8.6, 7.1 Hz, 2H), 6.74 (t, J = 7.3 Hz, 1H), 6.69 (d, J = 8.4 Hz, 2H), 4.83 (s, 2H), 3.33 (d, J = 2.2 Hz, 3H).

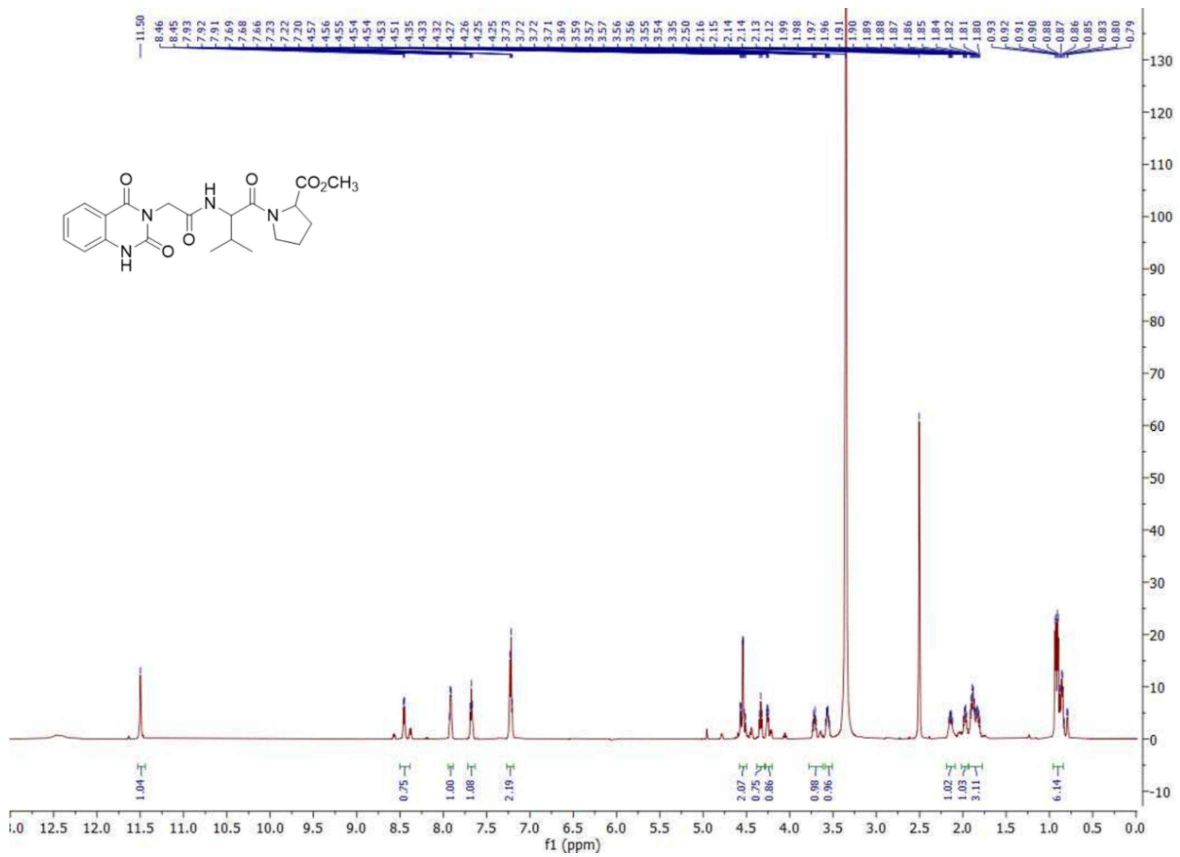

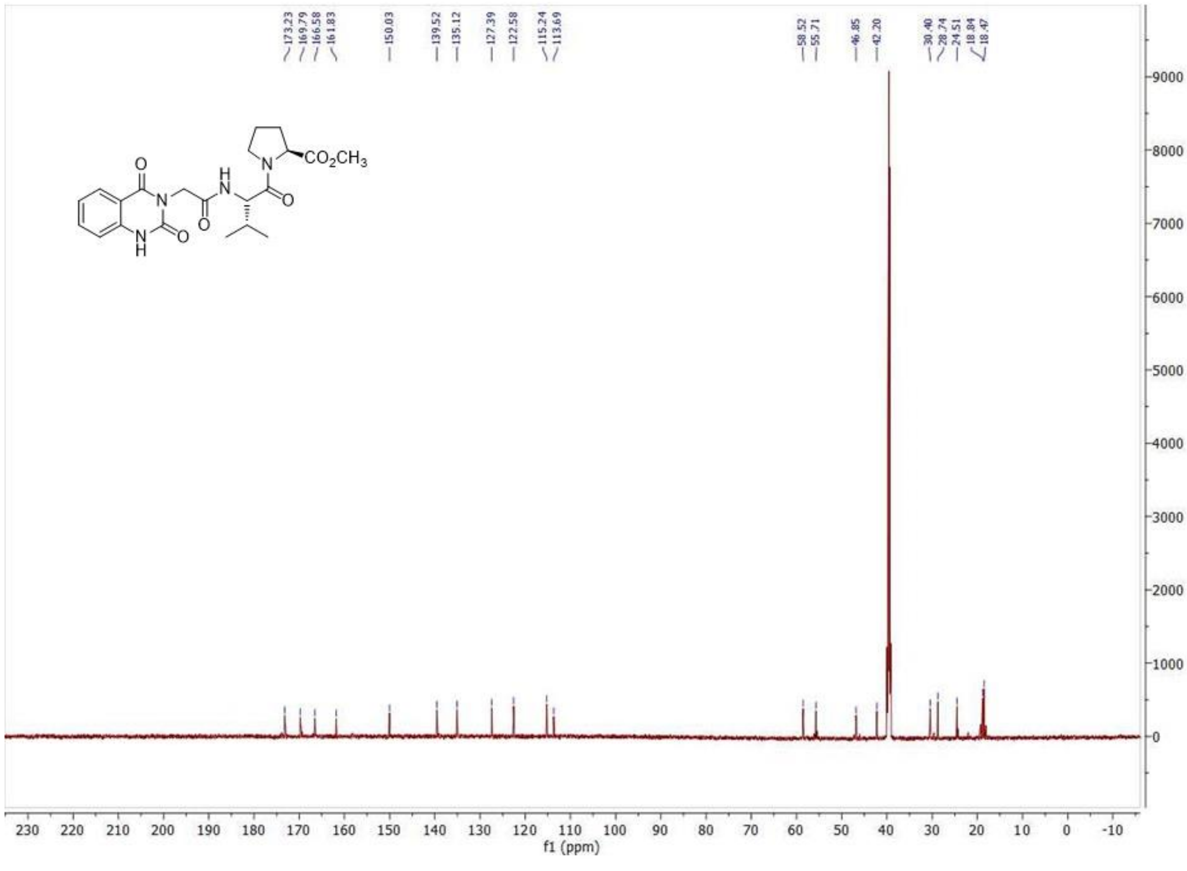

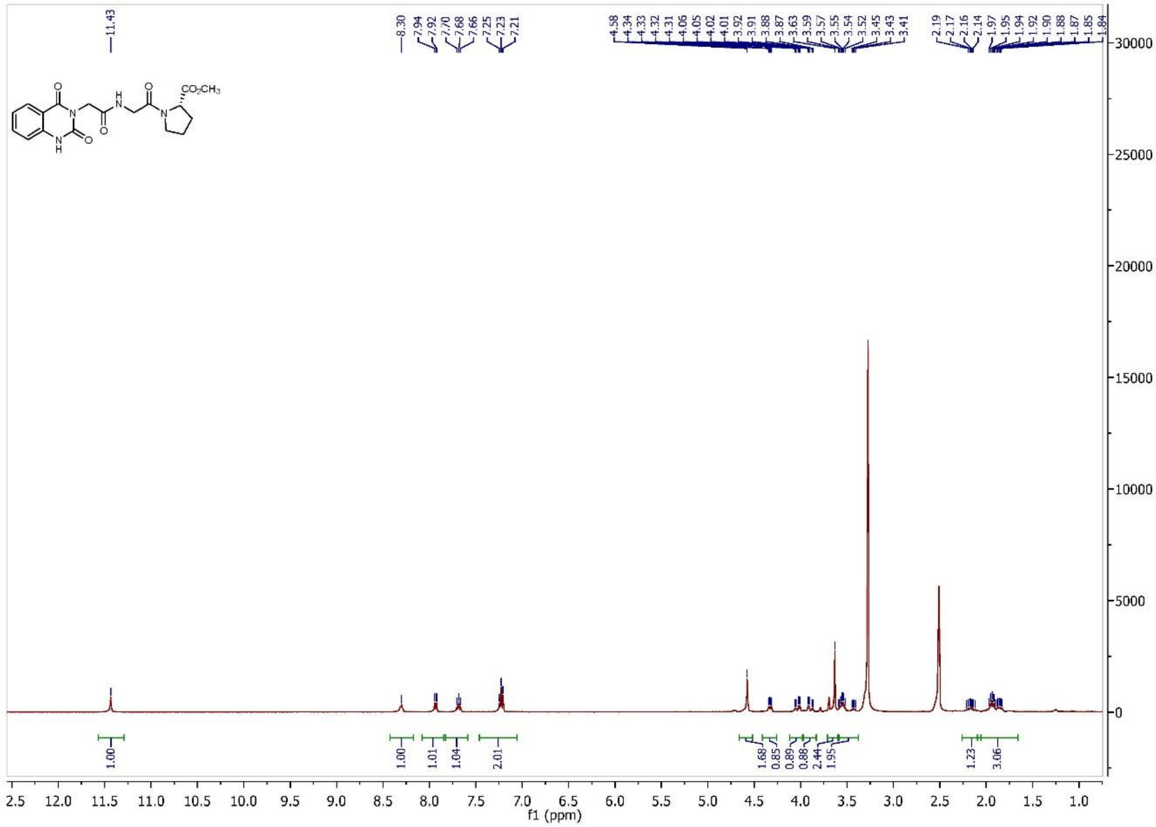

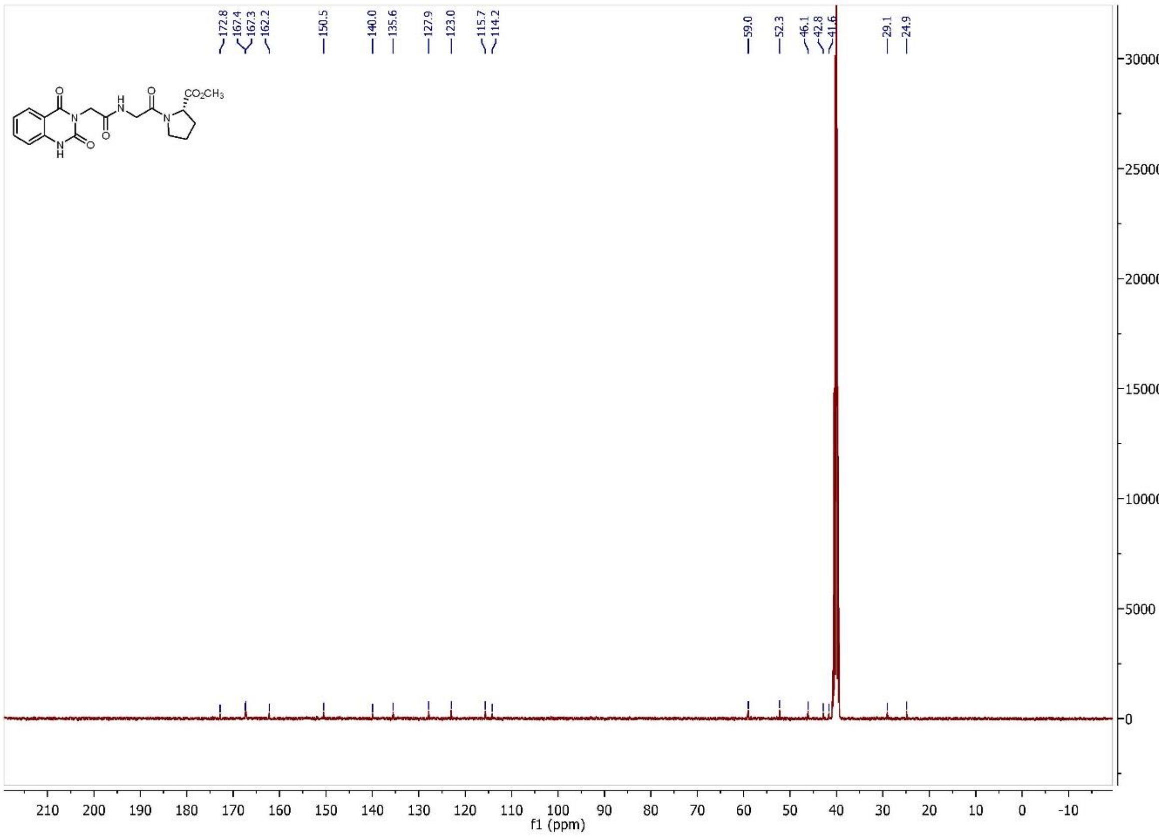

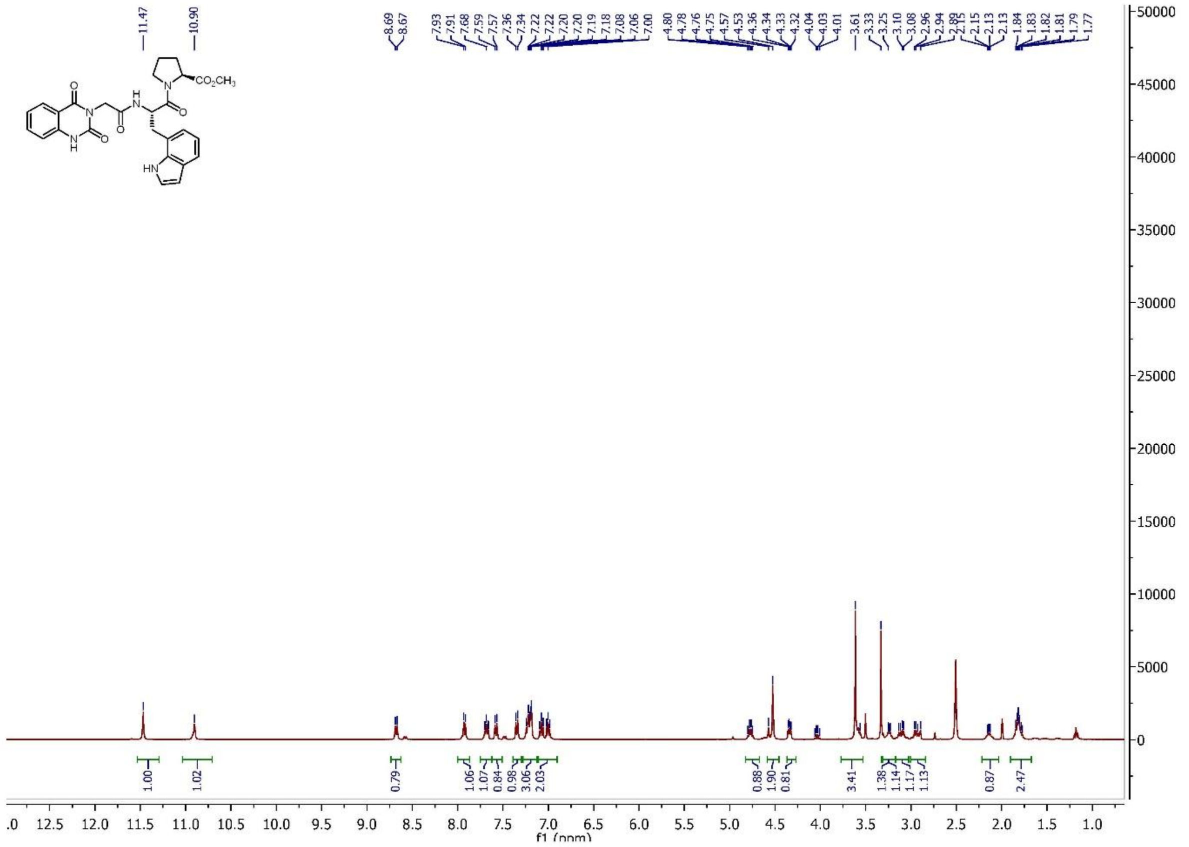
11.47
10.90
8.69 8.67
7.93 7.91 7.68 7.59 7.57 7.36 7.34 7.22 7.22 7.20 7.20 7.19 7.18 7.08 7.06 7.00
4.80 4.78 4.76 4.75 4.57 4.53 4.36 4.34 4.33 4.32 4.04 4.03 4.01
3.61 3.33 3.25 3.10 3.08 2.96 2.94 2.89 2.15 2.15 2.13 2.13 1.84 1.83 1.82 1.81 1.79 1.77
CO2CH3
1.00 1.02 0.79 1.06 1.07 0.84 0.98 3.06 2.03 0.88 1.90 0.81 3.41 1.38 1.14 1.17 1.13 0.87 2.47
f1 (ppm)

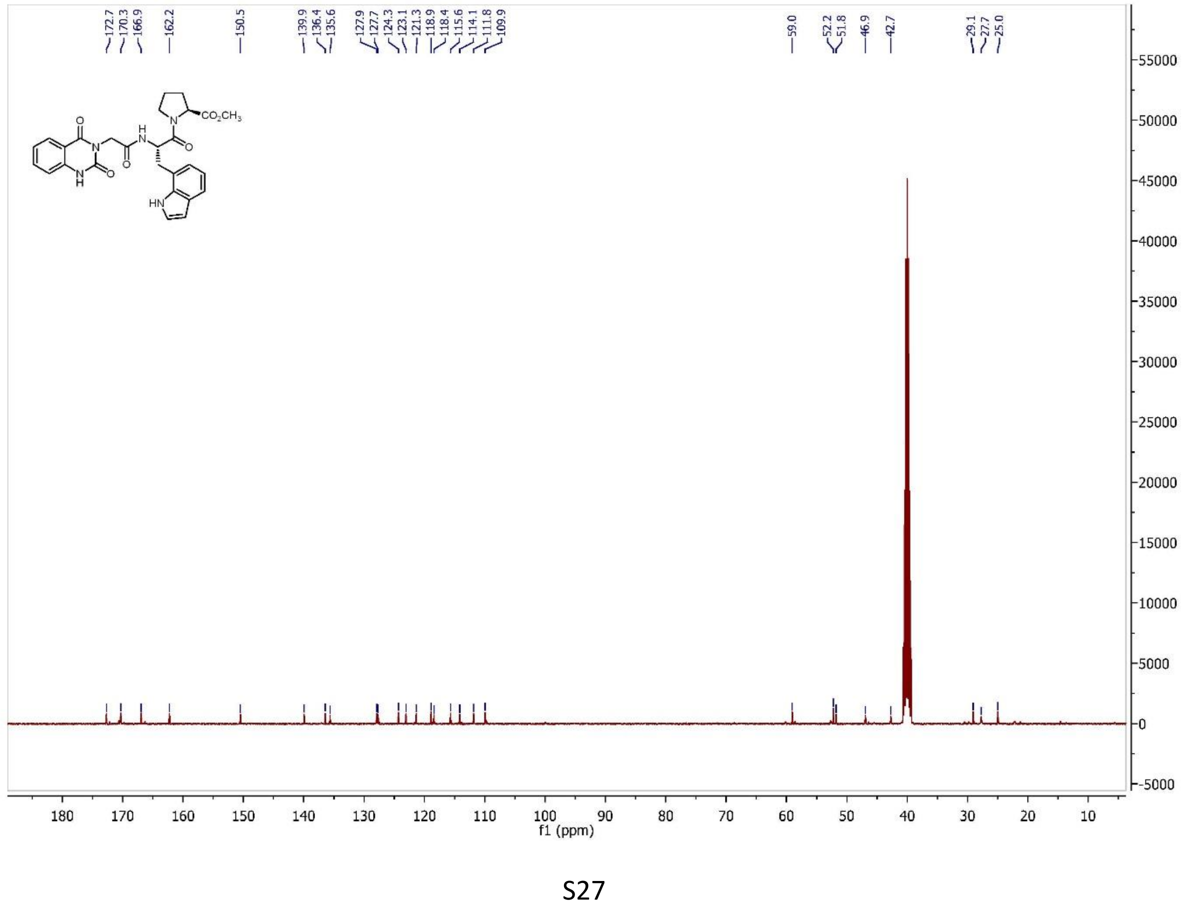
172.7 170.3 166.9 162.2 150.5
139.9 136.4 135.6 127.9 127.7 124.3 123.1 121.3 118.9 118.4 115.6 114.1 111.8 109.9
59.0 52.2 51.8 46.9 42.7 29.1 27.7 25.0
CO2CH3
f1 (ppm)

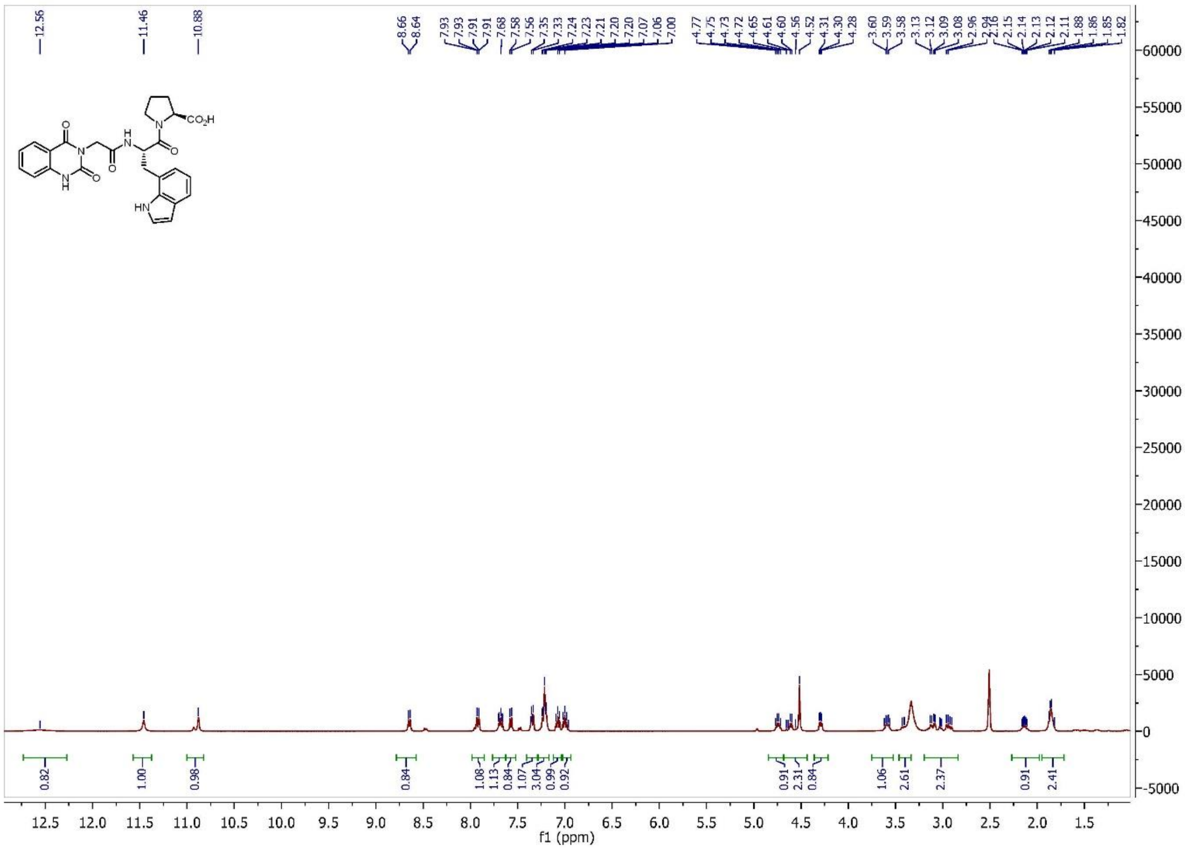

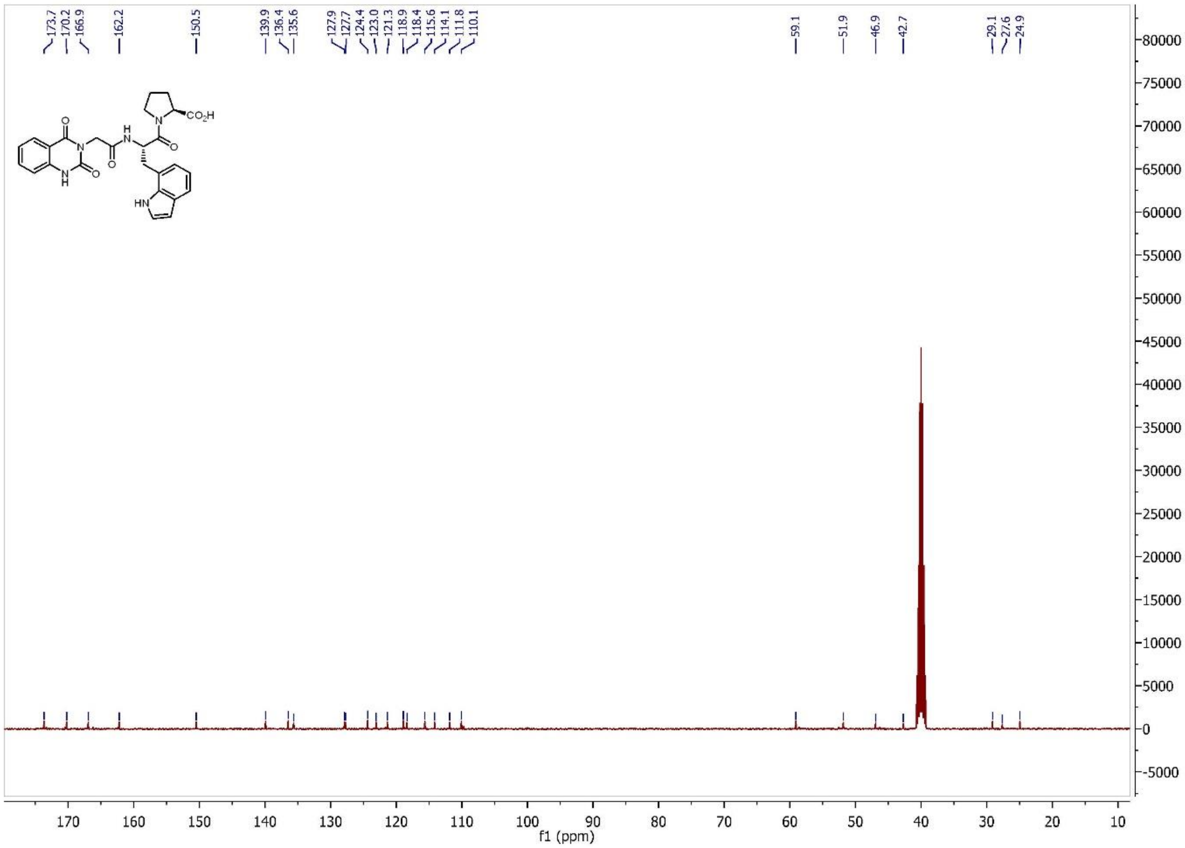

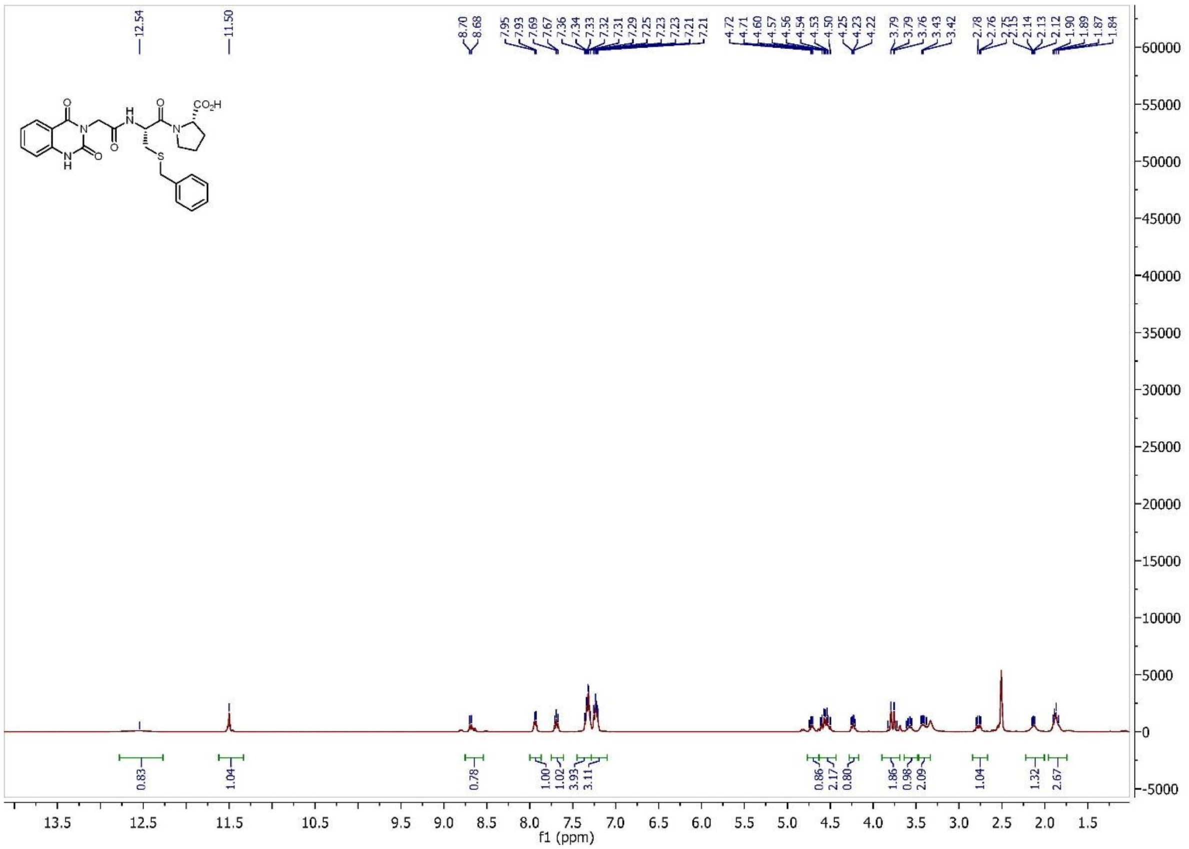

12.54
11.50
8.70
8.68
7.95
7.93
7.69
7.67
7.36
7.34
7.33
7.32
7.31
7.29
7.25
7.23
7.23
7.21
7.21
4.72
4.71
4.60
4.57
4.56
4.54
4.53
4.50
4.25
4.23
4.22
3.79
3.79
3.76
3.43
3.42
2.78
2.76
2.75
2.15
2.14
2.13
2.12
1.90
1.89
1.87
1.84
CO2H
N
H
O
S
0.83
1.04
0.78
1.00
1.02
3.93
3.11
0.86
2.17
0.80
1.86
0.98
2.09
1.04
1.32
2.67
f1 (ppm)

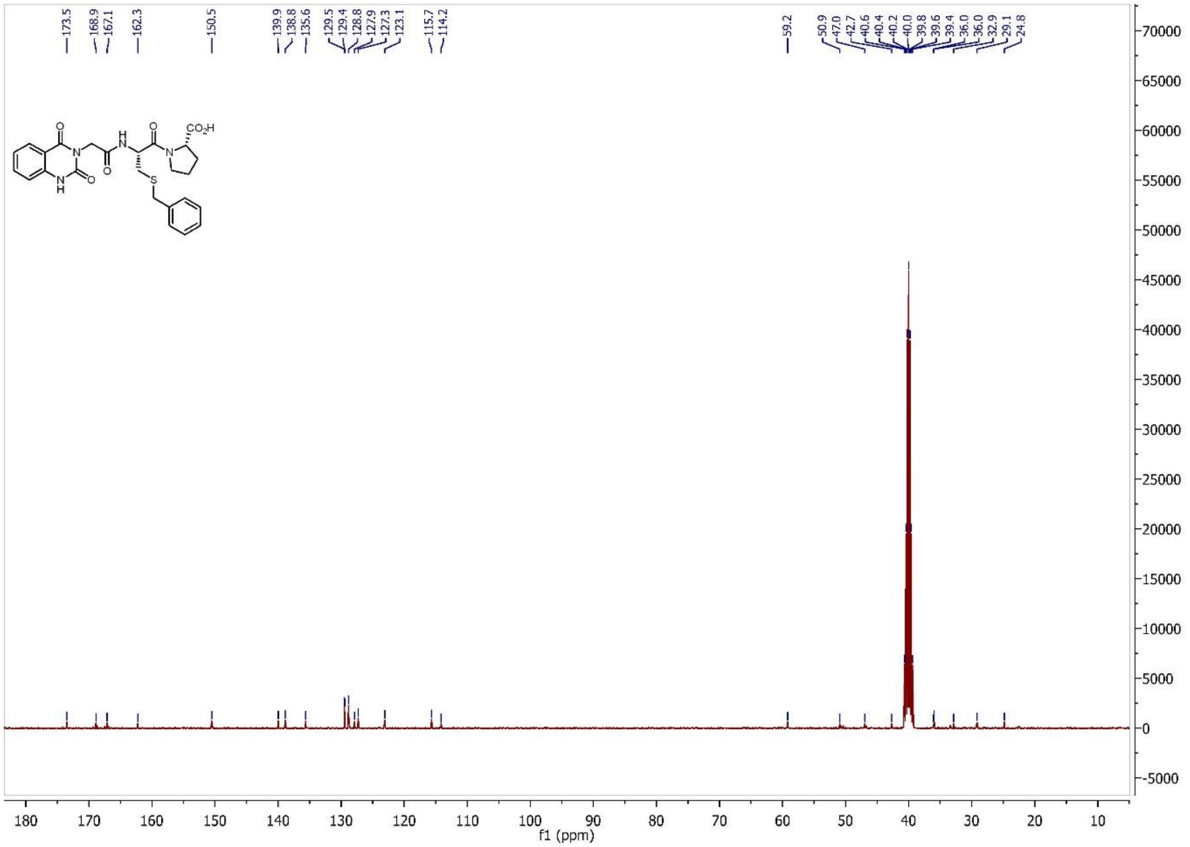

173.5
168.9
167.1
162.3
150.5
139.9
138.8
135.6
129.5
129.4
128.8
127.9
127.3
123.1
115.7
114.2
59.2
50.9
47.0
42.7
40.6
40.4
40.2
40.0
39.8
39.6
39.4
36.0
32.9
29.1
24.8
CO2H
f1 (ppm)

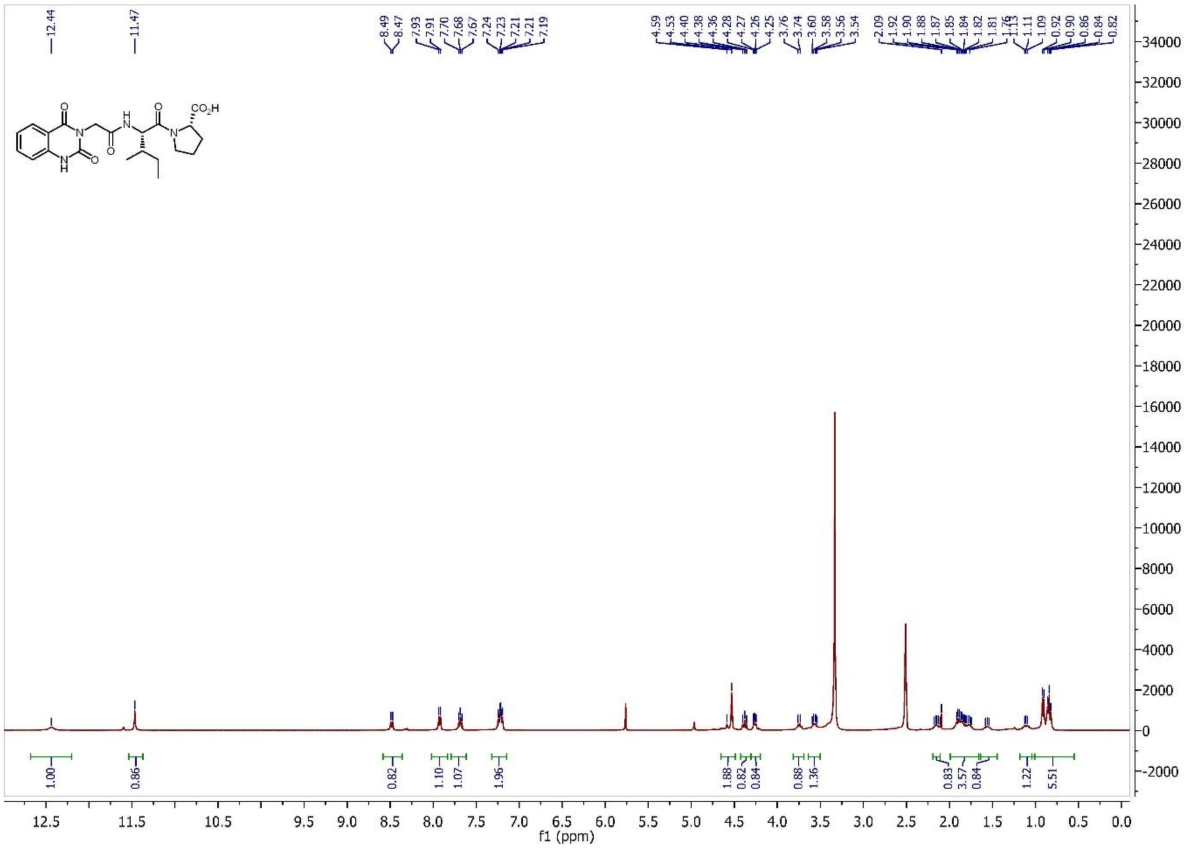

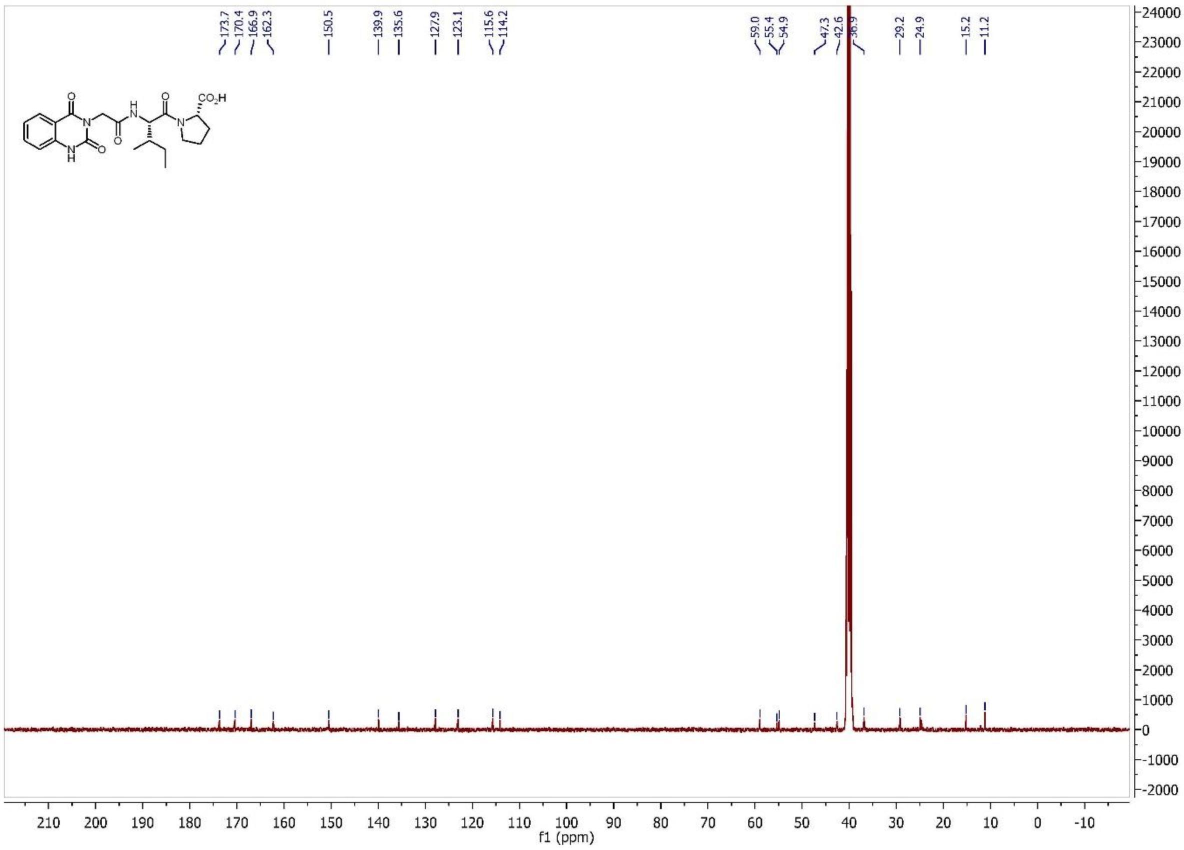

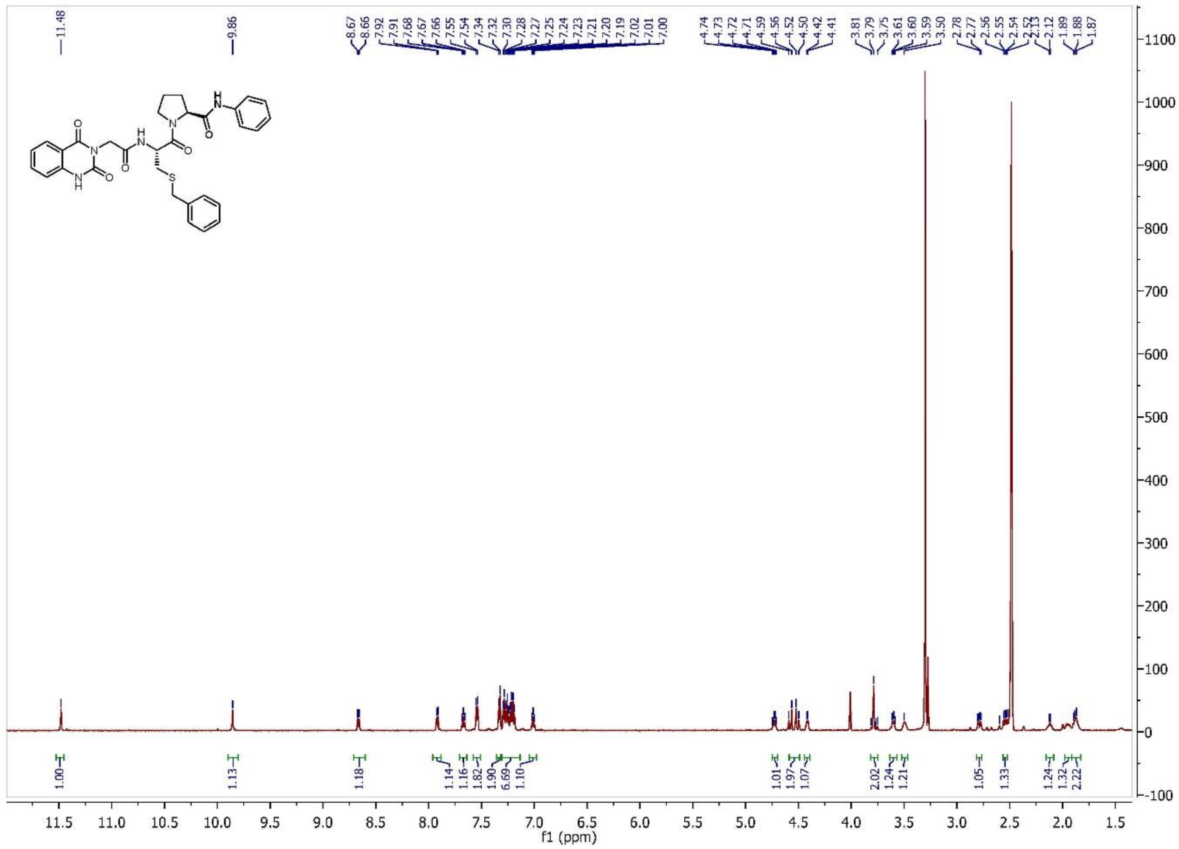

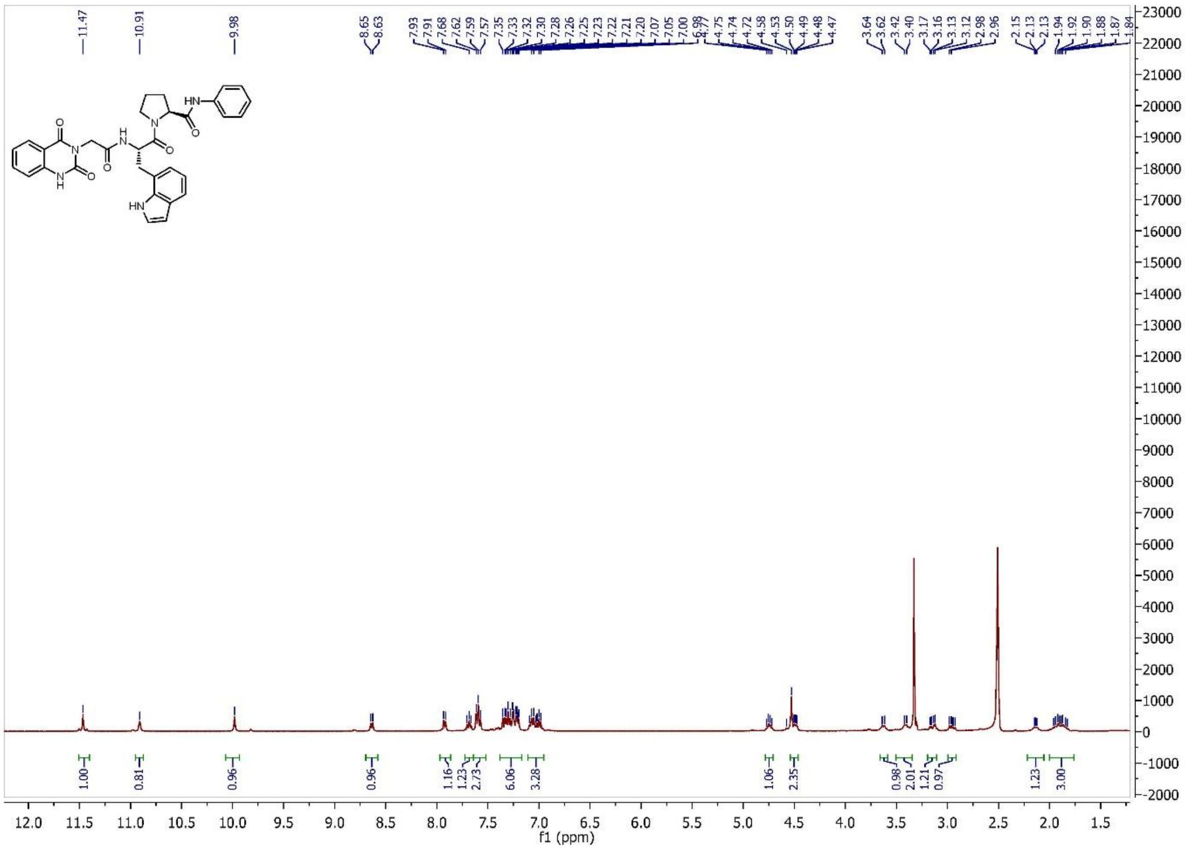

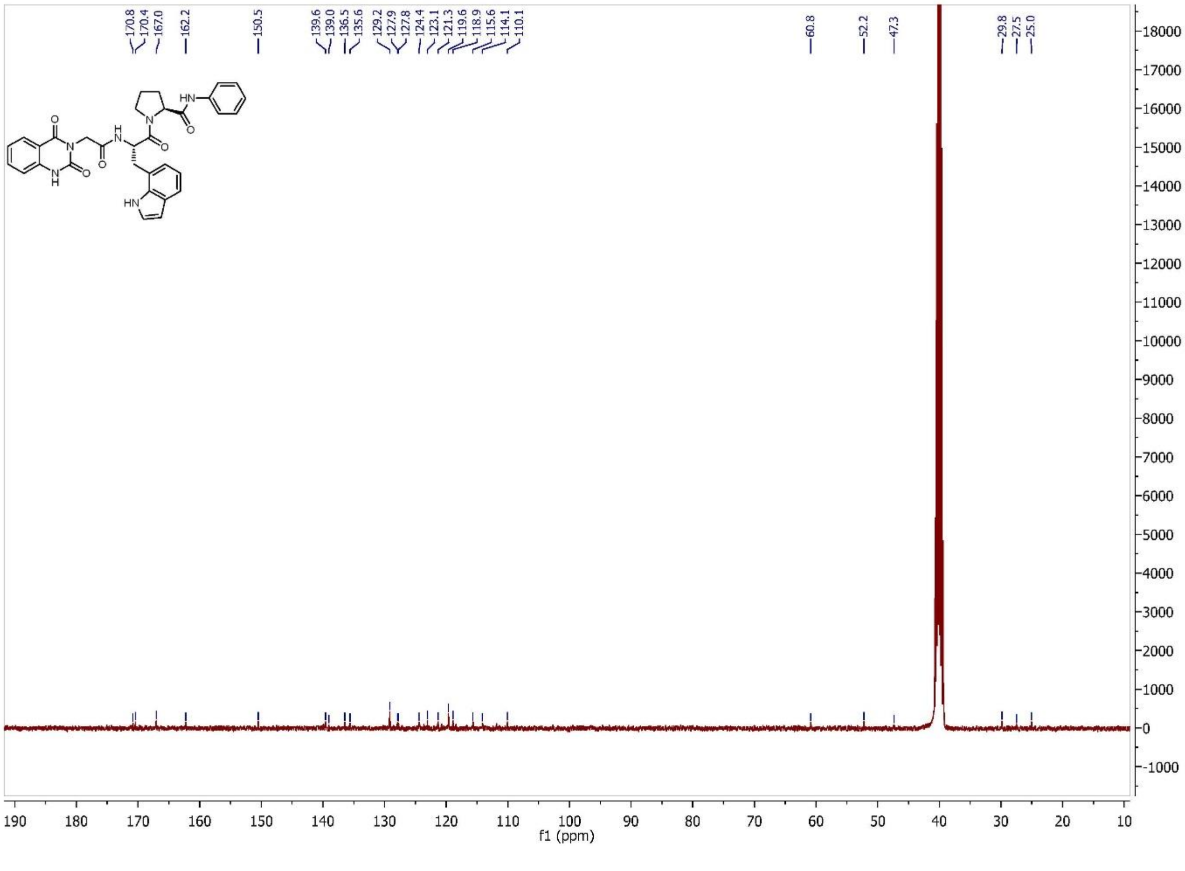

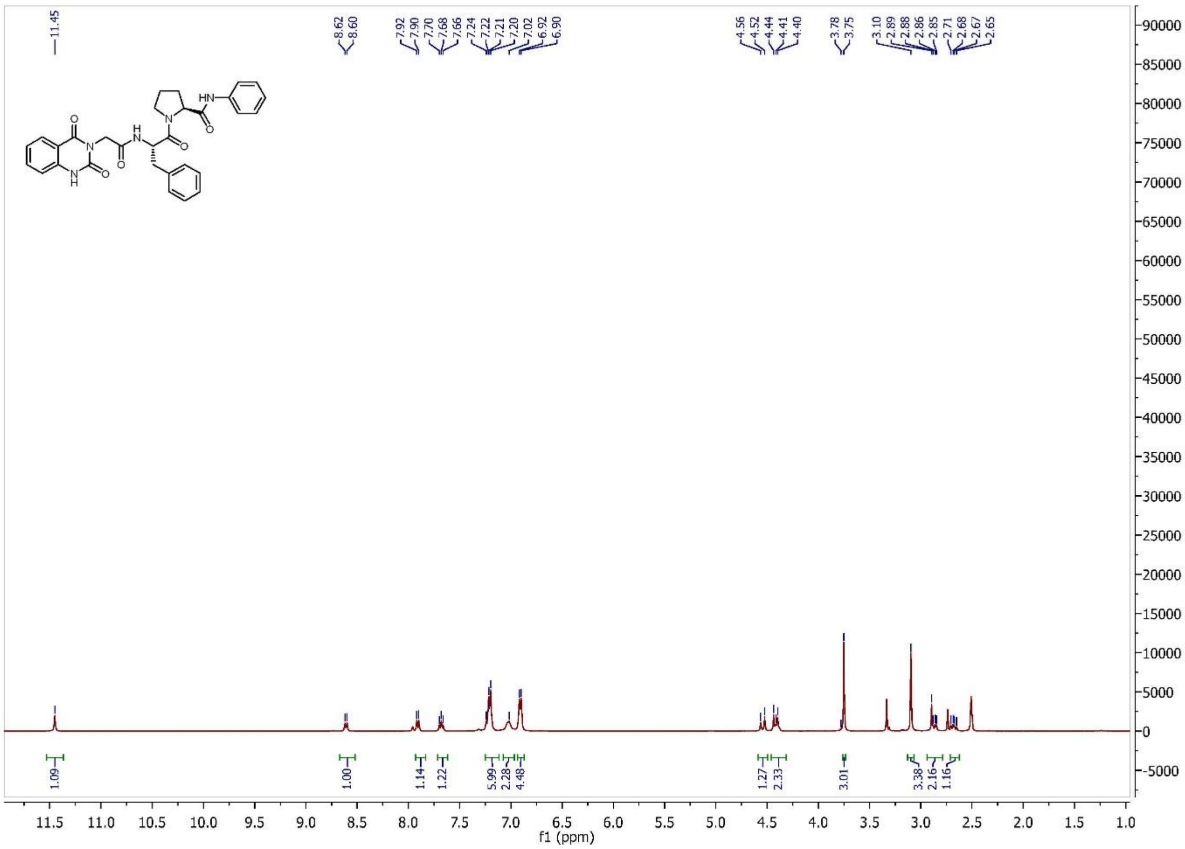

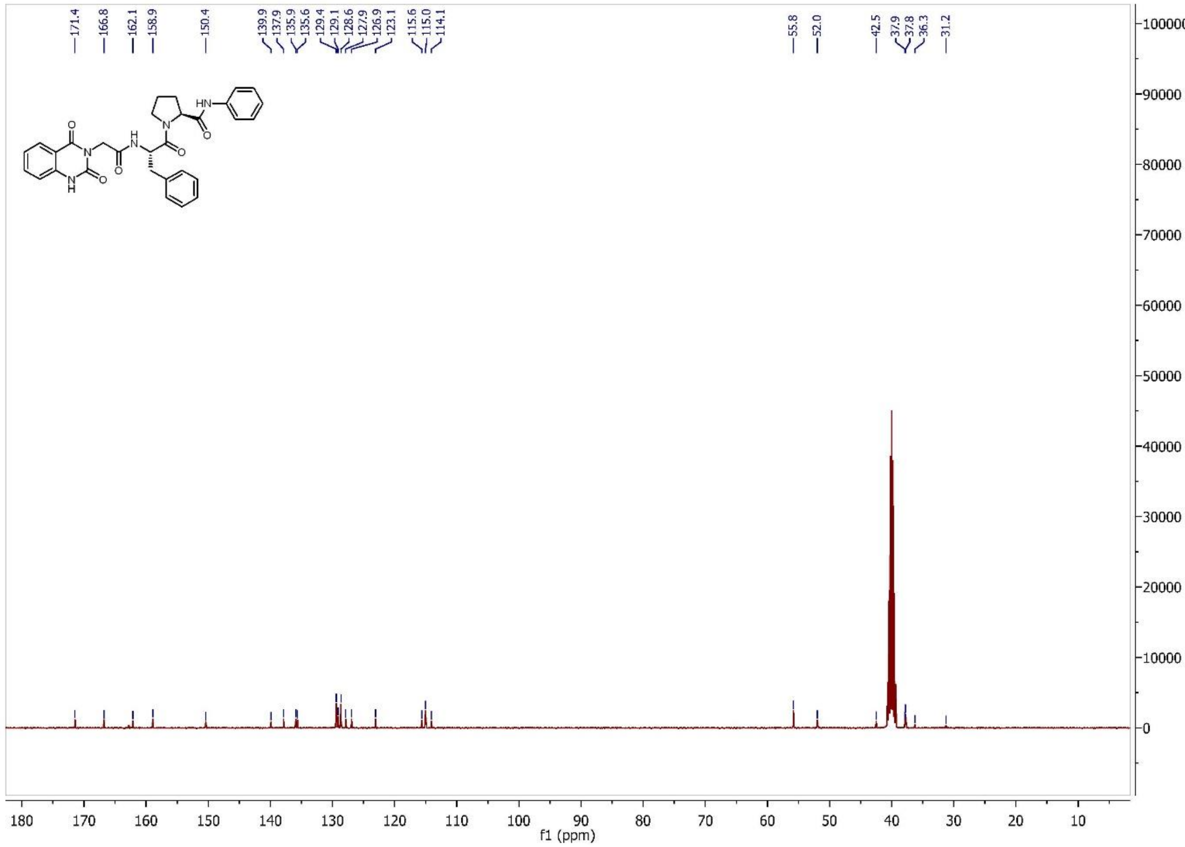

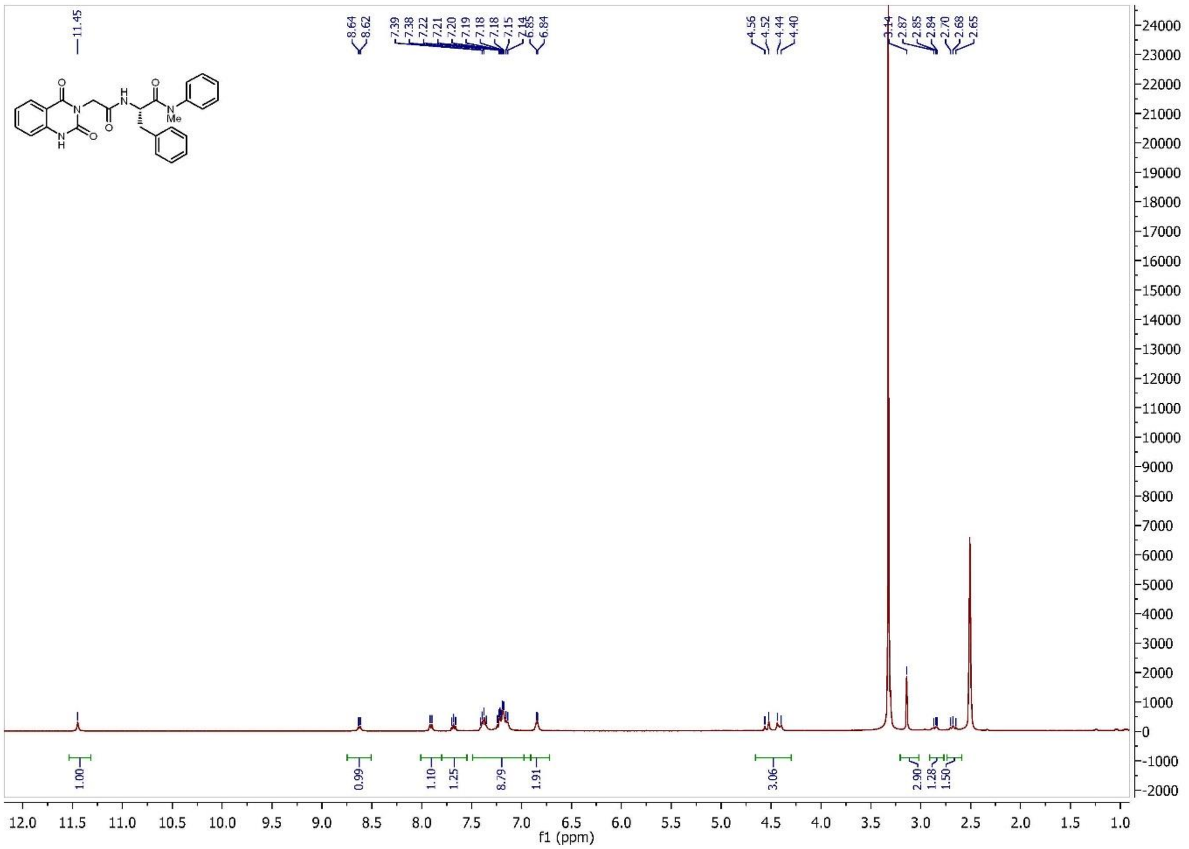

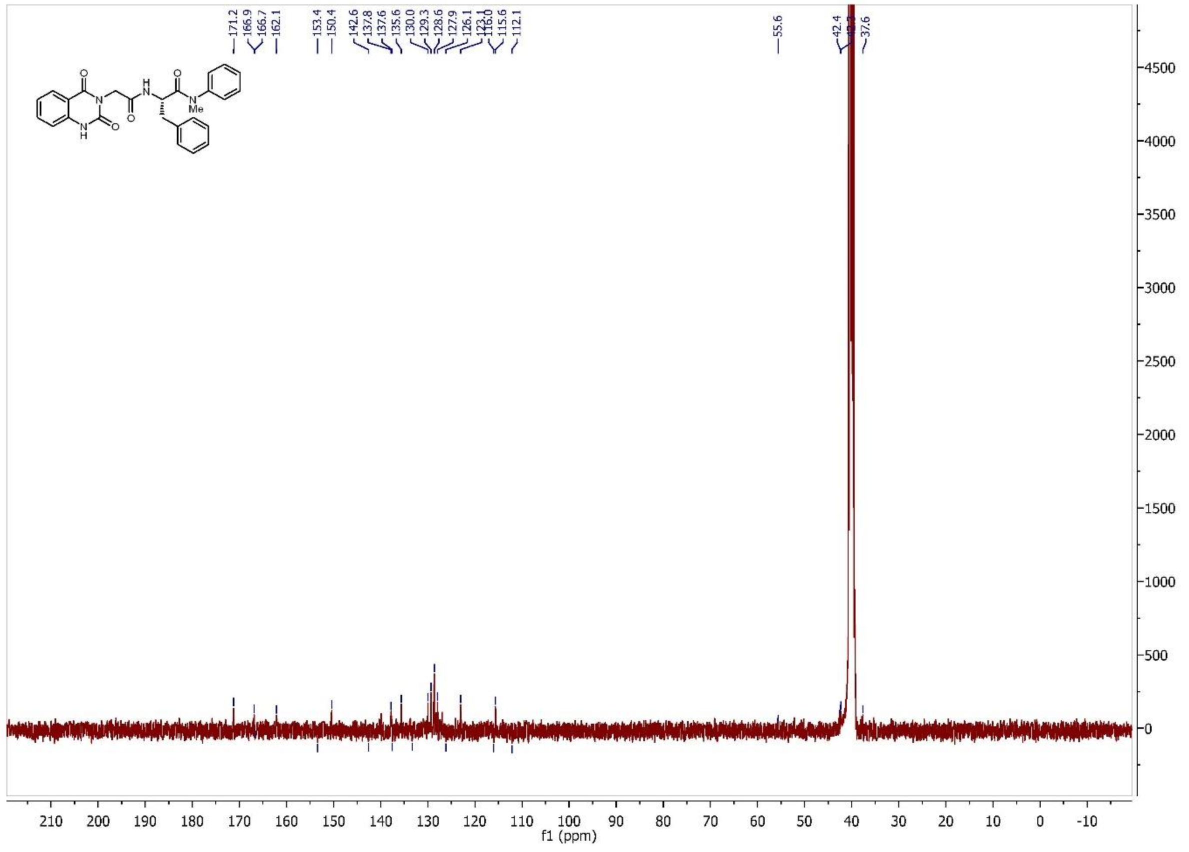

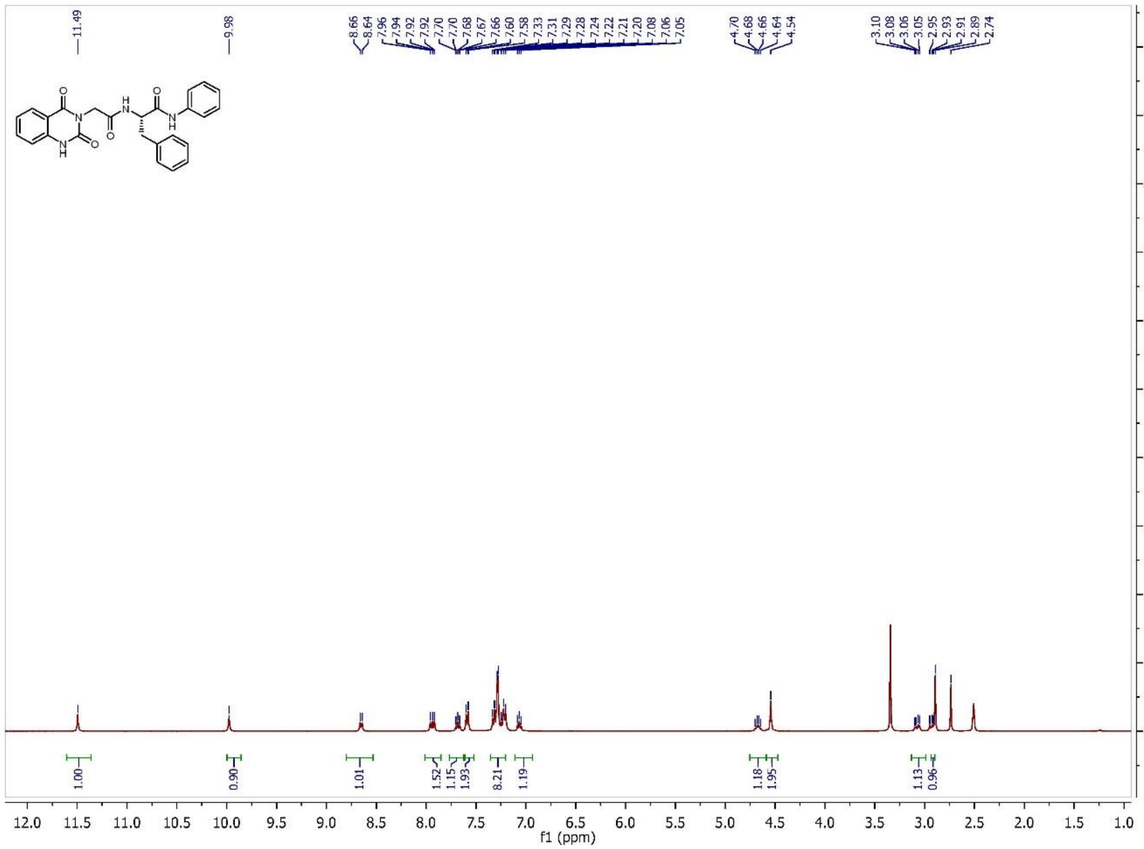

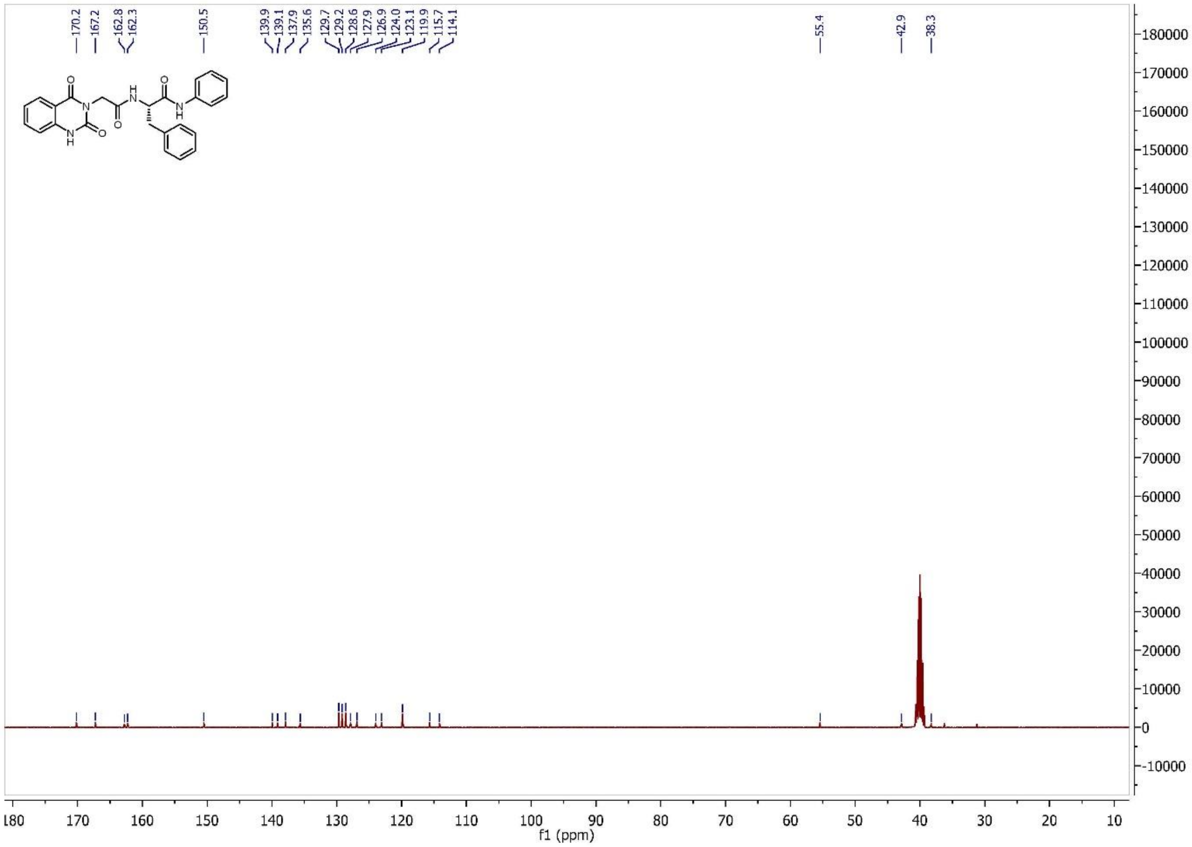

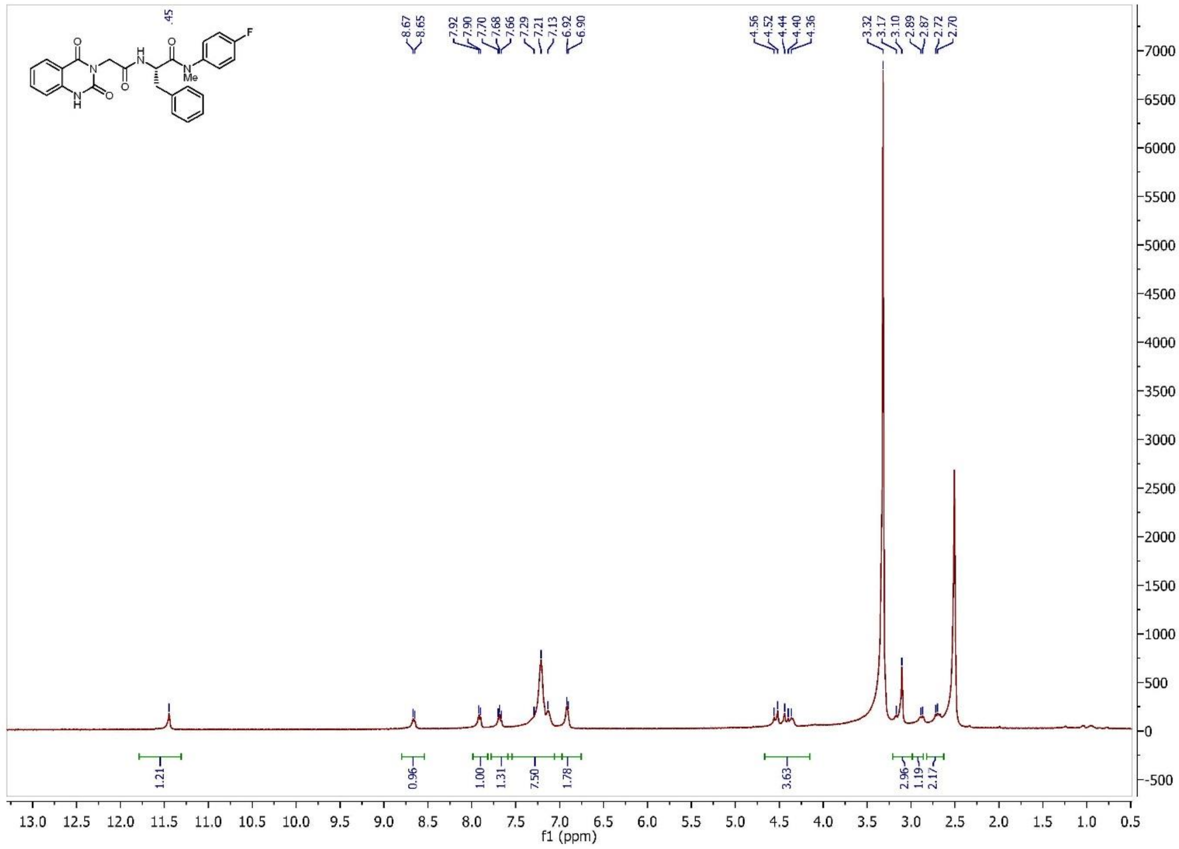

8.67
8.65
7.92
7.90
7.70
7.68
7.66
7.29
7.21
7.13
6.92
6.90
4.56
4.52
4.44
4.40
4.36
3.32
3.17
3.10
2.89
2.87
2.72
2.70
1.21
0.96
1.00
1.31
7.50
1.78
3.63
2.96
1.19
2.17
F
Me
f1 (ppm)

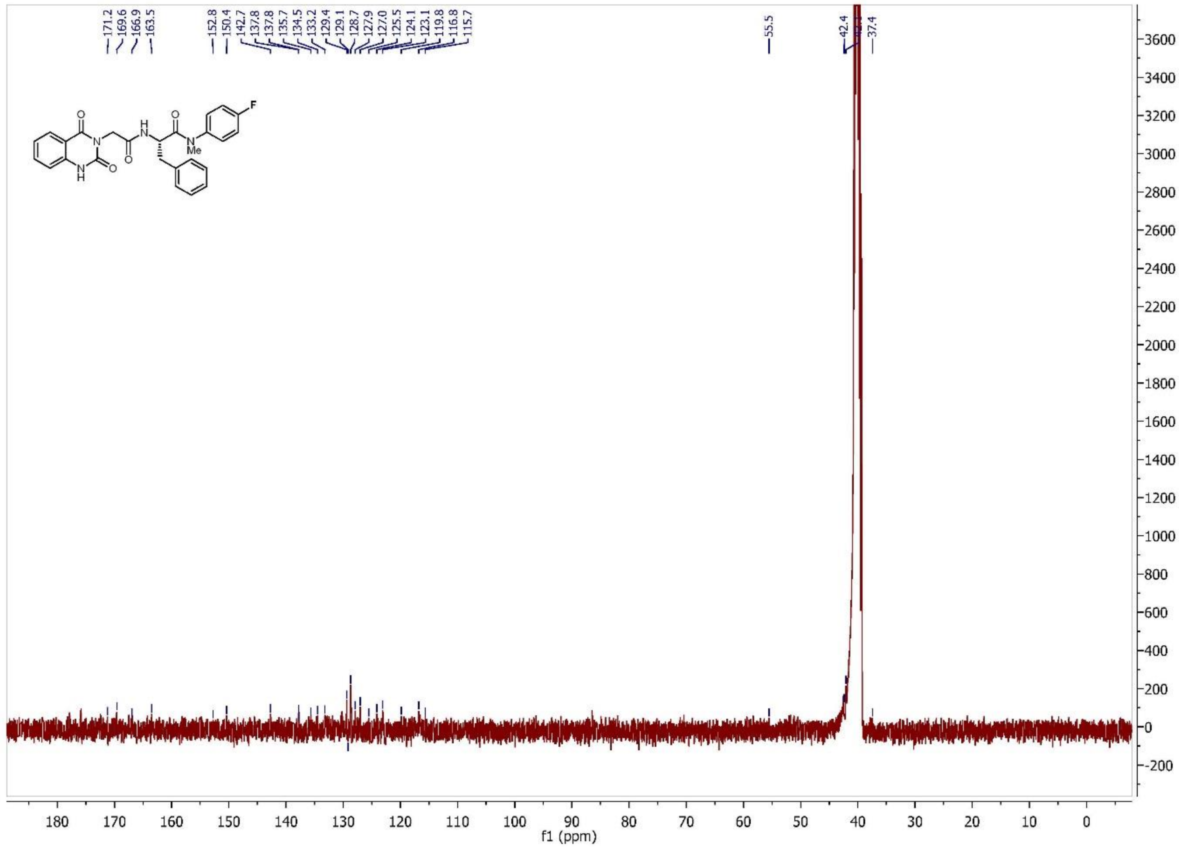

171.2
169.6
166.9
163.5
152.8
150.4
142.7
137.8
137.8
135.7
134.5
133.2
129.4
129.1
128.7
127.9
127.0
125.5
124.1
123.1
119.8
116.8
115.7
55.5
42.4
37.4
F
Me
f1 (ppm)

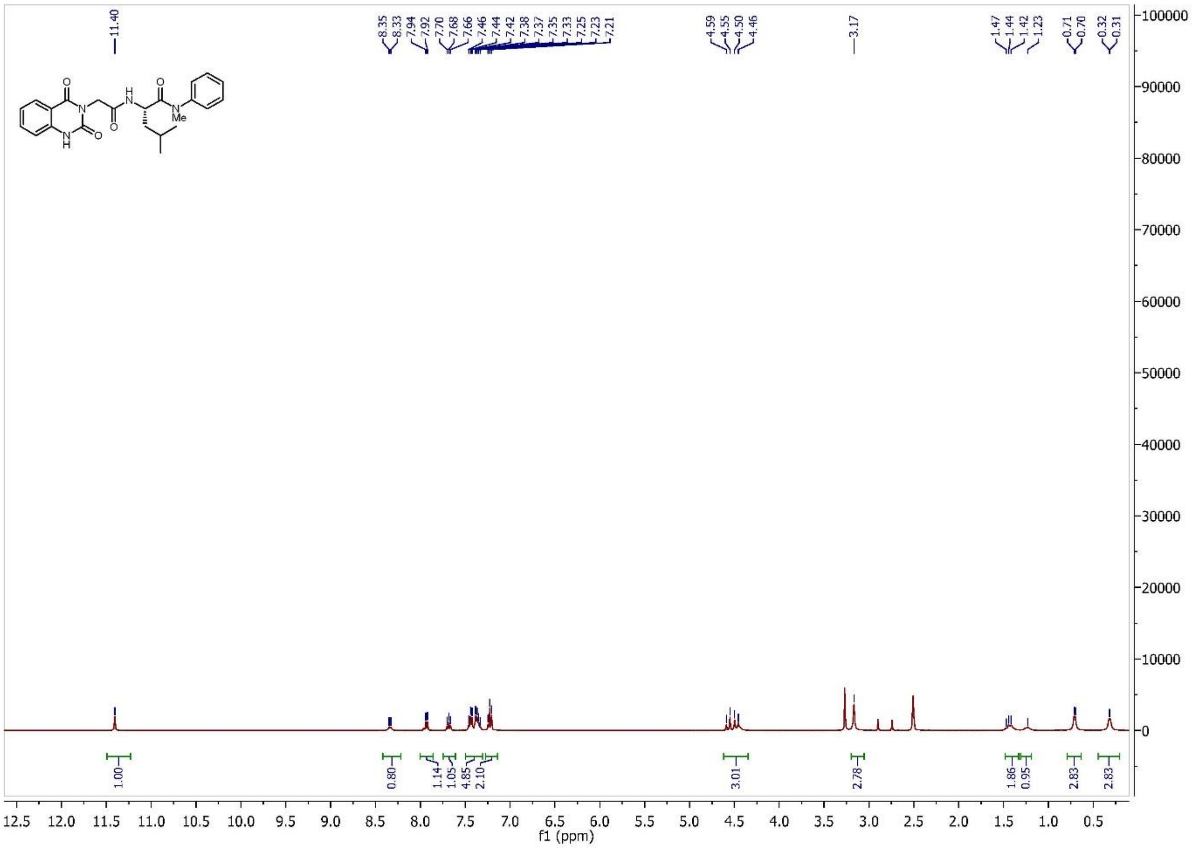
11.40
8.35
8.33
7.94
7.92
7.70
7.68
7.66
7.46
7.44
7.42
7.38
7.37
7.35
7.33
7.25
7.23
7.21
4.59
4.55
4.50
4.46
3.17
1.47
1.44
1.42
1.23
0.71
0.70
0.32
0.31
1.00
0.80
1.14
1.05
4.85
2.10
3.01
2.78
1.86
0.95
2.83
2.83
f1 (ppm)
Me

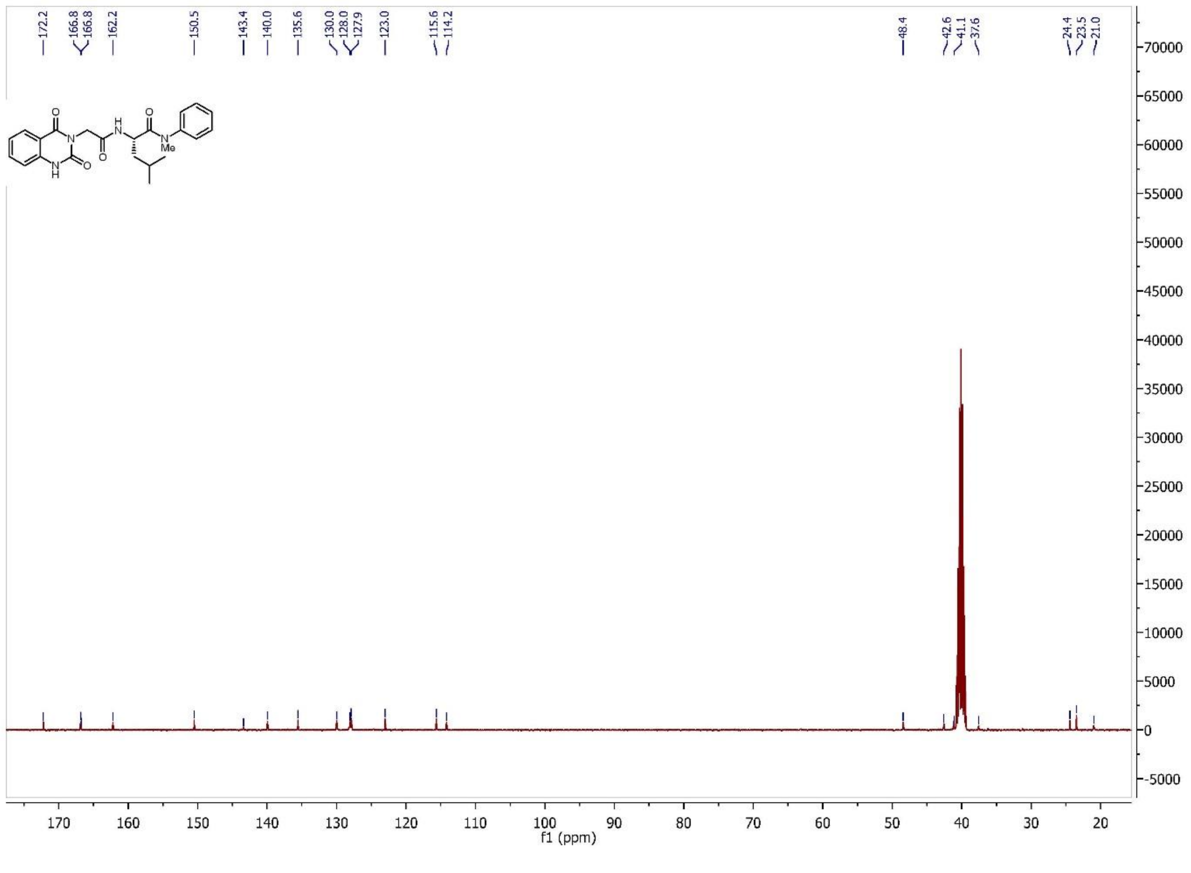
172.2
166.8
166.8
162.2
150.5
143.4
140.0
135.6
130.0
128.0
127.9
123.0
115.6
114.2
48.4
42.6
41.1
37.6
24.4
23.5
21.0
f1 (ppm)
Me

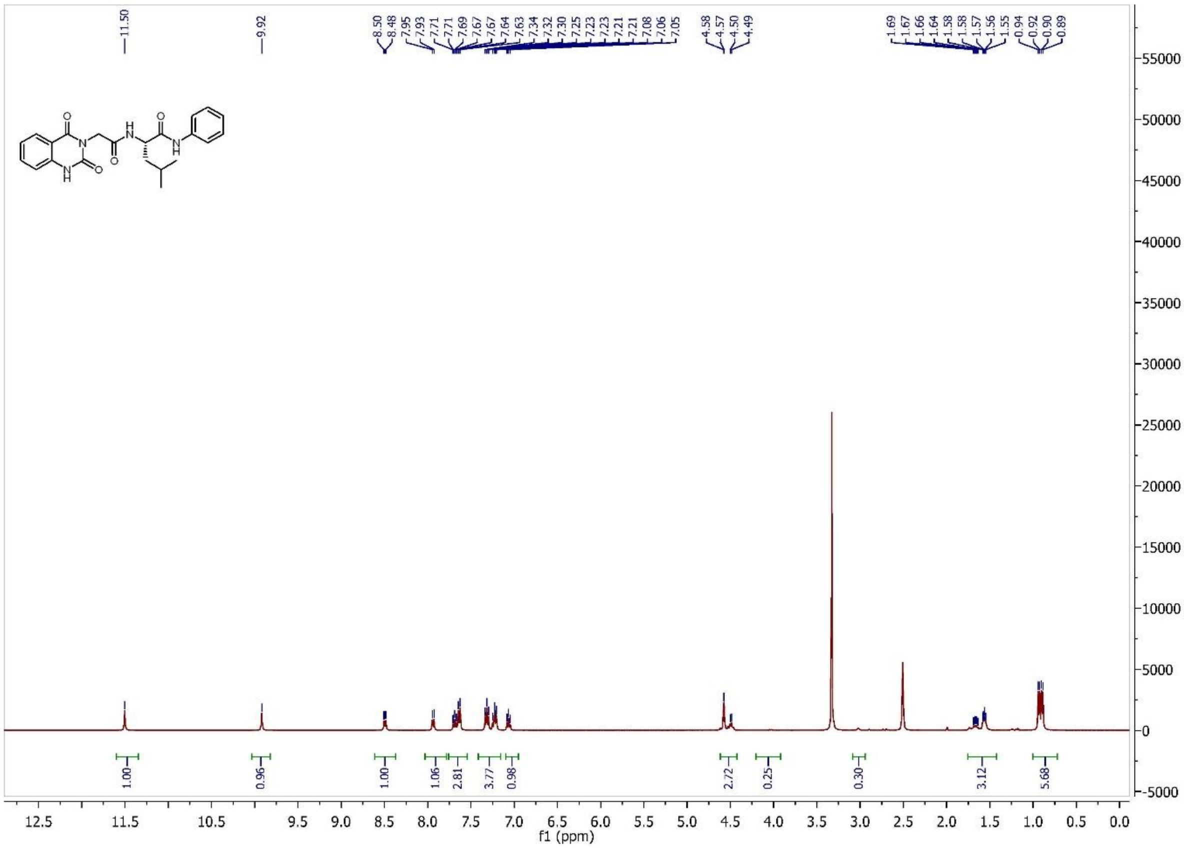
11.50
9.92
8.50
8.48
7.95
7.93
7.71
7.71
7.69
7.67
7.67
7.64
7.63
7.34
7.32
7.30
7.25
7.23
7.23
7.21
7.21
7.08
7.06
7.05
4.58
4.57
4.50
4.49
1.69
1.67
1.66
1.64
1.58
1.58
1.57
1.56
1.55
0.94
0.92
0.90
0.89
1.00
0.96
1.00
1.06
2.81
3.77
0.98
2.72
0.25
0.30
3.12
5.68
f1 (ppm)

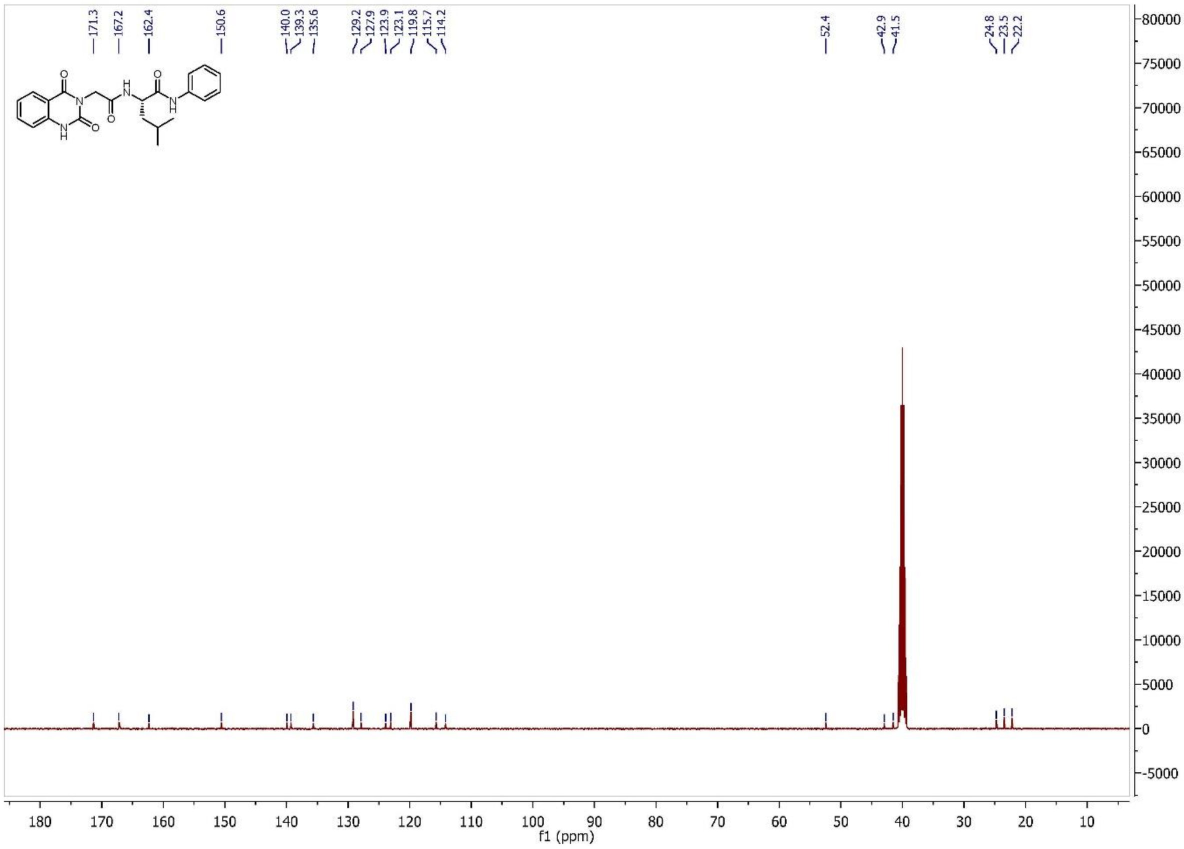
171.3
167.2
162.4
150.6
140.0
139.3
135.6
129.2
127.9
123.9
123.1
119.8
115.7
114.2
52.4
42.9
41.5
24.8
23.5
22.2
f1 (ppm)

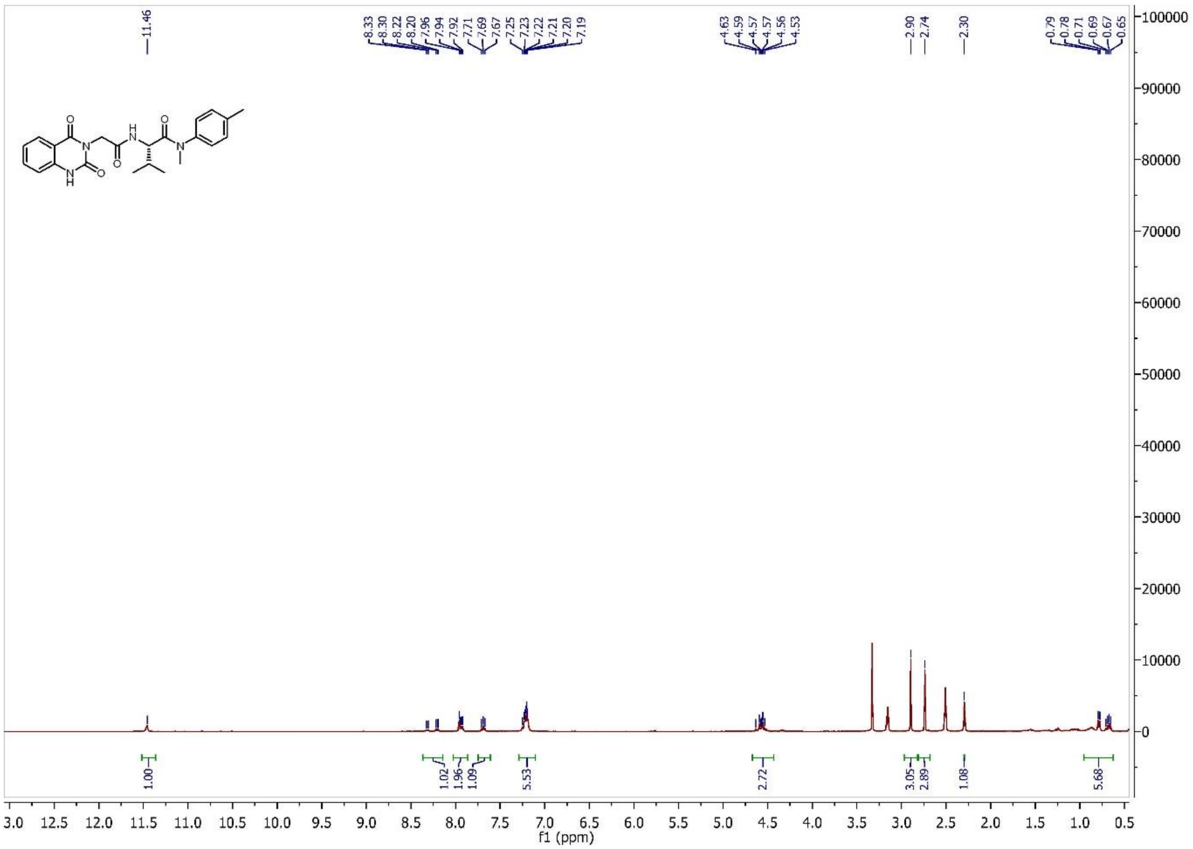

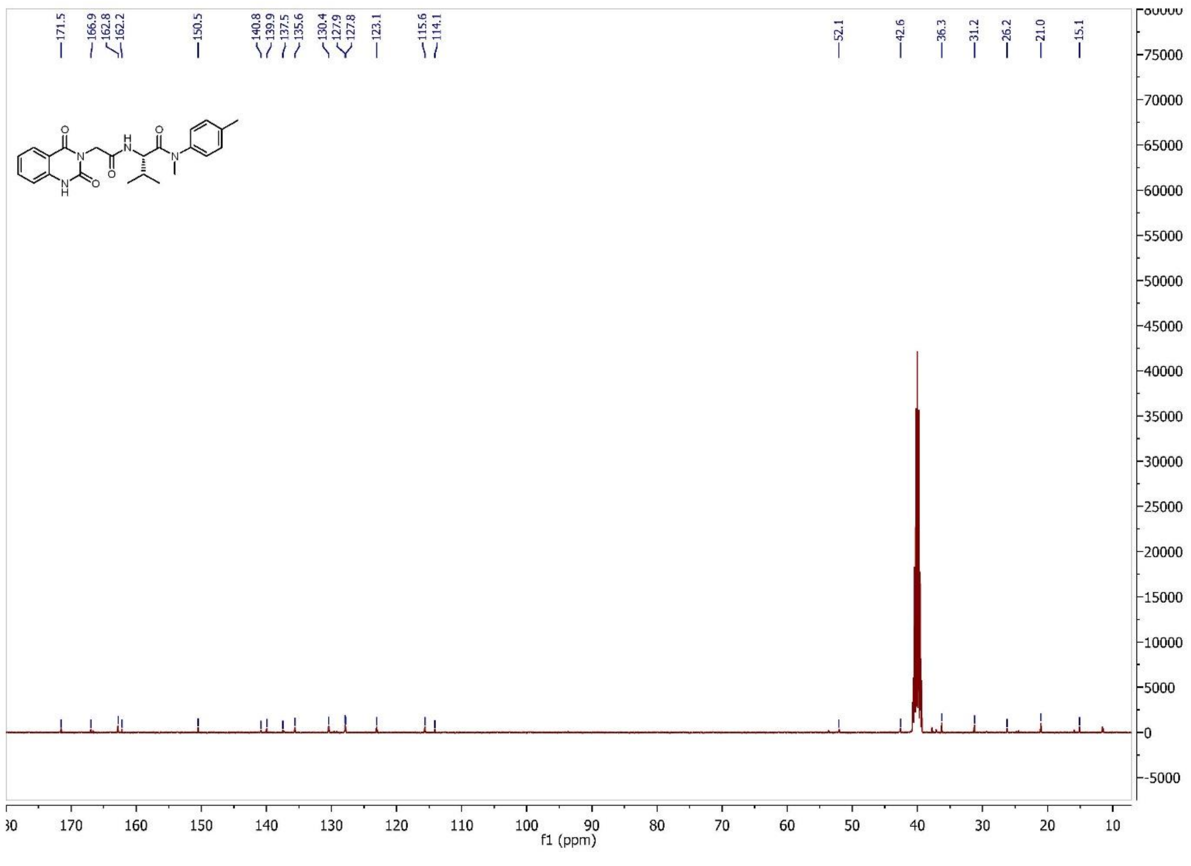

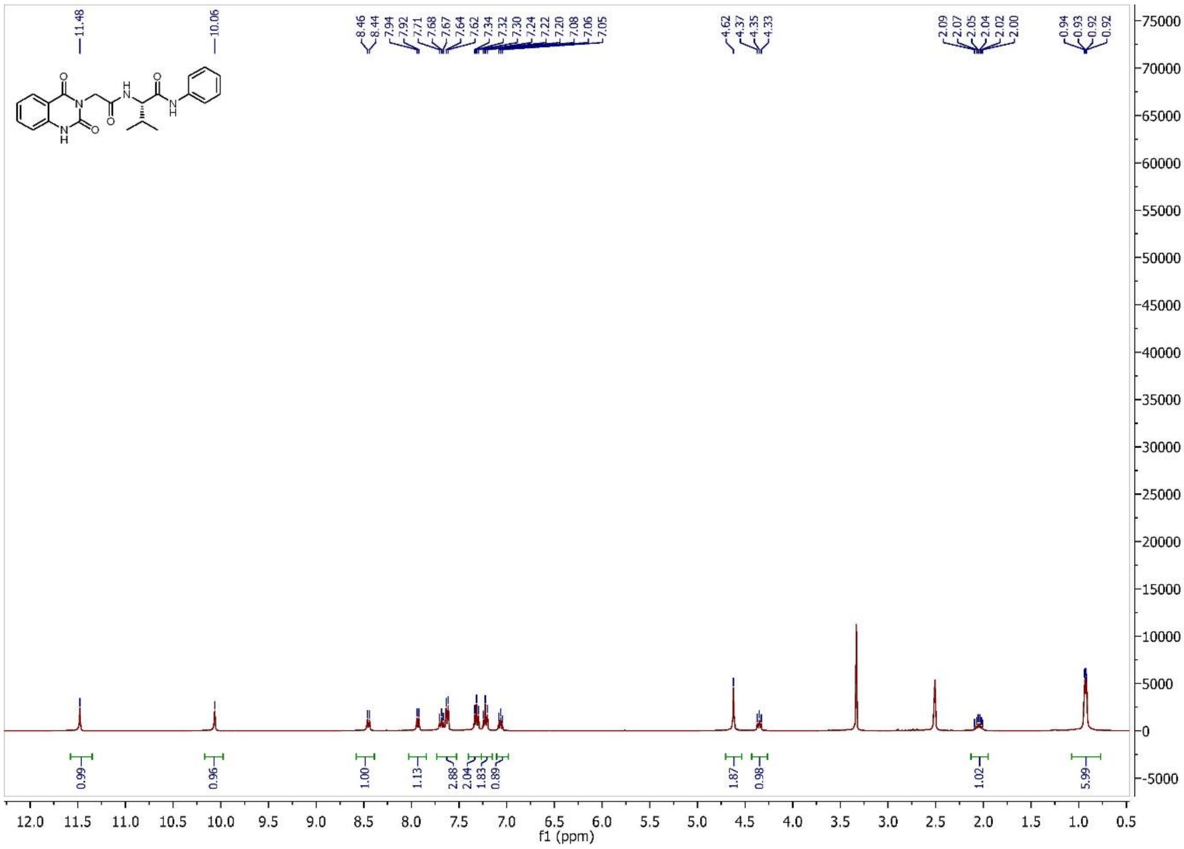

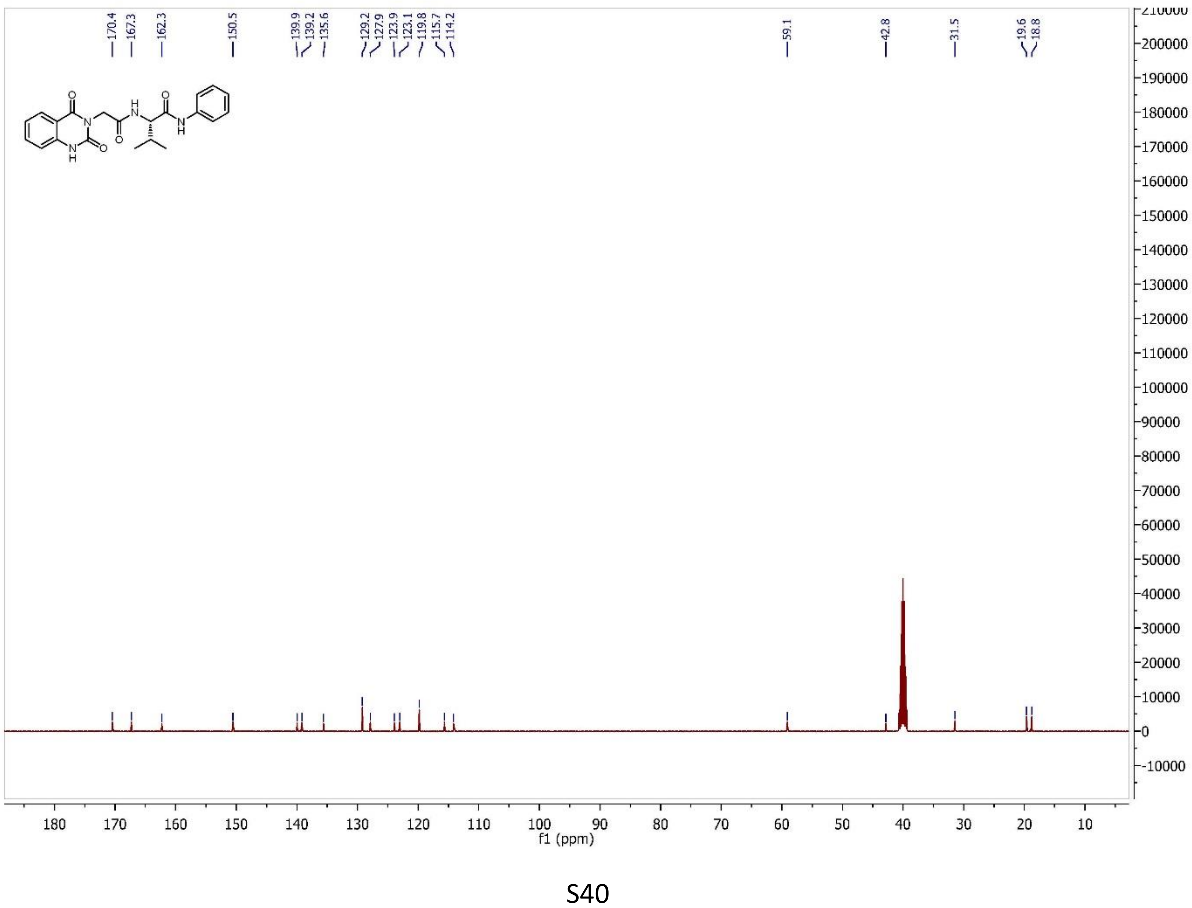

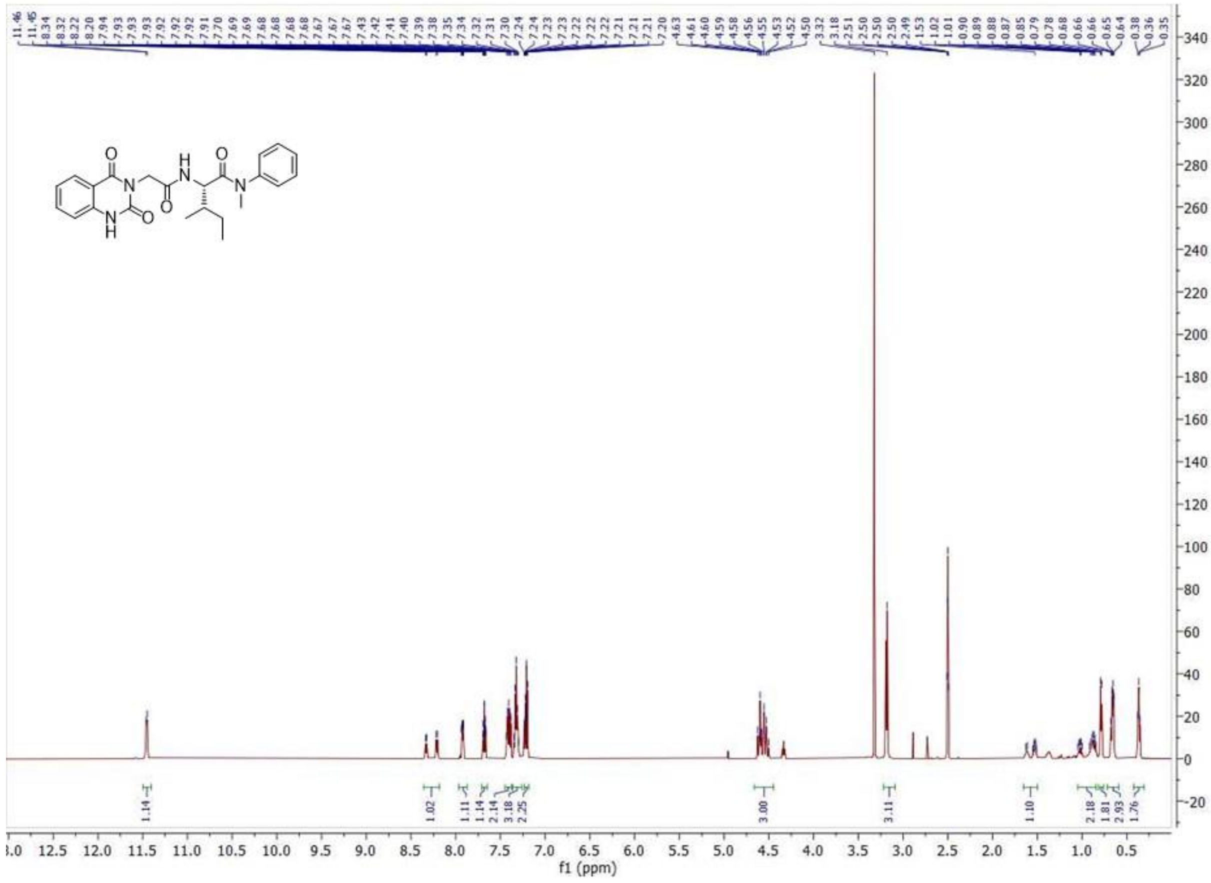

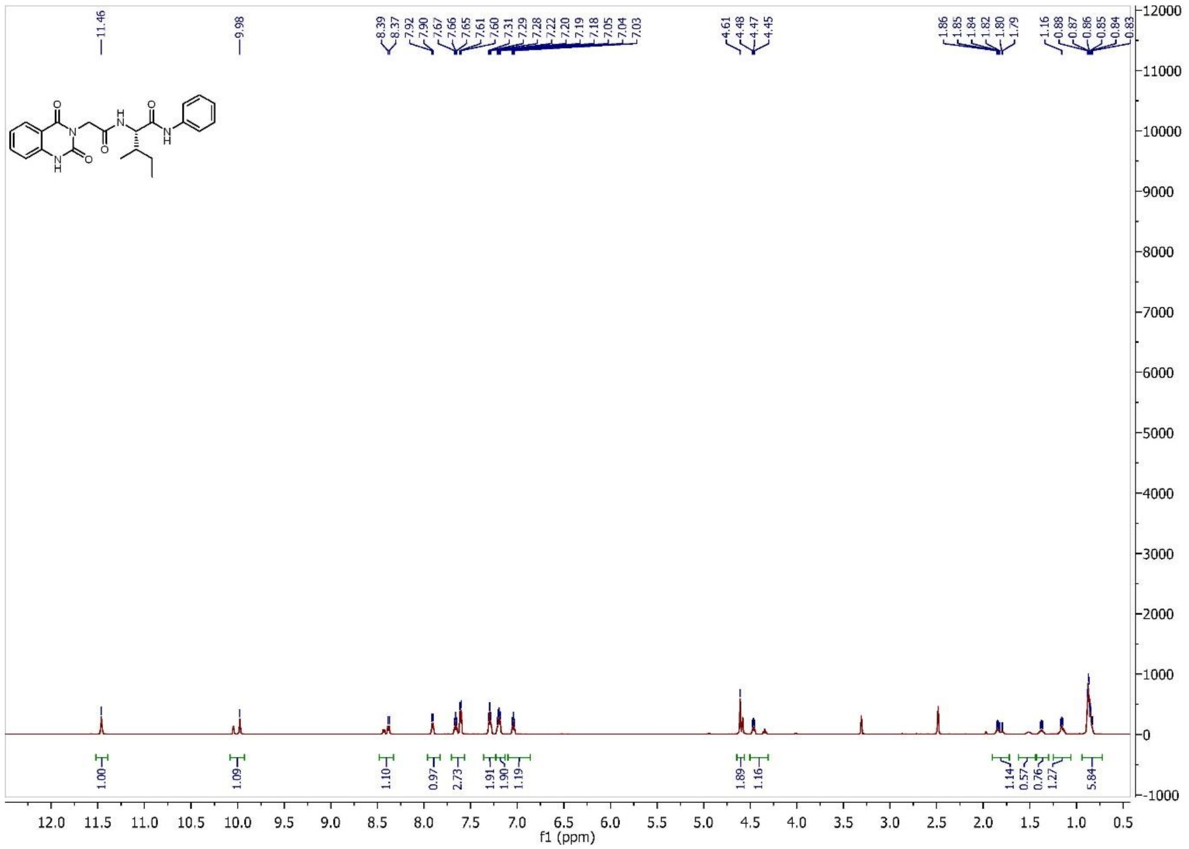

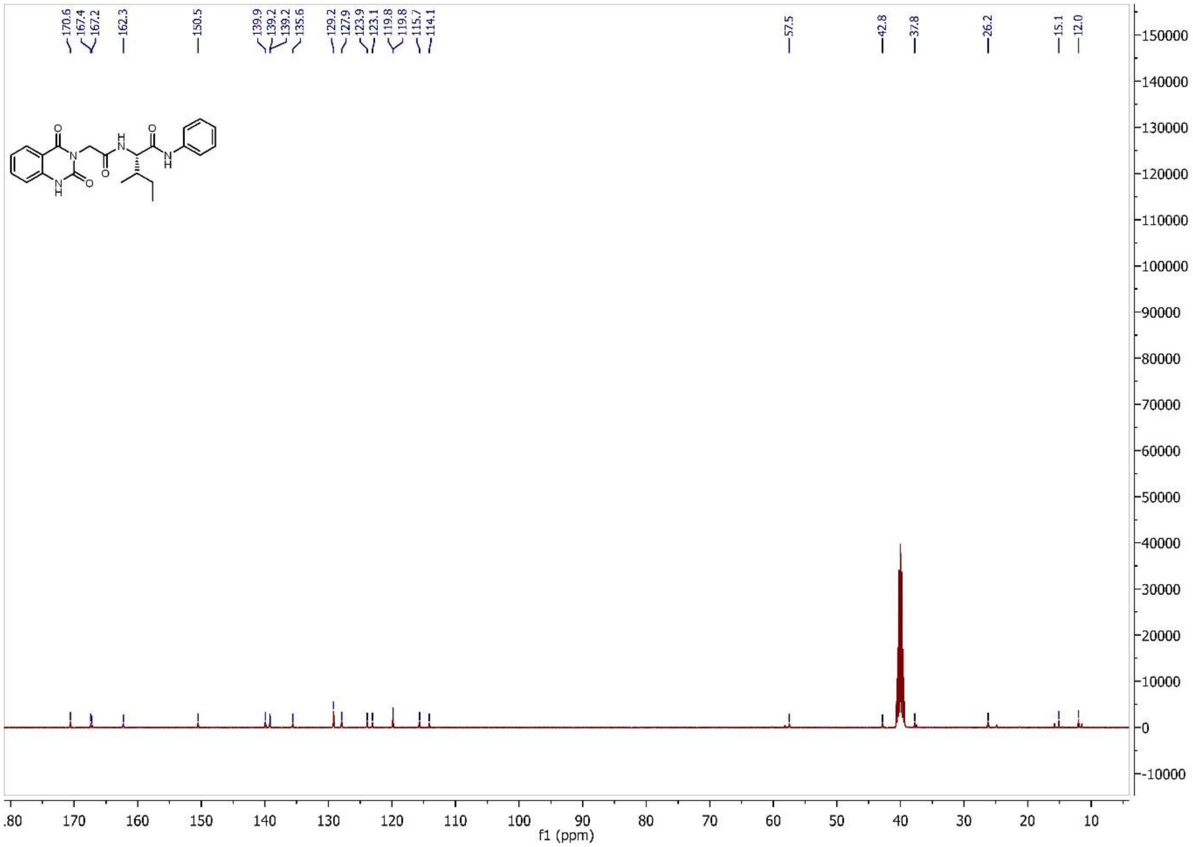

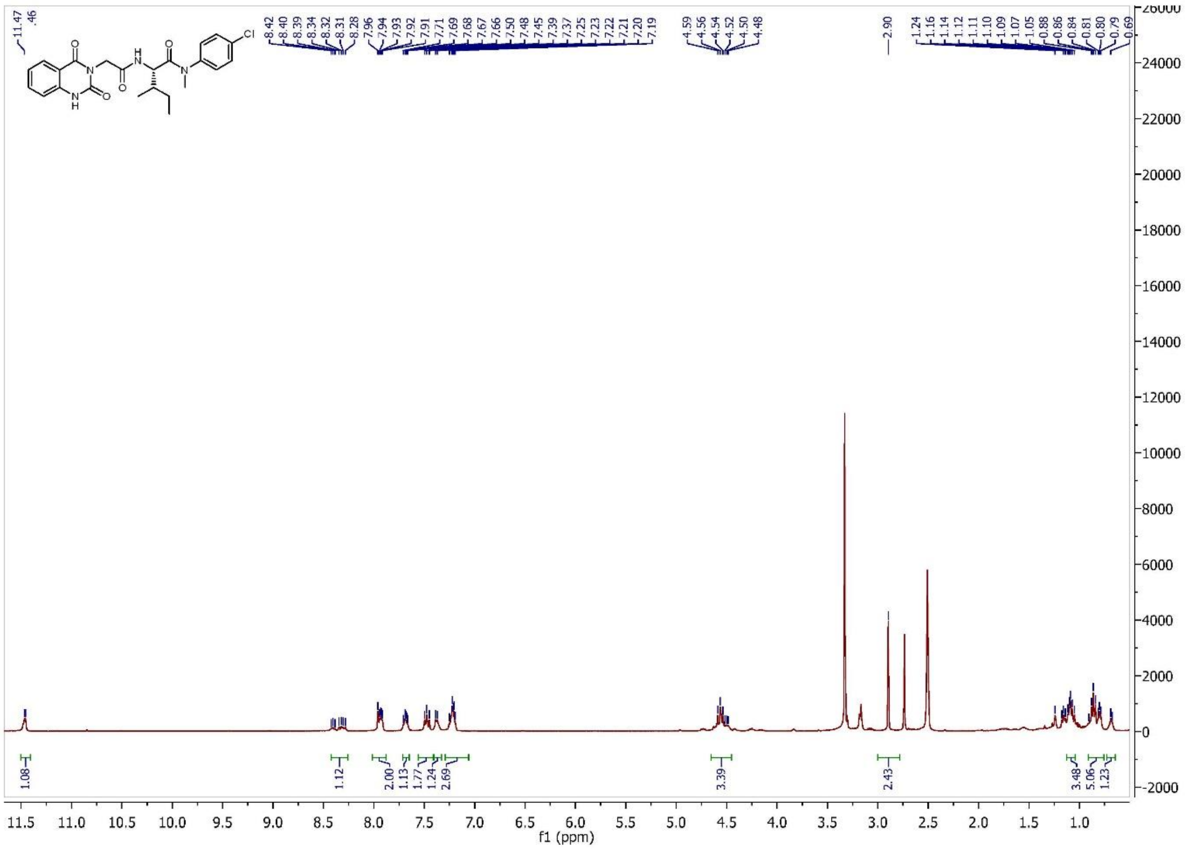

11.47
.46
8.42 8.40 8.39 8.34 8.32 8.31 8.28 7.96 7.94 7.93 7.92 7.91 7.71 7.69 7.68 7.67 7.66 7.50 7.48 7.45 7.39 7.37 7.25 7.23 7.22 7.21 7.20 7.19
4.59 4.56 4.54 4.52 4.50 4.48
2.90
1.24 1.16 1.14 1.12 1.11 1.10 1.09 1.07 1.05 0.88 0.86 0.84 0.81 0.80 0.79 0.69
1.08 1.12 2.00 1.13 1.77 1.24 2.69 3.39 2.43 3.48 5.06 1.23
f1 (ppm)

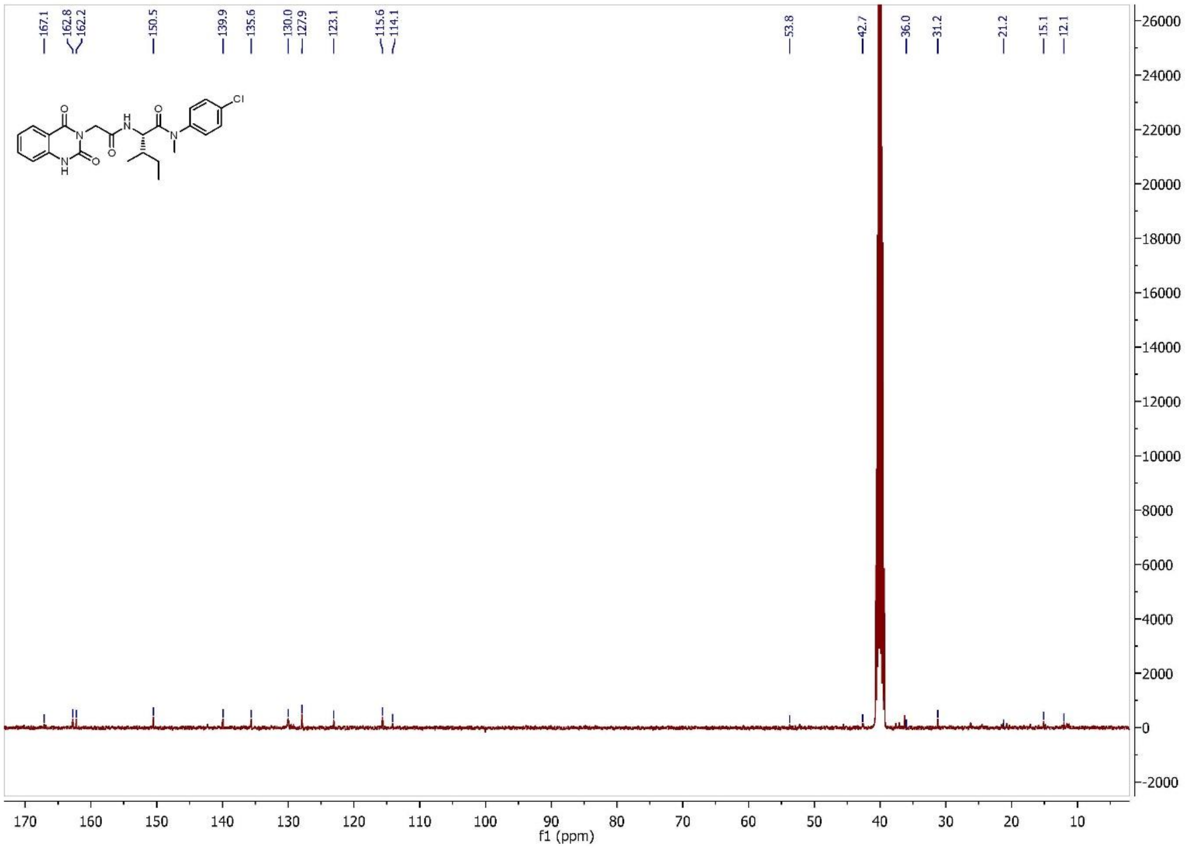

167.1 162.8 162.2 150.5 139.9 135.6 130.0 127.9 123.1 115.6 114.1 53.8 42.7 36.0 31.2 21.2 15.1 12.1
f1 (ppm)

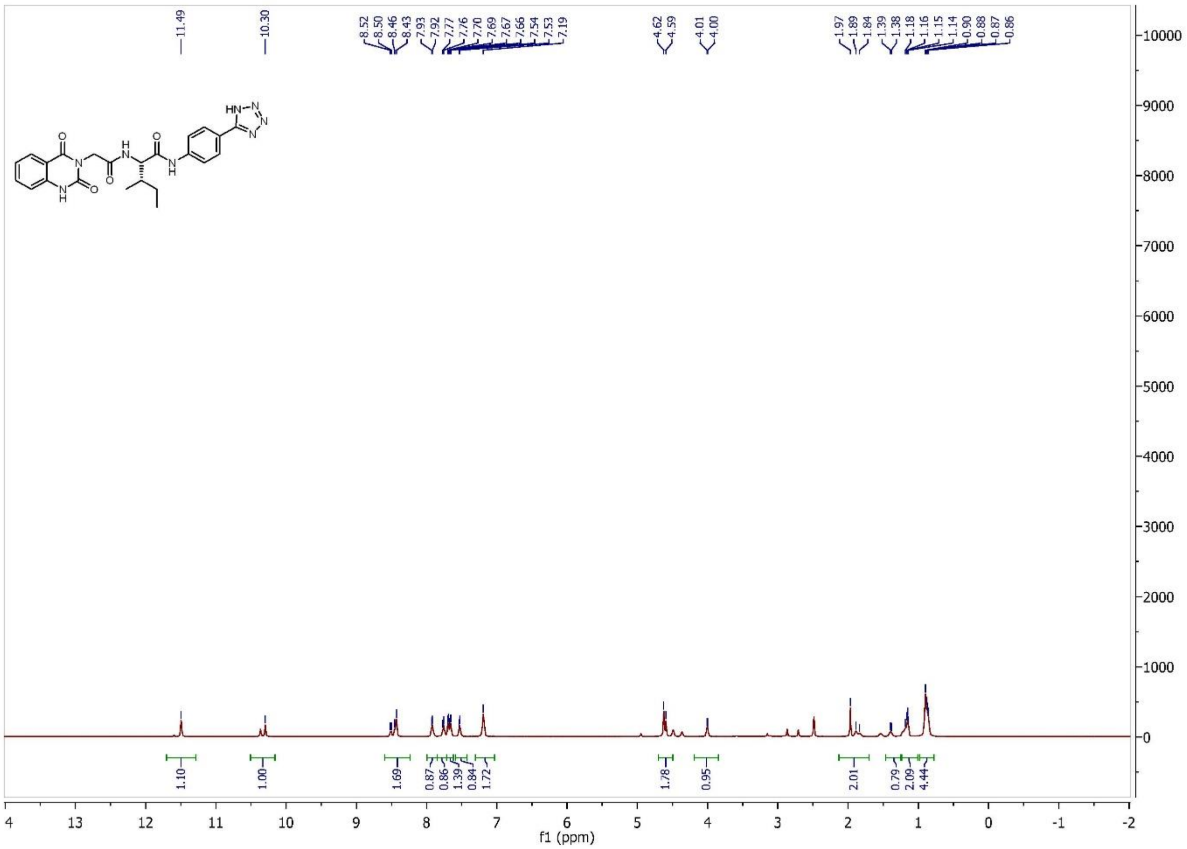

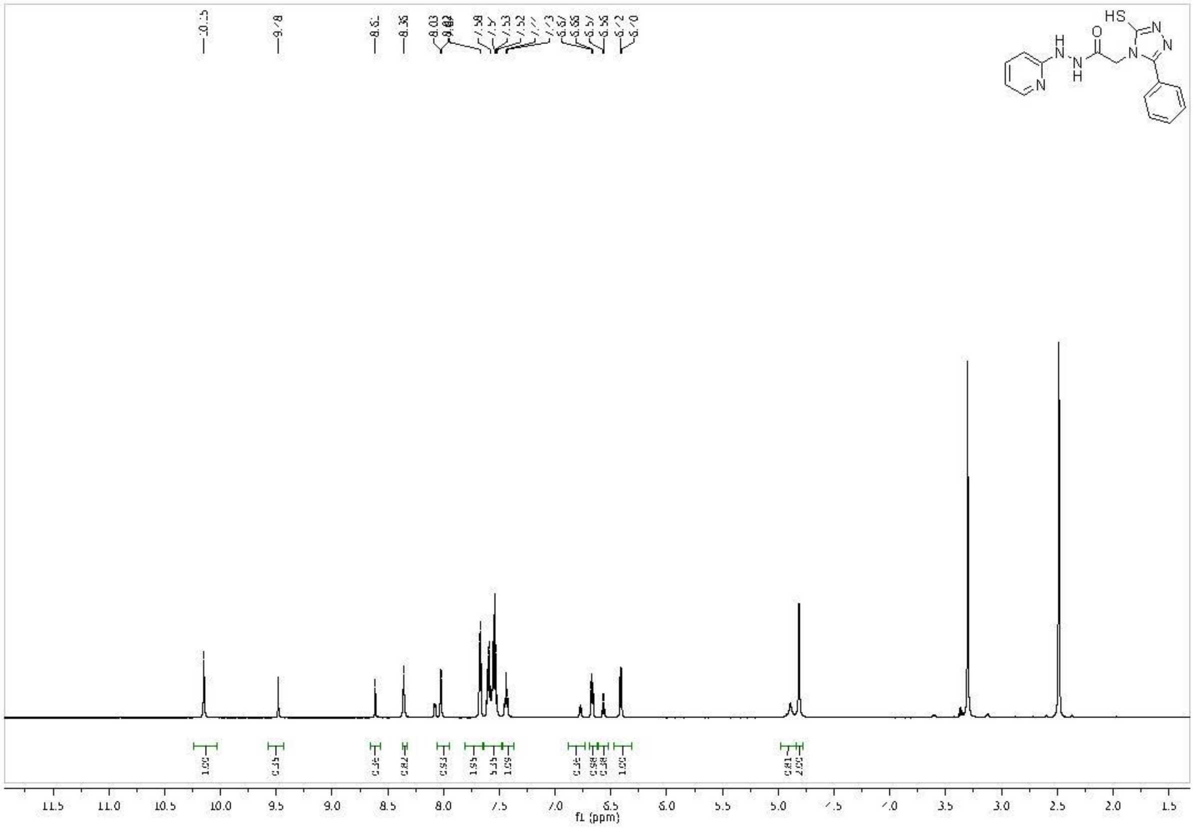
HS
N
N
N
H
N
N
H
O
f1 (ppm)

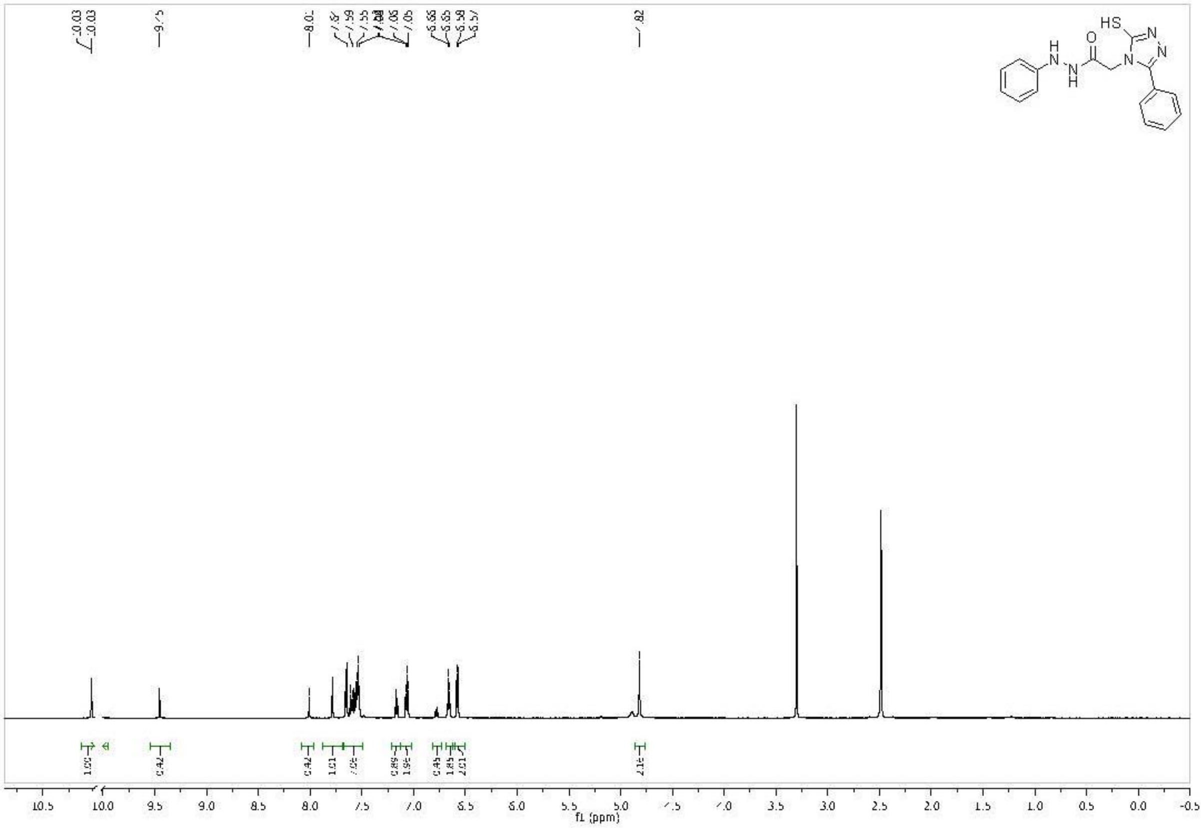
HS
N
N
N
H
N
N
H
O
f1 (ppm)

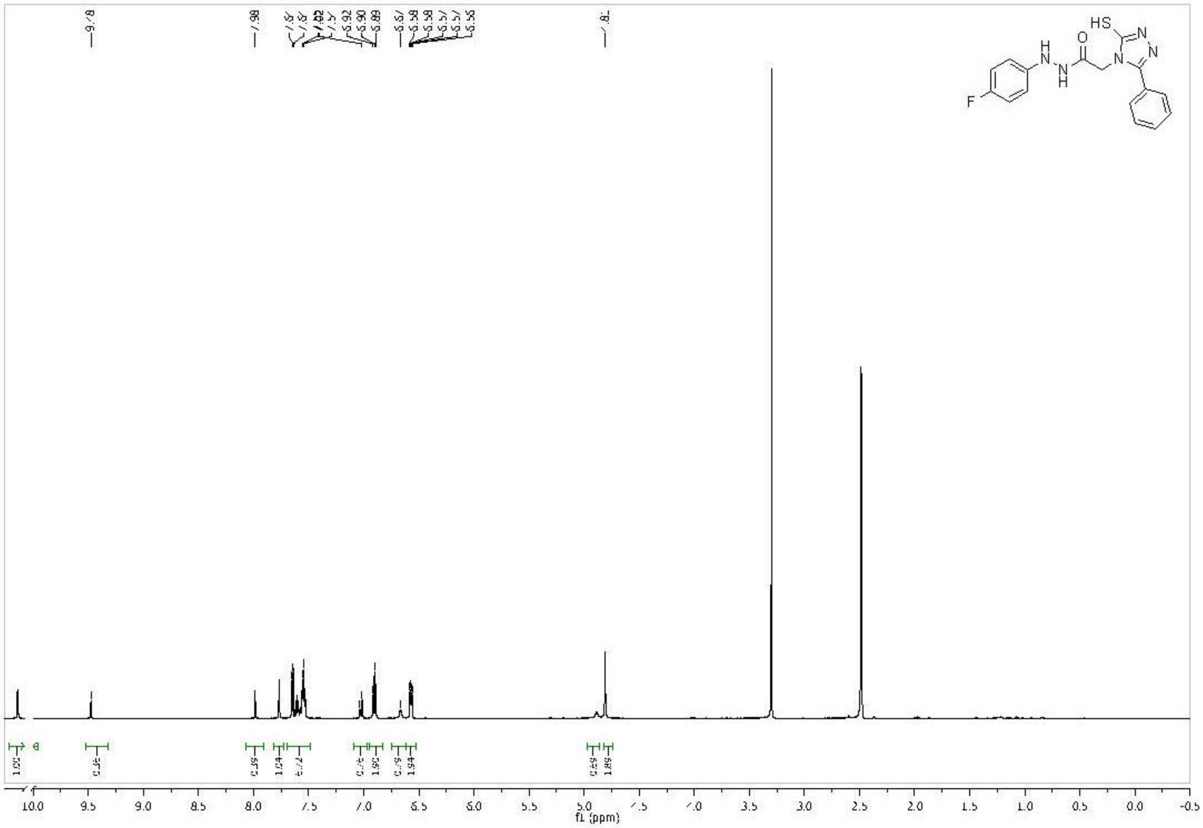

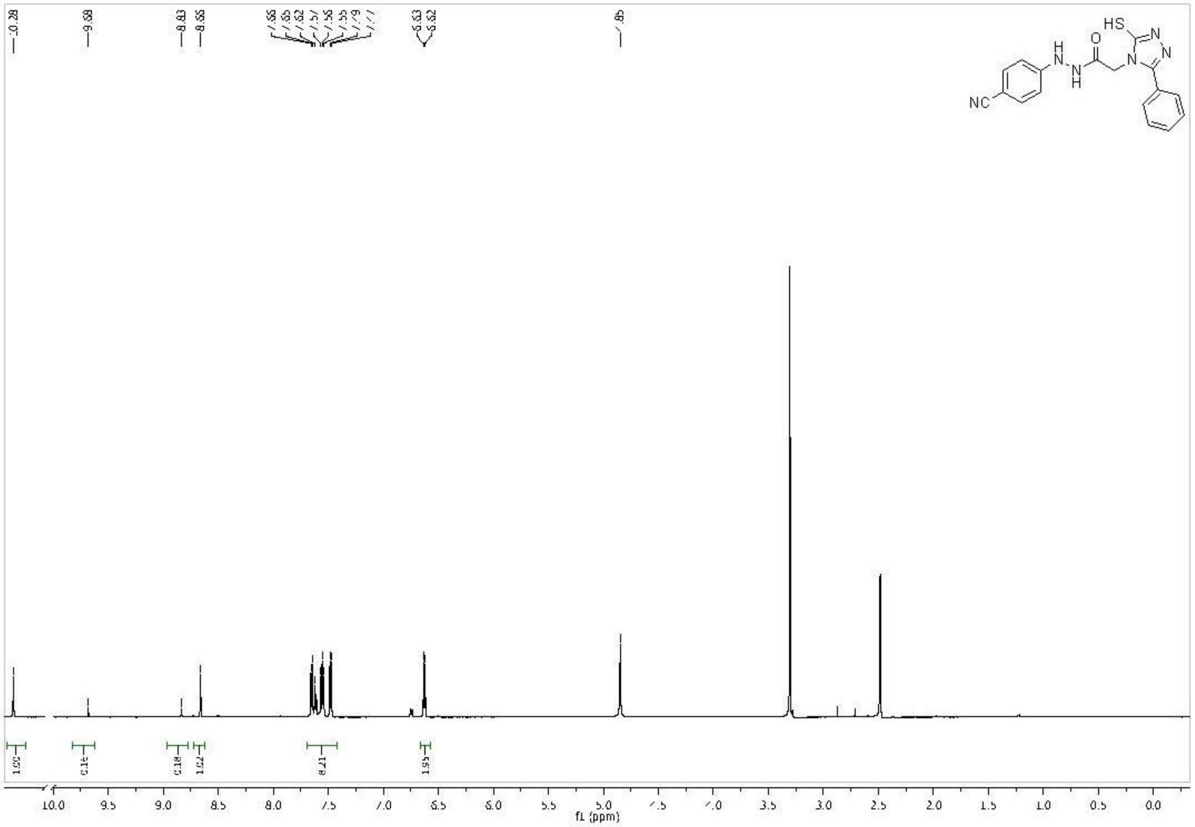

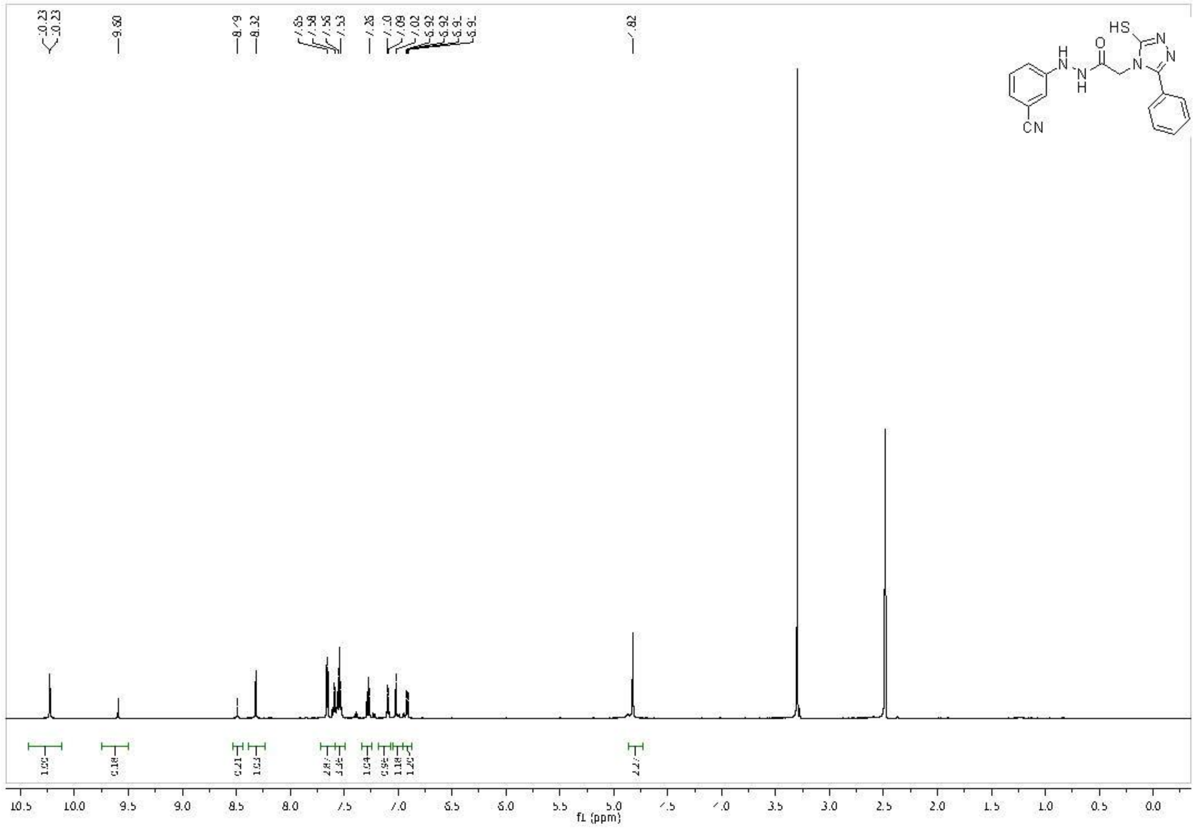

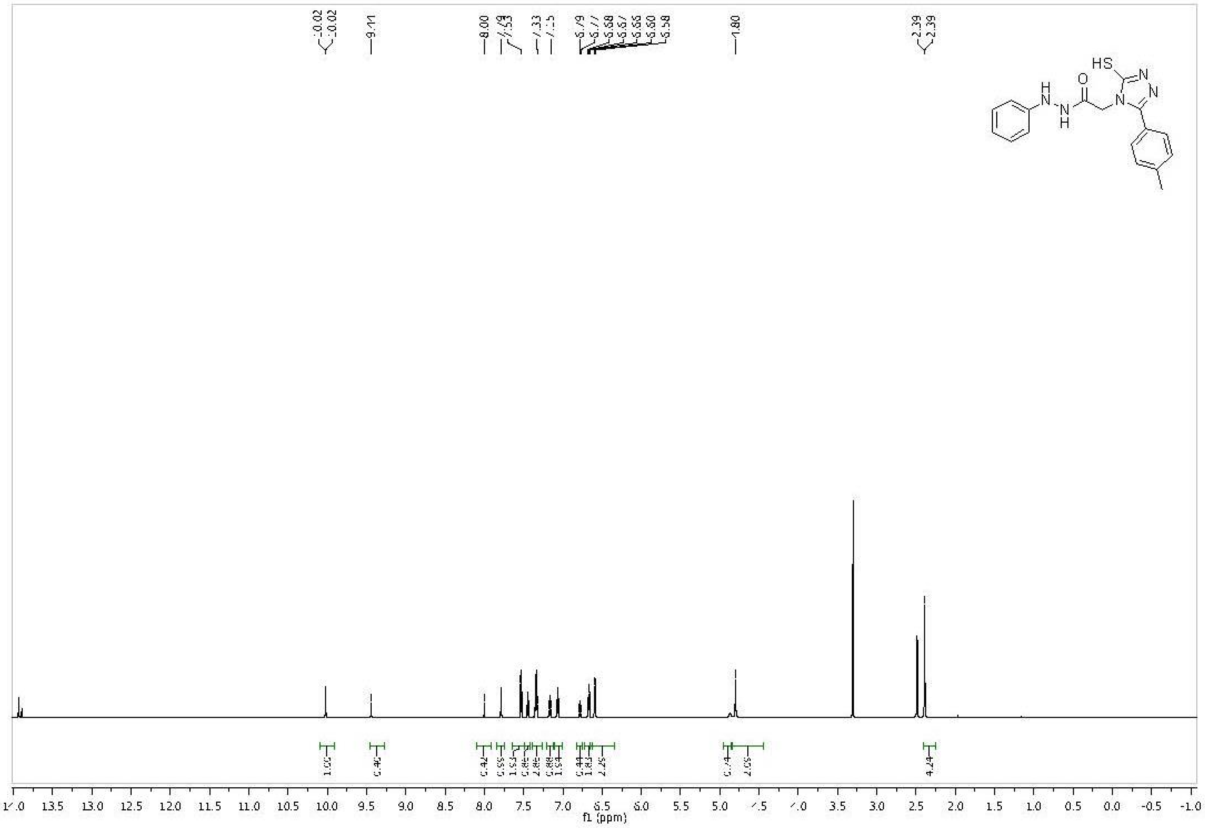

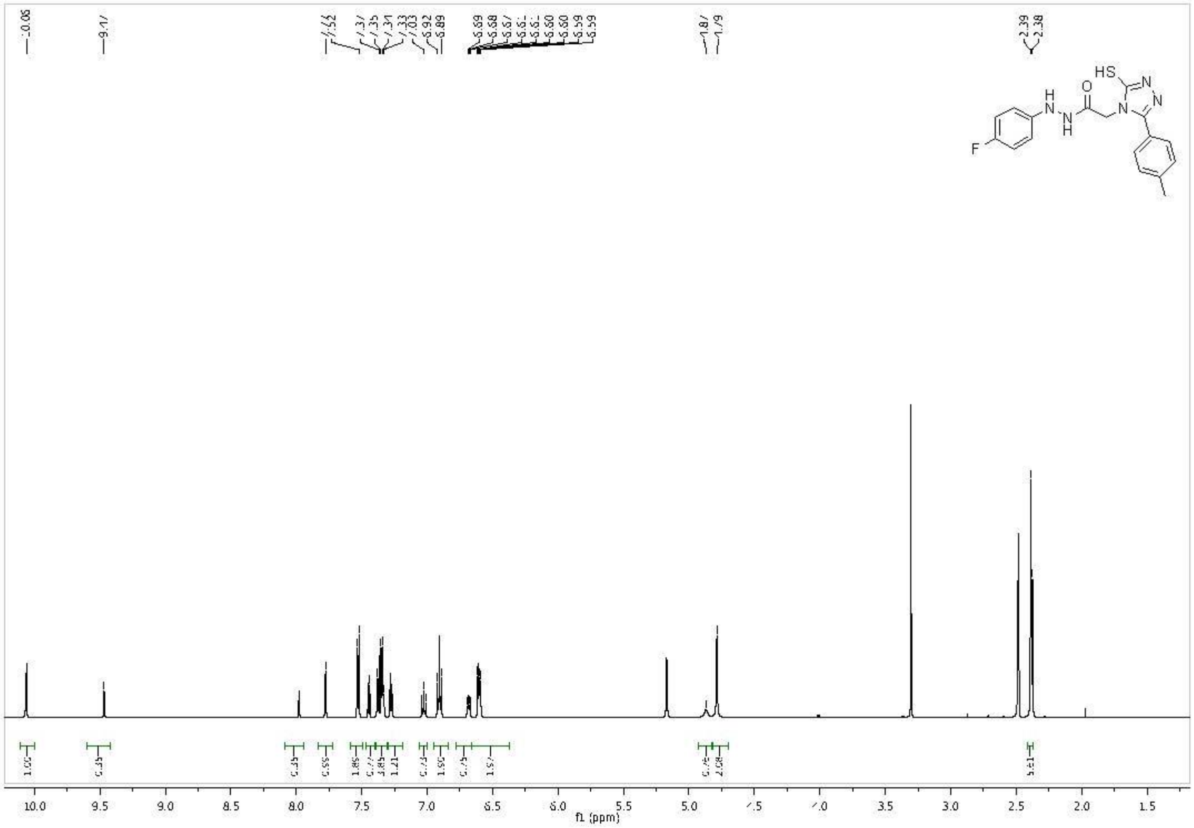
f1 (ppm)

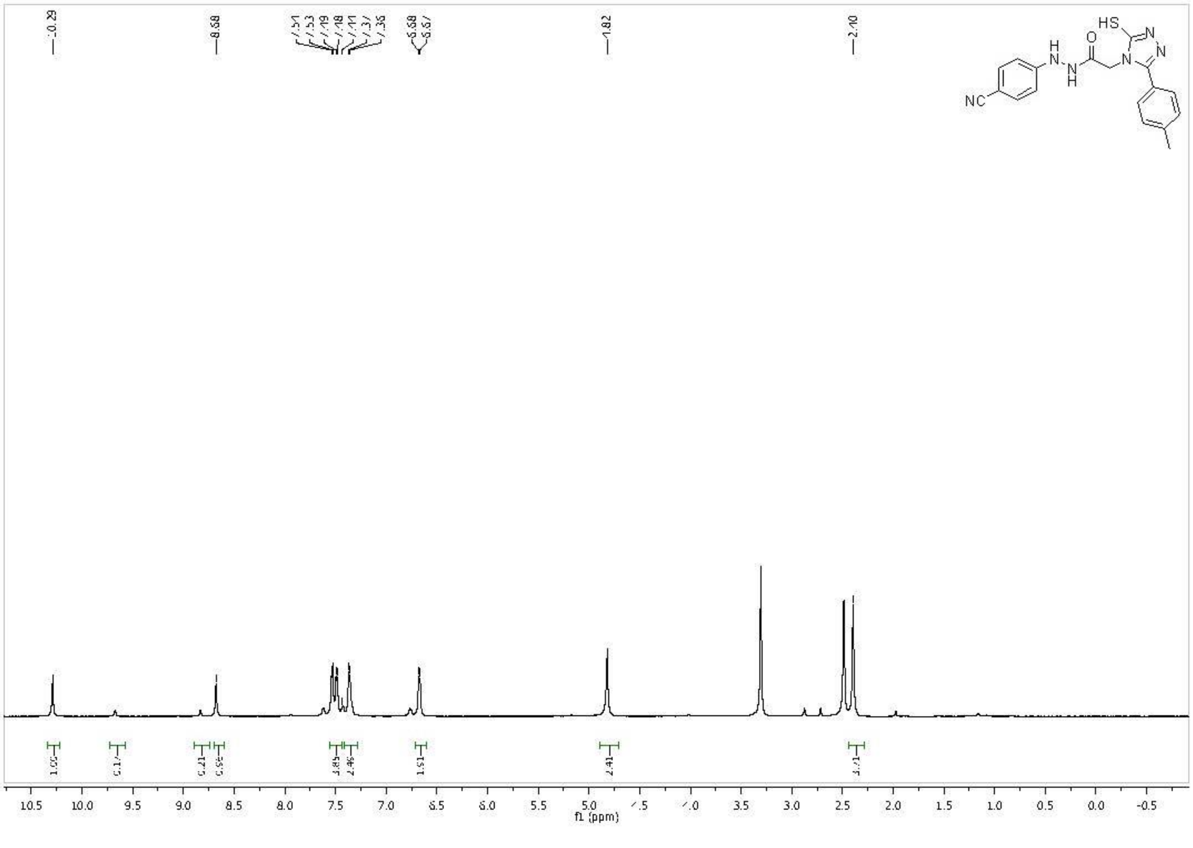
f1 (ppm)

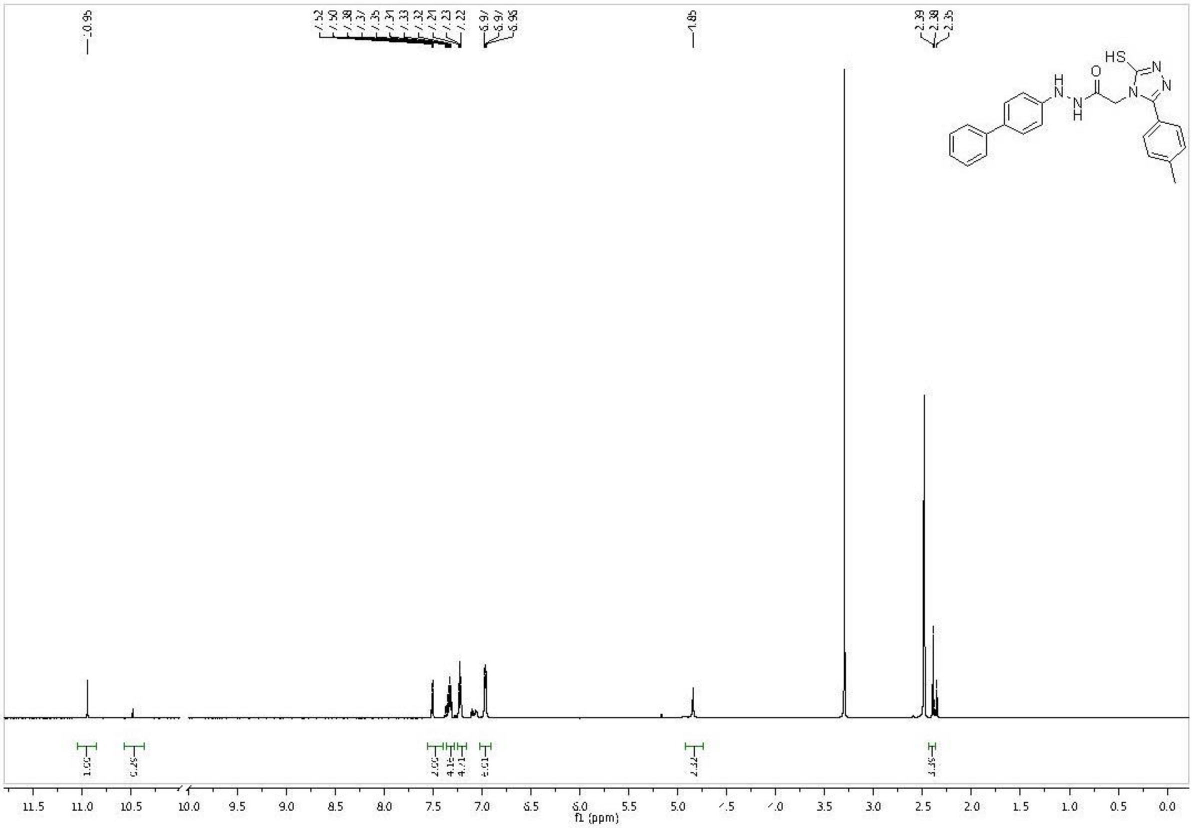

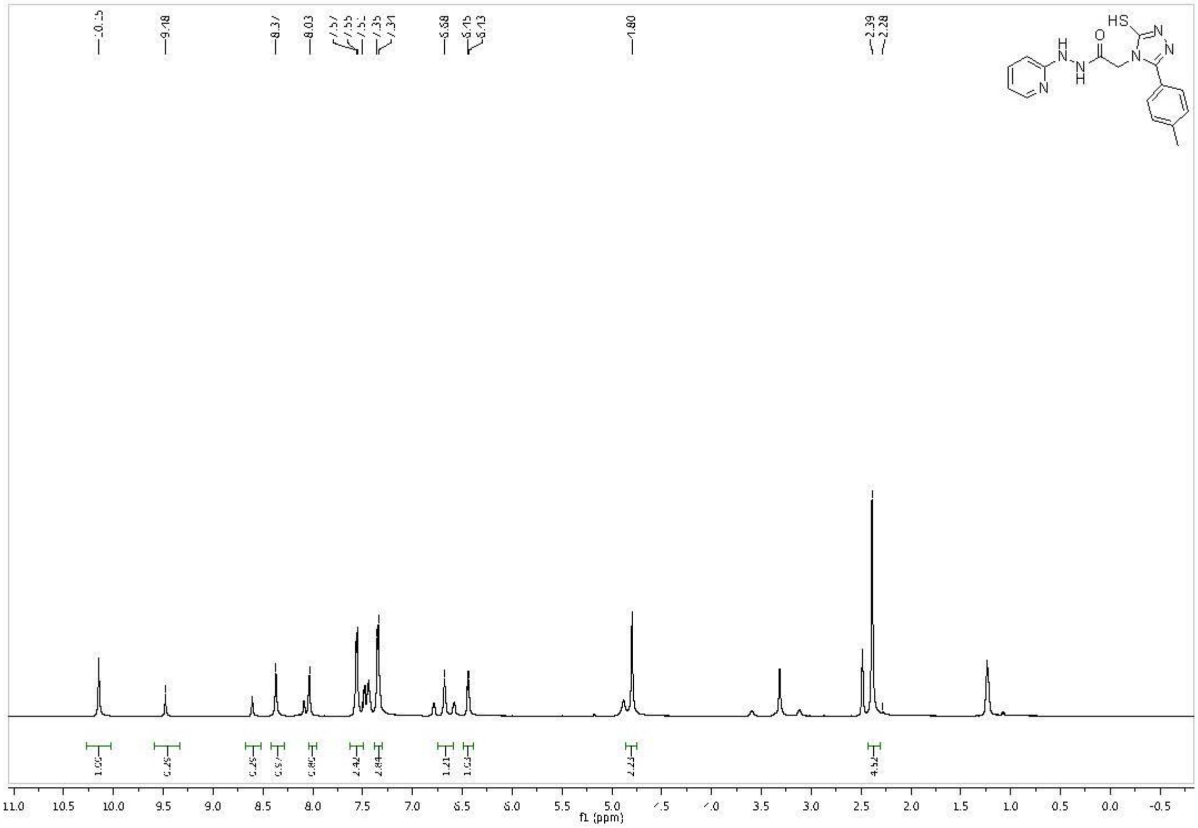

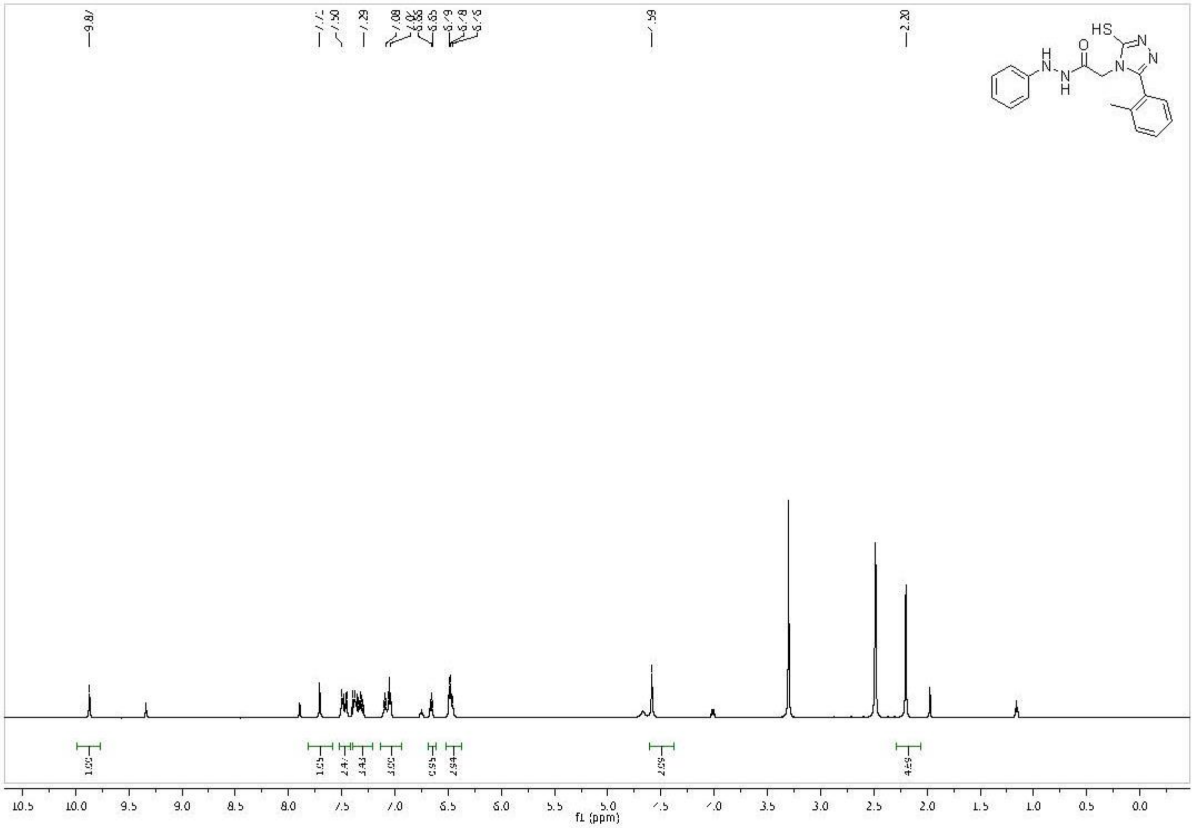

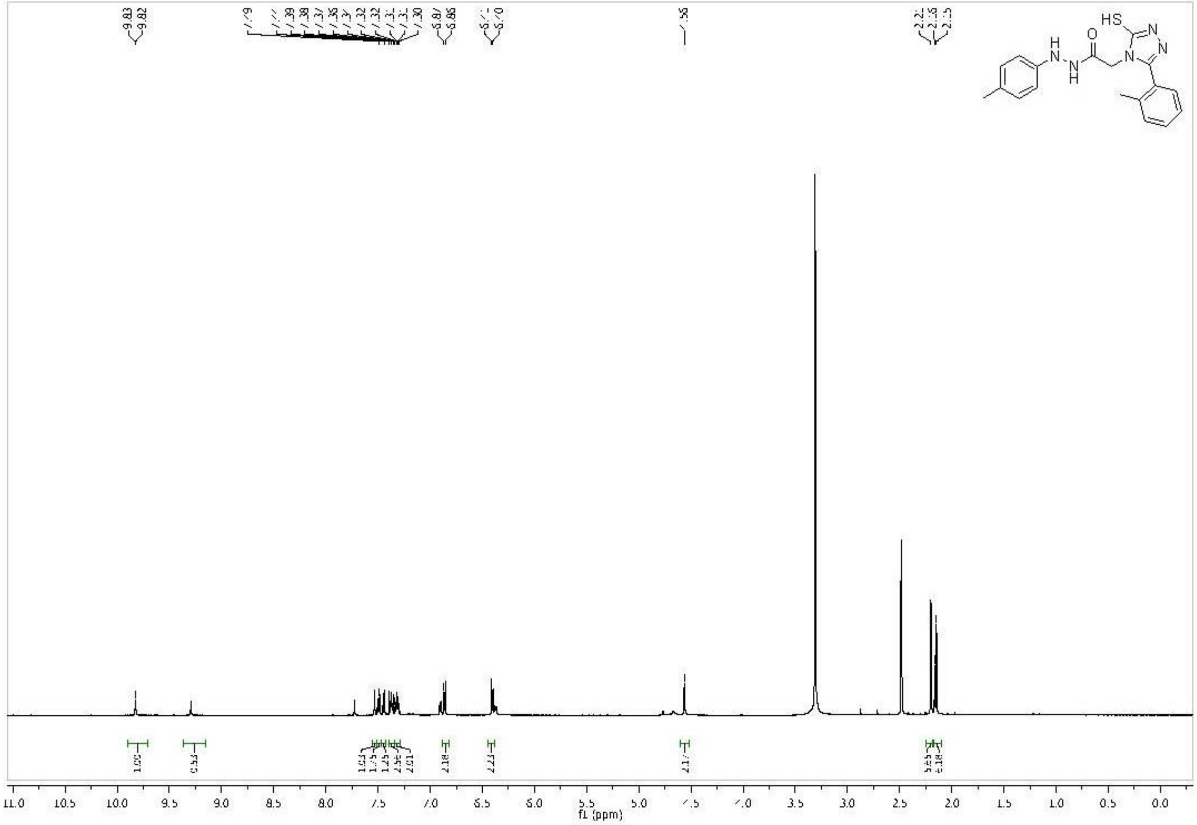

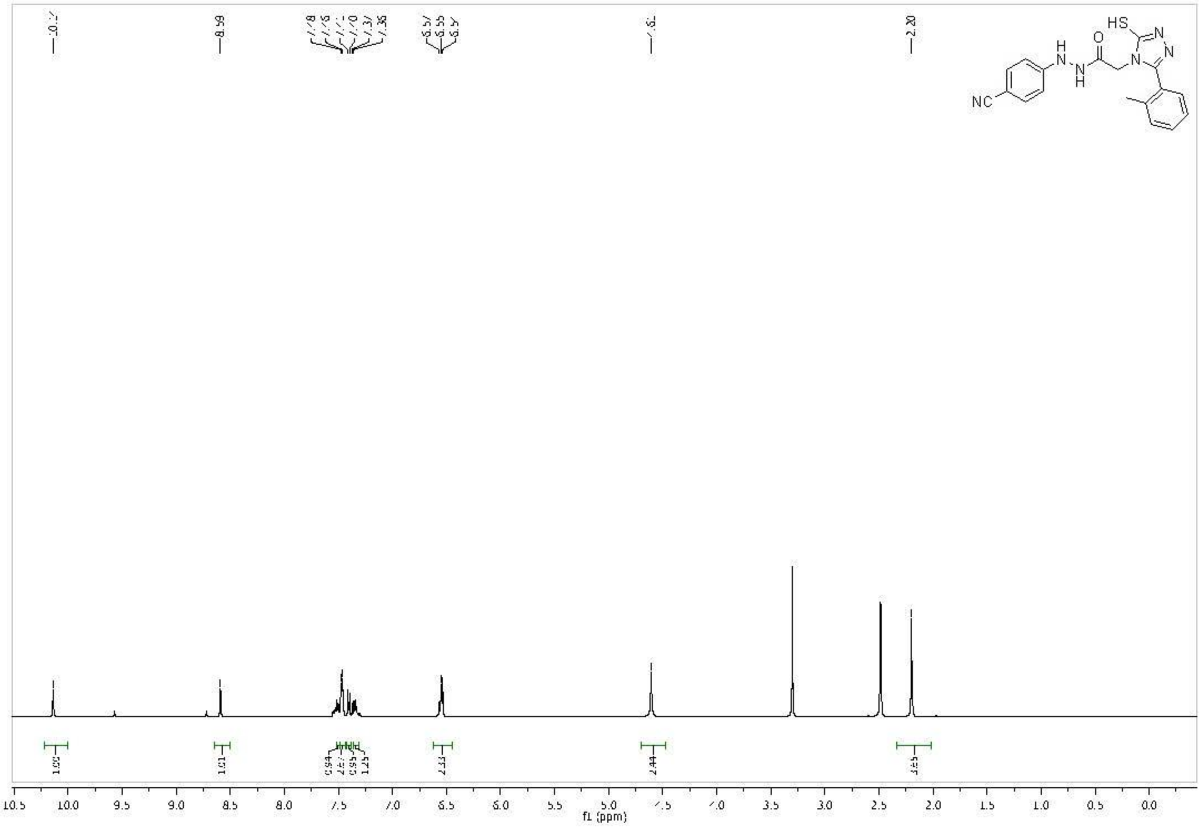

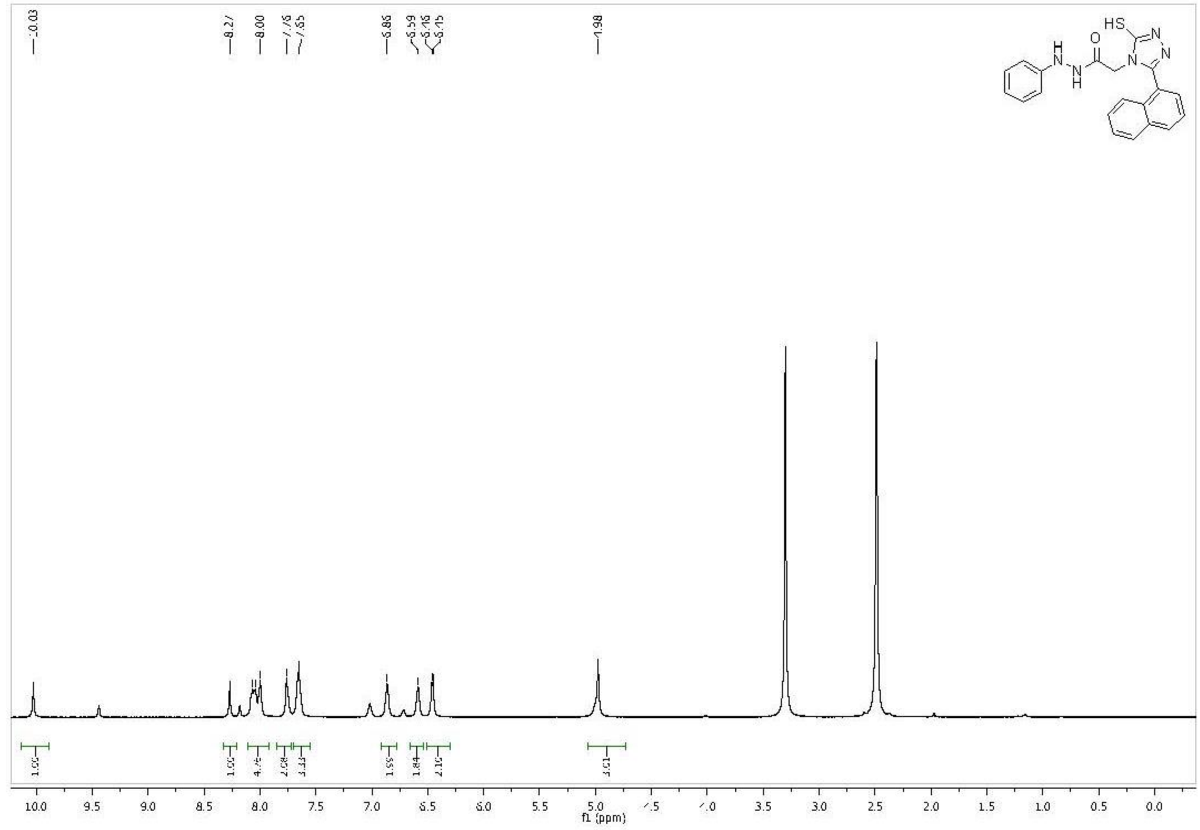

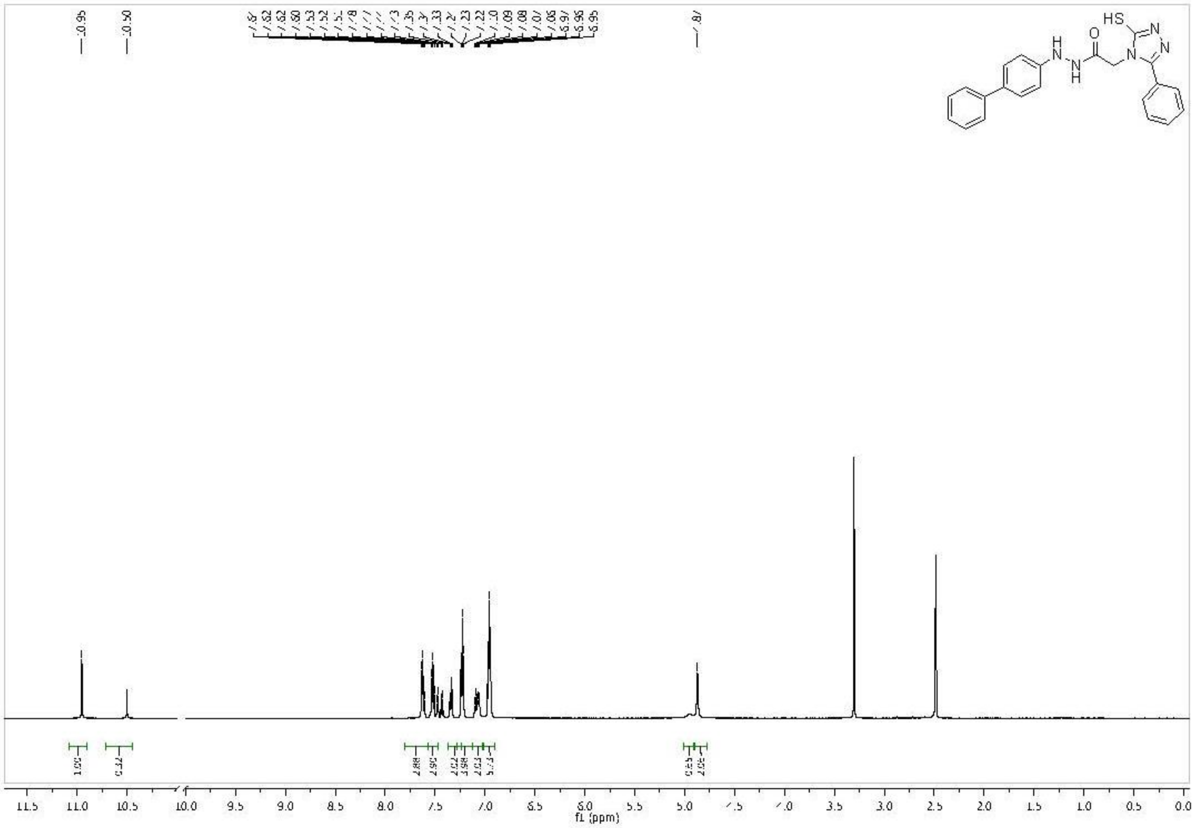

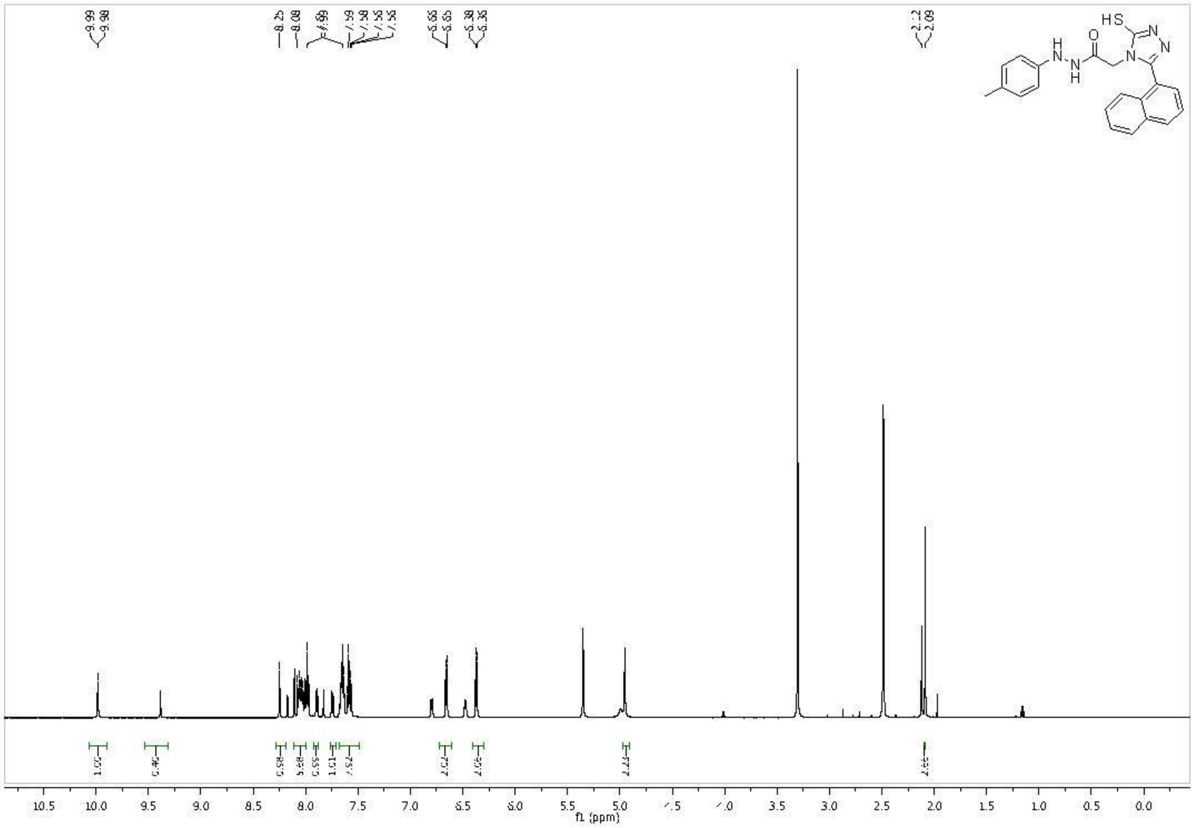

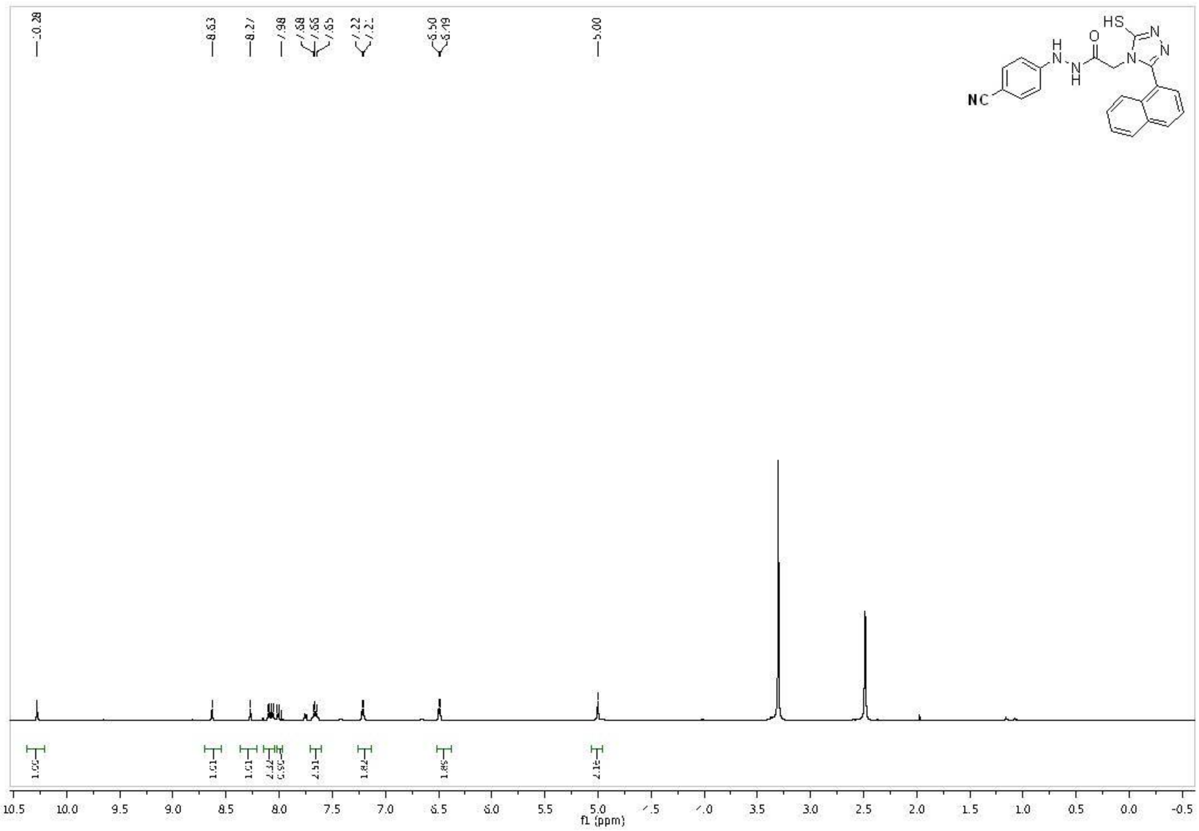
HS
NC
f1 (ppm)

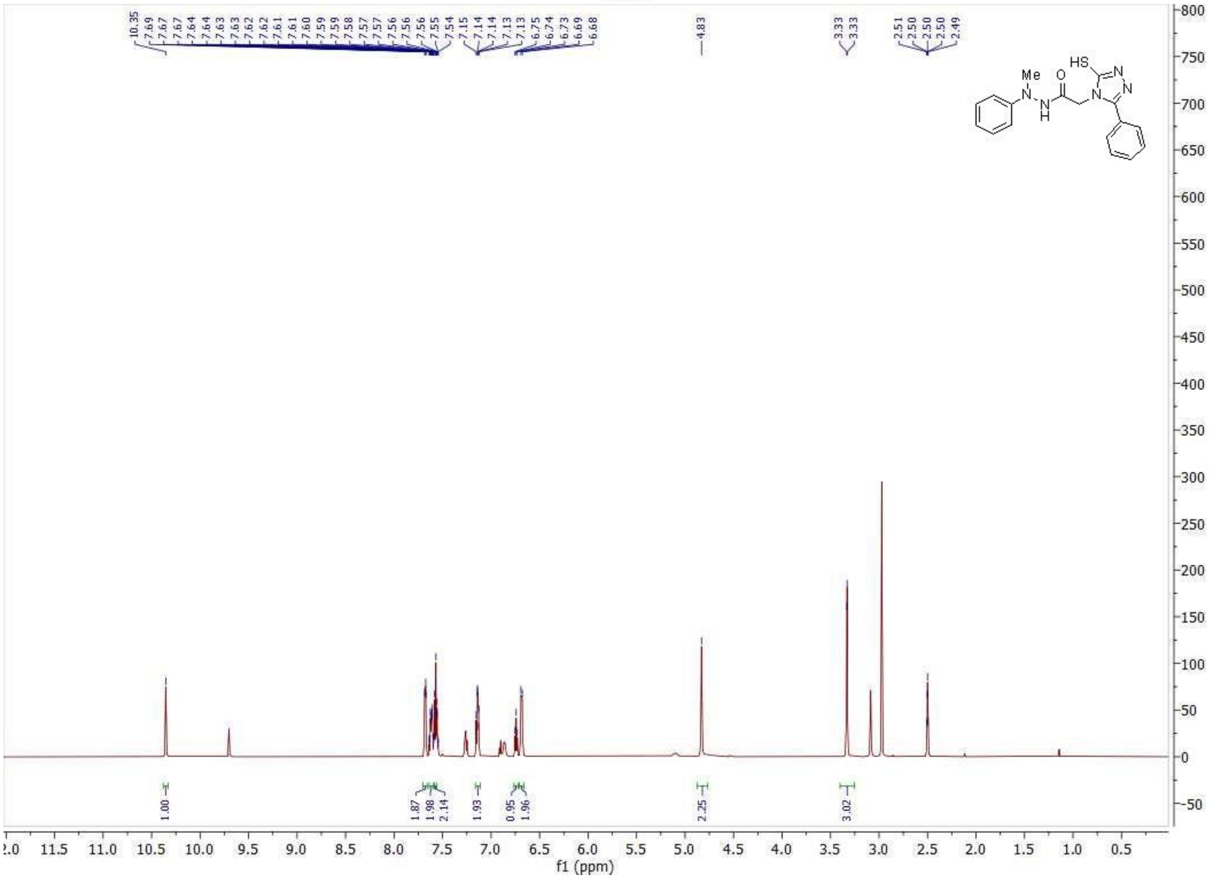
Me
HS
f1 (ppm)
